# Validity of observational evidence on putative risk and protective factors: appraisal of 3744 meta-analyses on 57 topics

**DOI:** 10.1186/s12916-021-02020-6

**Published:** 2021-07-06

**Authors:** Perrine Janiaud, Arnav Agarwal, Ioanna Tzoulaki, Evropi Theodoratou, Konstantinos K. Tsilidis, Evangelos Evangelou, John P. A. Ioannidis

**Affiliations:** 1 Meta-Research Innovation Center at Stanford (METRICS), Stanford, CA 94305 USA; 2 Department of Clinical Research, University Hospital Basel, University of Basel, CH-4056 Basel, Switzerland; 3 Department of Medicine, University of Toronto, 1 King’s College Circle #3172, Toronto, ON M5S 1A8 Canada; 4 Department of Hygiene and Epidemiology, University of Ioannina School of Medicine, University Campus, 45110 Ioannina, Greece; 5 Department of Epidemiology and Biostatistics, School of Public Health, Imperial College London, London, W2 1PG UK; 6 Centre for Global Health, The University of Edinburgh, Edinburgh, EH8 9AG UK; 7 Cancer Research UK Edinburgh Centre, Institute of Genetics and Cancer, Western General Hospital, The University of Edinburgh, Edinburgh, EH4 2XU UK; 8 Department of Epidemiology and Population Health, Stanford University School of Medicine, Stanford, CA 94305 USA; 9 Stanford Prevention Research Center, Department of Medicine, Stanford University School of Medicine, Stanford, CA 94305 USA; 10 Department of Biomedical Data Science, Stanford University School of Medicine, Stanford, CA 94305 USA; 11 Department of Statistics, Stanford University School of Humanities and Sciences, Stanford, CA 94305 USA

**Keywords:** Umbrella review, Observation studies, Randomized clinical trials, Mendelian randomization

## Abstract

**Background:**

The validity of observational studies and their meta-analyses is contested. Here, we aimed to appraise thousands of meta-analyses of observational studies using a pre-specified set of quantitative criteria that assess the significance, amount, consistency, and bias of the evidence. We also aimed to compare results from meta-analyses of observational studies against meta-analyses of randomized controlled trials (RCTs) and Mendelian randomization (MR) studies.

**Methods:**

We retrieved from PubMed (last update, November 19, 2020) umbrella reviews including meta-analyses of observational studies assessing putative risk or protective factors, regardless of the nature of the exposure and health outcome. We extracted information on 7 quantitative criteria that reflect the level of statistical support, the amount of data, the consistency across different studies, and hints pointing to potential bias. These criteria were level of statistical significance (pre-categorized according to 10^−6^, 0.001, and 0.05 *p*-value thresholds), sample size, statistical significance for the largest study, 95% prediction intervals, between-study heterogeneity, and the results of tests for small study effects and for excess significance.

**Results:**

3744 associations (in 57 umbrella reviews) assessed by a median number of 7 (interquartile range 4 to 11) observational studies were eligible. Most associations were statistically significant at *P* < 0.05 (61.1%, 2289/3744). Only 2.6% of associations had *P* < 10^−6^, ≥1000 cases (or ≥20,000 participants for continuous factors), *P* < 0.05 in the largest study, 95% prediction interval excluding the null, and no large between-study heterogeneity, small study effects, or excess significance. Across the 57 topics, large heterogeneity was observed in the proportion of associations fulfilling various quantitative criteria. The quantitative criteria were mostly independent from one another. Across 62 associations assessed in both RCTs and in observational studies, 37.1% had effect estimates in opposite directions and 43.5% had effect estimates differing beyond chance in the two designs. Across 94 comparisons assessed in both MR and observational studies, such discrepancies occurred in 30.8% and 54.7%, respectively.

**Conclusions:**

Acknowledging that no gold-standard exists to judge whether an observational association is genuine, statistically significant results are common in observational studies, but they are rarely convincing or corroborated by randomized evidence.

**Supplementary Information:**

The online version contains supplementary material available at 10.1186/s12916-021-02020-6.

## Background

The validity of observational studies of putative risk or protective factors is a subject of continuous debate. Critics focus on the weaknesses of the observational evidence and occasionally debates get further fueled by comparisons against other designs, in particular randomized trials. Usually debates address either single research questions or few associations [1, 2]. However, now we have the opportunity to assess systematically collected and synthesized evidence from thousands of observational associations. In the last decade, numerous umbrella reviews have summarized systematically the evidence from meta-analyses of observational epidemiological studies across entire fields of research [3, 4]. Umbrella reviews also typically assess the observational evidence by looking at the level of statistical support (statistical significance of results), the amount of data, the consistency across different studies, and hints pointing to potential bias. A series of seven standardized quantitative criteria (Table 1 and Additional file 1: Appendix Method 1) have been previously proposed and are commonly used [3–6].

**Table 1:** The seven standardized criteria

Levels of evidence	Description
Convincing	• Associations with a statistical significance at *P* < 10^−6^ • More than 1000 cases included (or more than 20,000 participants for continuous outcomes) • The largest component study reporting a significant result at *P* < 0.05 • A 95% prediction interval that excluded the null • Absence of large heterogeneity (I2<50%) • No evidence of small study effects (*P* > 0.10) • No evidence of excess significance (*P* > 0.10)
Highly suggestive	• Associations with a statistical significance at *P* < 10^−6^ • More than 1000 cases included (or more than 20,000 participants for continuous outcomes) • The largest component study reporting a significant result at *P* < 0.05.
Suggestive	• Associations with a statistical significance at *P* < 0.001 • More than 1000 cases included (or more than 20,000 participants for continuous outcomes).
Weak	• Associations with a statistical significance at *P* < 0.05

Previous umbrella reviews have used various criteria to assess the evidence from meta-analysis of observational epidemiological studies. The combination of these criteria allows to tentatively classify evidence from meta-analyses of statistically significant risks and protective factors into four levels described below. A more detailed description of the criteria can be found in Additional file 1: Appendix Method 1

Some of these umbrella reviews have also included systematic assessments of meta-analyses of randomized controlled trials (RCTs) and of Mendelian randomization (MR) studies (an alternative way to generate an equivalent to randomization under certain assumptions using genetic instruments) [7]. Juxtaposing observational and randomized evidence may allow to corroborate results and probe causality.

Here, we overview the evidence obtained from 3744 meta-analyses of observational studies included in umbrella reviews evaluating putative risk or protective non-genetic factors. We evaluate how these meta-analyses of observational studies perform on different quantitative criteria that address statistical significance, amount of evidence, consistency, and hints of bias. We also assess the concordance of observational epidemiological data against corresponding meta-analyses of RCTs and MR studies.

## Methods

### Data sources and searches

We systematically searched PubMed, up to November 19, 2020, for studies labeled as umbrella reviews in their title: umbrella [Title] AND review [Title]. The protocol has been registered on the Open Science Framework [8].

### Study selection

All umbrella reviews including meta-analyses of observational studies assessing putative risk or protective factors were eligible. We considered all putative factors (i.e., any attributes, characteristics, or exposure of an individual [9] that may either increase or decrease the occurrence of any type of health outcomes). Umbrella reviews not assessing any putative risk or protective factors in observational settings or not using any of the seven previously proposed standardized criteria (Table 1) to assess the evidence were excluded. One author (PJ) screened all resulting articles from the literature search for inclusion criteria and consulted with a second author (JPA) when in doubt. If two or more umbrella reviews had 50% of their associations (i.e., a putative risk or protective factor with a health outcome) assessed overlapping, we retained the one with the largest number of associations.

### Data extraction

At the umbrella reviews level, we abstracted data regarding study design (observational studies alone or combined with other study types); number of factors evaluated by study design, when available; methodological quality tool used (e.g., AMSTAR [10]); and method used to evaluate the evidence (i.e., the seven standardized criteria [3–6] or other method).

We then extracted the following data for each meta-analysis included in each umbrella review examining the association of a putative risk or protective factor with a health outcome: exposure, outcome, study designs included (e.g., cohort or case-control studies), number of included studies, participants, metric used (e.g., odds ratio, risk ratio, hazard ratio, mean difference, standardized mean difference), and data necessary for the evaluation of the pre-specified seven standardized criteria (Table 1). Data extraction was repeated limited to data from prospective cohort studies. When cohorts were mentioned, without specification of whether these were prospective or retrospective, we kept these data and then excluded them in separate sensitivity analyses.

For umbrella reviews that also separately considered RCTs and MR studies besides the observational association studies, we extracted the effect size and corresponding 95% confidence interval, total number of participants, number of cases/events, and genetic instruments used for MR studies. We did not perform any new quality assessment and relied on the ones performed by umbrella review authors. Two authors (PJ and AA) independently extracted 20% of the included umbrella reviews, while the rest was split between them. Discrepancies were resolved through consensus.

### Data synthesis and analysis

We started by reassessing the evidence for each association using the pre-specified list of criteria presented in Table 1 (more details in Additional file 1). In case of missing data, the criterion was considered as failed. The number and proportions of associations fulfilling each criterion and meeting the different levels of evidence (i.e., convincing, highly suggestive, suggestive, and weak) based on their combination (Table 1) were counted for each umbrella reviews (also labeled as topic). For each level of evidence, proportions were summarized across umbrella reviews using the restricted maximum likelihood random effects model meta-analysis and the arcsine transformation to normalize and stabilize the variance [11]. Similarly, proportions were summarized for each criterion but focusing solely on statistically significant associations (those with *P* < 0.05 for the random effects summary effect). The between-umbrella heterogeneity was estimated using I^2^ [12].

The concordance between the 7 criteria was assessed by Cohen’s kappa (κ), where a κ<0.6 represents weak agreement [13, 14]. First, we estimated the different κ across all umbrella reviews, including only statistically significant associations (those with *P* < 0.05 for the random effects summary effect). We then estimated the different κ and their corresponding confidence intervals within each umbrella review and combined them using random effects [15].

In previously published umbrellas, when all 7 criteria are met (*P* < 10^−6^, ≥1000 cases (or ≥20,000 participants for continuous factors), *P* < 0.05 in the largest study, 95% prediction interval excluding the null [16, 17], and no large between-study heterogeneity, small study effects [18–20], or excess significance [21–23]), the evidence has been called “convincing” [3–6] since there is strong statistical support, large amount of evidence, consistency, and no overt signals in the bias tests. We should acknowledge, however, that there is no gold standard of what constitutes a genuine risk or protective factor. Some convincing associations may have some other problem in their evidence that invalidates them. Conversely, other associations that are not mapped as convincing may well be true. Allowing for this uncertainty, we tried to address which criteria were the most constraining to reach a convincing level of evidence, as each criterion was separately removed from being required to have an association called convincing. This analysis was performed only on statistically significant associations for which information on all seven criteria was available. Numbers of additional associations reaching a convincing level of evidence were recorded. In addition to testing the different criteria, different statistical thresholds (*P* < 0.001 and <0.05) were also tested as alternatives to the original convincing level of statistical significance (*P* < 10^−6^). We also recorded how evidence was impacted by restricting the assessment of associations to prospective cohorts.

For associations assessed both by meta-analyses of observational studies and by either meta-analyses of RCTs or MR studies, we compared the effect sizes and corresponding 95% confidence intervals. The estimates across different designs were paired according to outcome, exposure, comparison, and population. For RCTs, if there were more than one meta-analysis for the same topic, we retained the one with the largest number of studies included. For MR studies in case of multiple studies for one observational association, each study was compared with the corresponding meta-analysis of observational studies. We specifically examined if the direction and statistical significance of the associations were concordant with the direction and statistical significance of effects in meta-analyses of RCTs and MR studies. We considered the traditional *P* < 0.05 threshold of statistical significance and also the more recently adopted *P* < 0.005 [24].

Moreover, to investigate whether the difference between the meta-analyses estimates was beyond chance, Q tests were performed (*P* < 0.10) [25]. For ease of interpretation, we converted all weighted mean differences (WMDs) and standardized mean difference (SMDs) to odds ratio (OR) equivalents [26] and assumed that relative risks (RRs) and hazard ratios (HRs) were interchangeable with ORs (a reasonable assumption for mostly rare event rates and for a minority where event rates are substantial, the OR is substantially larger than the RR). We also checked how often OR estimates using the different designs differed by two-fold or more.

For factors with statistically significant results both in observational as well as RCTs or MR studies’ evidence (and thus have the most consistent support), we recorded the pattern of the seven pre-specified criteria in the meta-analyses of observational epidemiological data.

## Results

### Eligible umbrella reviews and meta-analyses of observational associations

**Fig. 1:**
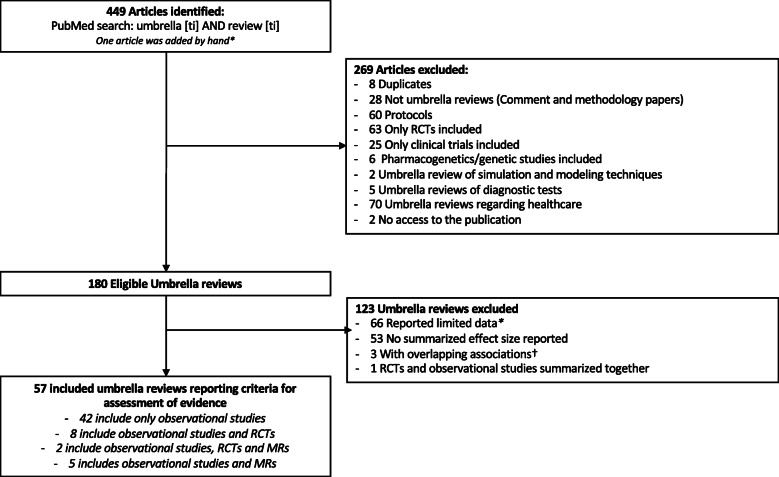
Flowchart of the literature search. *Umbrella reviews reported summarized effect sizes but did not report the other criteria of interest. ^†^Umbrella reviews assessing the same associations

The literature search yielded 449 articles of which 180 umbrella reviews were potentially eligible. Of those, 123 umbrella reviews were excluded as they had limited or inadequately reported data available, and did not use the seven standardized criteria to assess the evidence of their included associations or reported associations overlapped by over 50% with another umbrella review (Fig. 1).

**Fig. 2:**
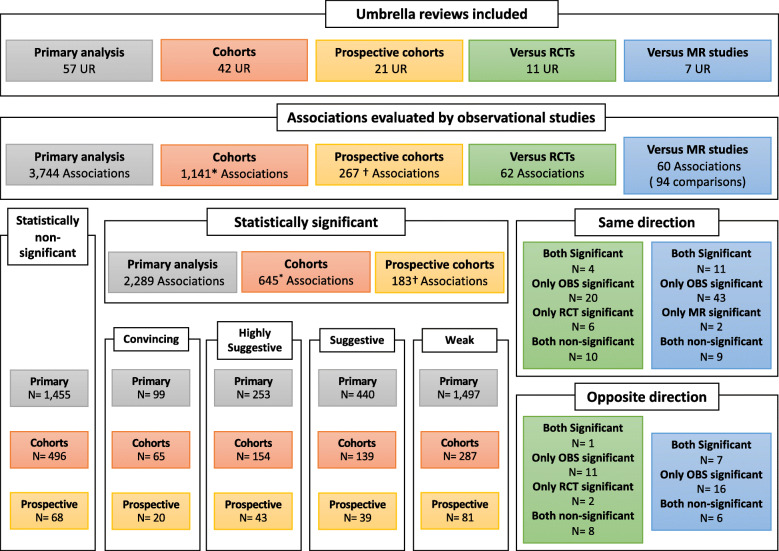
Overview of the included associations. MR, Mendelian randomization; OBS, observational studies; RCTs, randomized controlled trials. The statistical significance threshold was at *P* < 0.05. *Twenty-one of which were not assessable anymore as included only one cohort study per association. ^†^Sixteen of which were not assessable anymore as included only one cohort study per association

Fifty-seven umbrella reviews including 3744 associations assessed by meta-analyses of observational studies were included [5, 6, 27–81] (Fig. 2 and Table 2). The median number of estimates included in each meta-analysis was 7 (IQR 4 to 11) ranging from a minimum of 2 up to 309 estimates.

**Table 2:** Overview of the included umbrella reviews

Topic	First author	Year	Type of studies	N total studies ^**a**^	Median [IQR] Min to max.^**b**^	N total associations^**c**^	N associations included	Convincing	Highly suggestive	Suggestive	Weak	Non-significant
**One exposure with multiple outcomes**												
Adiposity and cancer outcomes	Kyrgiou	2017	OBS	507	6 [4; 11.50] (2 to 44)	194	67^d^	8 (11.9%)	14 (20.9%)	15 (22.4%)	11 (16.4%)	19 (28.4%)
Antidepressant and adverse events	Dragioti	2019	OBS	1012	6 [4; 11.25] (2 to 44)	120	120	3 (2.5%)	8 (6.7%)	24 (20%)	38 (31.7%)	47 (39.2%)
Antipsychotics and life-threatening events	Papola	2019	OBS	68	9 [6.75; 12.75] (6 to 24)	6	6	1 (16.7%)	2 (33.3%)	3 (50%)	0 (0%)	0 (0%)
Low-dose aspirin and health outcomes	Veronese	2020	OBS-RCT	NR	3.50 [3; 7.75] (2 to 32)	156	42	0 (0%)	0 (0%)	0 (0%)	11 (26.2%)	31 (73.8%)
Aspirin and cancer outcomes	Song	2020	OBS	NR	11 [7; 18.25] (3 to 309)	27	18^e^	0 (0%)	0 (0%)	0 (0%)	12 (66.7%)	6 (33.3%)
Birth weight and later life events	Belbasis	2016	OBS	NR	10 [6.25; 16] (3 to 45)	78	78	3 (3.8%)	8 (10.3%)	10 (12.8%)	29 (37.2%)	28 (35.9%)
Chocolate and health outcomes	Veronesem	2018	OBS-RCT	NR	5 [4; 6] (4 to 6)	19	7	0 (0%)	0 (0%)	0 (0%)	4 (57.1%)	3 (42.9%)
Chronic kidney disease and mortality	Kim	2020	OBS-RCT	NR	9 [4; 14] (2 to 26)	105	49	0 (0%)	0 (0%)	22 (44.9%)	11 (22.4%)	16 (32.7%)
Coffee and cancer risk	Zhao	2020	OBS	448	15 [7; 21.25] (4 to 54)	36	36	0 (0%)	3 (8.3%)	7 (19.4%)	7 (19.4%)	19 (52.8%)
C-reactive protein and health outcomes	Markozannes	2020	OBS-MR	952	6 [4; 11] (3 to 53)	309	113	2 (1.8%)	12 (10.6%)	14 (12.4%)	67 (59.3%)	18 (15.9%)
Depression and mortality	Machado	2018	OBS	246	6 [4; 12] (3 to 111)	17	17	0 (0%)	4 (23.5%)	2 (11.8%)	11 (64.7%)	0 (0%)
Antidepressants during pregnancy and neonatal outcomes	Biffi	2020	OBS	NR	7 [4; 10] (3 to 28)	69	69	0 (0%)	5 (7.2%)	11 (15.9%)	18 (26.1%)	35 (50.7%)
Dietary Fiber and health outcomes	Veronese	2018	OBS	NR	12 [6; 19] (3 to 26)	21	21	2 (9.5%)	1 (4.8%)	9 (42.9%)	6 (28.6%)	3 (14.3%)
Fish and ω-3 Fatty Acids consumptions and cancer outcomes	Lee	2020	OBS	NR	5 [3; 10] (2 to 17)	57	52^f^	0 (0%)	0 (0%)	2 (3.8%)	10 (19.2%)	40 (76.9%)
Influenza vaccine in elderly and health outcomes	Demurtas	2020	OBS-RCT	NR	3.50 [2; 6.75] (2 to 27)	60	38	1 (2.6%)	3 (7.9%)	6 (15.8%)	15 (39.5%)	13 (34.2%)
Handgrip strength and health outcomes	Soysal	2020	OBS	NR	9 [8; 10.50] (7 to 34)	11	8^e^	0 (0%)	1 (12.5%)	1 (12.5%)	4 (50%)	2 (25%)
Human immunodeficiency virus infections and health outcomes	Grabovac	2019	OBS	NR	8 [4; 13.50] (2 to 43)	55	55	0 (0%)	0 (0%)	9 (16.4%)	30 (54.5%)	16 (29.1%)
Metformin and cancer outcomes	Yu	2019	OBS	327	7 [5; 15] (2 to 29)	33	33	1 (3%)	3 (9.1%)	5 (15.2%)	14 (42.4%)	10 (30.3%)
Magnesium and health outcomes	Veronese	2019	OBS-RCT	NR	6 [3; 9.50] (3 to 32)	55	19	0 (0%)	0 (0%)	2 (10.5%)	7 (36.8%)	10 (52.6%)
Obesity and gynecology/obstetric outcomes	Kalliala	2017	OBS-RCT	427	6 [3; 9] (2 to 40)	248	144	11 (7.6%)	28 (19.4%)	23 (16%)	42 (29.2%)	40 (27.8%)
Physical activity and cancer outcomes	Rezende	2017	OBS	297	6 [3.25; 10] (2 to 38)	46	46	1 (2.2%)	2 (4.3%)	5 (10.9%)	5 (10.9%)	33 (71.7%)
Physical activity and atrial fibrillation outcomes	Valenzuela	2020	OBS	NR	8 [7; 19] (6 to 20)	5	5	0 (0%)	0 (0%)	0 (0%)	3 (60%)	2 (40%)
Statins and multiple non-cardiovascular outcomes	Yazhou	2018	OBS-RCT	NR	6 [4; 9] (2 to 27)	278	115	0 (0%)	2 (1.7%)	21 (18.3%)	42 (36.5%)	50 (59.8%)
Serum uric acid and health outcomes	Li	2017	OBS-RCT-MR	NR	5 [3; 9] (2 to 31)	152	76	0 (0%)	7 (9.2%)	9 (11.8%)	41 (53.9%)	19 (25%)
Type 2 diabetes mellitus and cancer	Tsilidis	2015	OBS	474	14 [9; 21] (5 to 45)	27	27	2 (7.4%)	4 (14.8%)	4 (14.8%)	10 (37%)	7 (25.9%)
Tea consumption and cancer	Kim	2020	OBS	NR	10 [6.75; 16] (4 to 53)	150	68^g^	0 (0%)	1 (1.5%)	2 (2.9%)	18 (26.5%)	47 (69.1%)
Telomere length and health outcomes	Smith	2019	OBS	NR	5.50 [3; 8] (2 to 20)	50	50	0 (0%)	1 (2%)	0 (0%)	23 (46%)	26 (52%)
Vitamin D and health outcomes	Theodoratou	2014	OBS-RCT	NR	7 [5; 10] (2 to 37)	48	48	0 (0%)	6 (12.5%)	7 (14.6%)	16 (33.3%)	19 (39.6%)
**Multiple exposures with one outcome**												
Risk factor for attention deficit hyperactivity disorder	Kim	2020	OBS	NR	6 [4; 9] (2 to 30)	63	63	5 (7.9%)	3 (4.8%)	11 (17.5%)	26 (41.3%)	18 (28.6%)
Risk and protective factors for mental disorders with onset in childhood/adolescence	Marco	2020	OBS	192	6 [4.50; 9] (2 to 26)	23	23	0 (0%)	0 (0%)	1 (4.3%)	8 (34.8%)	14 (60.9%)
Environmental factors and serum biomarkers for atrial fibrillation	Belbasis	2020	OBS	NR	6 [4; 8] (3 to 31)	51	51	6 (11.8%)	11 (21.6%)	8 (15.7%)	10 (19.6%)	16 (31.4%)
Factors associated to loneliness	Solmi	2020	OBS	NR	13 [8; 18] (3 to 31)	5	5	0 (0%)	0 (0%)	1 (20%)	4 (80%)	0 (0%)
Risk factor for amyotrophic lateral sclerosis	Belbasis	2016	OBS	NR	8 [5.75; 9.25] (3 to 20)	16	16	0 (0%)	0 (0%)	3 (18.8%)	6 (37.5%)	7 (43.8%)
Risk and protective factors for anxiety and obsessive compulsive disorders	Fullana	2019	OBS	216	3 [2; 6] (2 to 112)	427	128^fg^	4 (3.1%)	2 (1.6%)	3 (2.3%)	60 (46.9%)	59 (46.1%)
Environmental risk factors and biomarkers for autism spectrum disorder	Kim	2019	OBS	NR	8 [3.50; 13] (2 to 24)	67	67	8 (11.9%)	7 (10.4%)	11 (16.4%)	26 (38.8%)	15 (22.4%)
Environmental risk factors for bipolar disorder	Bortolato	2017	OBS	54	8 [5; 10] (3 to 13)	7	7	1 (14.3%)	1 (14.3%)	2 (28.6%)	2 (28.6%)	1 (14.3%)
Risk factors for colorectal cancer metastasis and recurrence	Xu	2020	OBS	NR	6 [3.50; 9] (2 to 41)	47	47	0 (0%)	0 (0%)	4 (8.5%)	27 (57.4%)	16 (34%)
Non-genetic biomarkers and colorectal cancer risk	Zhang	2020	OBS-RCT-MR	NR	7 [3; 10] (2 to 28)	112	65	0 (0%)	0 (0%)	4 (6.2%)	25 (38.5%)	36 (55.4%)
Risk factors for chronic obstructive pulmonary disease	Bellou	2019	OBS-MR	NR	5 [4; 11] (3 to 22)	22	18	0 (0%)	0 (0%)	8 (44.4%)	5 (27.8%)	5 (27.8%)
Environmental risk factors for dementia	Bellou	2017	OBS	NR	7 [4.75; 13] (3 to 43)	76	76	7 (9.2%)	5 (6.6%)	10 (13.2%)	33 (43.4%)	21 (27.6%)
Risk factors for depression	Kohler	2018	OBS-MR	NR	7.50 [5; 11] (3 to 77)	140	134	0 (0%)	0 (0%)	41 (30.6%)	57 (42.5%)	36 (26.9%)
Risk factors for eating disorders	Solmi	2020	OBS	NR	6 [4; 9] (2 to 33)	49	49	0 (0%)	0 (0%)	6 (12.2%)	35 (71.4%)	8 (16.3%)
Risk factors for endometrial cancer	Raglan	2019	OBS	604	4 [3; 6] (2 to 28)	127	127	3 (2.4%)	13 (10.2%)	14 (11%)	26 (20.5%)	71 (55.9%)
Environmental risk factors for obesity	Solmi	2018	OBS-RCT	166	8 [6; 10.75] (2 to 22)	60	26	4 (15.4%)	2 (7.7%)	1 (3.8%)	15 (57.7%)	4 (15.4%)
Prognostic biomarkers for gastric cancer	Zhou	2019	OBS	>1000	7 [4; 11] (3 to 51)	119	119	3 (2.5%)	7 (5.9%)	3 (2.5%)	82 (68.9%)	24 (20.2%)
Risk factors for gestational diabetes	Giannakou	2019	OBS	NR	8 [5; 14] (3 to 40)	61	61	1 (1.6%)	13 (21.3%)	9 (14.8%)	28 (45.9%)	10 (16.4%)
Peripheral biomarkers and major mental disorders	Carvalho	2020	OBS	NR	7 [5; 13] (3 to 55)	358	318^g^	0 (0%)	0 (0%)	3 (0.9%)	175 (55%)	140 (44%)
Environmental risk factors for multiple sclerosis	Belbasis	2015	OBS	NR	8 [6; 12] (3 to 30)	44	44	2 (4.5%)	2 (4.5%)	2 (4.5%)	17 (38.6%)	21 (47.7%)
Prognostic biomarkers for pancreatic ductal adenocarcinoma	Wang	2020	OBS	>300	4 [3; 6] (2 to 43)	63	63	0 (0%)	2 (3.2%)	1 (1.6%)	41 (65.1%)	19 (30.2%)
Environmental risk factors and Parkinson’s	Bellou	2016	OBS	755	7 [5; 10] (2 to 67)	75	75	2 (2.7%)	6 (8%)	9 (12%)	18 (24%)	40 (53.3%)
Risk and protective factors for prostate cancer	Markozannes	2016	OBS	1907	5 [3.75; 7] (2 to 45)	248	176^d^	0 (0%)	2 (1.1%)	7 (4%)	25 (14.2%)	142 (80.7%)
Non-genetic risk factors for pre-eclampsia	Giannakou	2017	OBS	NR	7 [4; 12.25] (3 to 34)	130	64^h^	1 (1.6%)	11 (17.2%)	5 (7.8%)	22 (34.4%)	25 (39.1%)
Risk and protective factors for psychosis	Ruada	2018	OBS	683	6 [3; 9] (2 to 55)	170	128^i^	1 (0.8%)	2 (1.6%)	11 (8.6%)	64 (50%)	50 (39.1%)
Environmental risk factors for rheumatic diseases	Belbasis	2018	OBS	NR	10.50 [7; 13] (3 to 51)	42	42	0 (0%)	0 (0%)	7 (16.7%)	26 (61.9%)	9 (21.4%)
Risk factors and peripheral biomarkers for schizophrenia spectrum disorders	Belbasis	2017	OBS-MR	NR	8 [5.25; 13] (3 to 42)	98	98	1 (1%)	4 (4.1%)	5 (5.1%)	52 (53.1%)	36 (36.7%)
Non-genetic risk factors for skin cancer	Belbasis	2016	OBS	NR	10 [7; 18] (3 to 41)	85	85	4 (4.7%)	9 (10.6%)	11 (12.9%)	34 (40%)	27 (31.8%)
Risk factors for type 2 diabetes mellitus	Bellou	2018	OBS-MR	NR	9.50 [6; 14] (3 to 88)	155	142	11 (7.7%)	34 (23.9%)	28 (19.7%)	43 (30.3%)	26 (18.3%)

*IQR* interquartile range, *MA* meta-analyses, *MR* Mendelian randomization, *NR* not reported, *OBS* observational studies, *OCD* obsessive and compulsive disorders, *RCT* randomized controlled trials

^a^Total number of primary studies included in the meta-analyses assessed by the umbrella reviews

^b^Median number IQR and minimum and maximum number of primary studies included in the associations assessed

^c^Total number of associations assessed in the included umbrella reviews

^d^The umbrella review presented data for continuous and binary outcomes but their principal analyses focused only on continuous outcomes which we included hence the lowest number of included associations in our work compared with the original umbrella review

^e^Some associations were excluded as they were assessed by a mix of RCTs and observational studies

^f^Associations assessed by only one study were removed

^g^Duplicated associations were excluded

^h^Excluded associations assessing genetic factors

^i^The authors of the umbrella review mention 170 associations but only report 145. Out of the 145, 17 meta-analyses were excluded because included only one study

### Assessment according to a set of 7 pre-specified quantitative criteria

Overall, 99 (2.6%) associations were graded as convincing, 253 (6.7%) as highly suggestive, 440 (11.8%) as suggestive, and 1497 (40.0%) as weak and 1455 (38.9%) were not statistically significant at *P* < 0.05 (Fig. 2). Meta-analyses of the proportions of convincing and highly suggestive associations across the 57 topics resulted in 1.3% (95% CI [1.0–2.2%]) summary proportion for convincing and 4.6% (95% CI [2.9–6.6%]) summary proportion for highly suggestive associations, and both had very high between-topic heterogeneity (I^2^ = 73.9% and I^2^ = 85.7%, respectively) (Table 3 and Additional file 2: Figures 1 to 5). Convincing associations varied from 0 to 16.7% across topics and 29/57 umbrella reviews had no associations with convincing evidence [6, 29, 36, 38, 40, 41, 45, 47, 50, 52, 53, 56, 58, 62, 63, 65, 66, 68–72, 74–76, 78–81] (Table 2). Moreover, the number of non-statistically significant associations (those with P≥0.05) varied substantially between topics from 0% for the associations of depression with mortality outcomes, antipsychotics with life-threatening events, and health factors with loneliness [40, 55, 68] to 80.7% for risk factors of prostate cancer [41].

**Table 3:** Meta-analyses of the proportions of associations for each criterion and level of evidence (random effects)

	n/N associations (crude proportion)	Proportions [95% CI] from meta-analysis of 57 topics^**a**^	I^**2**^	Range of proportions across topics
**Level of evidence:**				
Convincing	99/3744 (2.6%)	1.3% [1.0%; 2.2%]	73.9%	0–16.7%
Highly suggestive	253/3744 (6.7%)	4.6% [2.9%; 6.6%]	85.7%	0–33.3%
Suggestive	440/3744 (11.8%)	11.0% [8.5%; 13.8%]	83.9%	0–50%
Weak	1497/3744 (40.0%)	39.1% [34.8%; 43.5%]	86.2%	0–71.4%
Non-significant	1455/3744 (38.9%)	34.7% [29.2%; 40.3%]	90.8%	0–80.7%
**Criteria:**				
Statistical significance				
*P* < 10^-6^	762/2289 (33.3%)	29.0% [24.9%; 33.3%]	74.8%	0–66.7%
*P* < 0.001	1377/2289 (60.2%)	58.6% [54.1%; 63.0%]	73.3%	0–100%
Cases > 1000 (or > 20,000 participants for continuous outcomes)	1182/2107 (56.1%)	65.3% [56.9%; 73.2%]	94.9%	1.7–100%
Largest study with *P* < 0.05	1343/1781 (75.4%)	74.9% [71.2%; 78.4%]	63.6%	28.6–100%
95% prediction interval that excluded the null	642/2136 (30.1%)	30.3% [26.5%; 34.2%]	71.0%	9.0–100%
Absence of large heterogeneity (I^2^<50%)	1050/2277 (46.1%)	46.6% [41.8%; 51.3%]	79.5%	0–88.2%
No evidence of small study effects (*P* > 0.10)	1628/2164 (75.2%)	75.3% [72.2%; 78.3%]	63.2%	40–100%
No evidence of excess significance (*P* > 0.10)	1599/2052 (77.9%)	77.7% [72.6%; 82.5%]	83.0%	33.3–100%

^a^Meta-analyses for the individual criteria excluded associations with missing data. Meta-analyses for the levels of evidence were conducted across all 3744 associations regardless of their statistical significance status. The meta-analyses for the individual criteria were conducted across the 2289 statistically significant associations. Out of 2289, statistically significant associations, 182 associations did not report on the number of cases,508 on whether the largest study had *P* < 0.05, 153 on the 95% prediction interval, 12 on the I^2^ for heterogeneity, 125 on the small study effect test, and 237 on the excess of significance bias. Data on all 7 criteria were available for 1457 statistically significant meta-analyses

41.4% (1549/3744) of the associations had at least one missing criterion (Additional file 3: Figures 6 to 7). An additional 25 and 82 associations would have reached a convincing and highly suggestive level of evidence, respectively, if missing criteria were considered to be satisfied.

We performed meta-analyses for the proportion of associations that met each of the 7 pre-specified quantitative criteria across the 57 topics, limiting to the 2289 statistically significant associations. Only 29% (95% CI [24.9–33.3%]) of the associations had *P* < 10^−6^. Conversely, 74.9% (95% CI [71.2–78.4%]) of the associations had the largest study with *P* < 0.05, and 75.3% (95% CI [72.2–78.3%]) and 77.7% (95% CI [72.6–82.5%]) of the associations with available data had no signals of small-study effects or excess significance, respectively. Between-topic heterogeneity for the presence of each criterion was typically high (Table 3 and Additional file 4: Figures 8 to 15).

### Concordance between the 7 pre-specified quantitative criteria

There was a limited concordance between the different criteria, meaning that they provide mostly independent information (Fig. 3). Excluding the kappa coefficients for the concordance of different *P*-value thresholds, a weak to moderate concordance existed only between prediction intervals excluding the null and *P* < 10^−6^ (κ=0.44) (Additional file 5: Table 1 and Additional file 6: Figures 16 to 43).

**Fig. 3:**
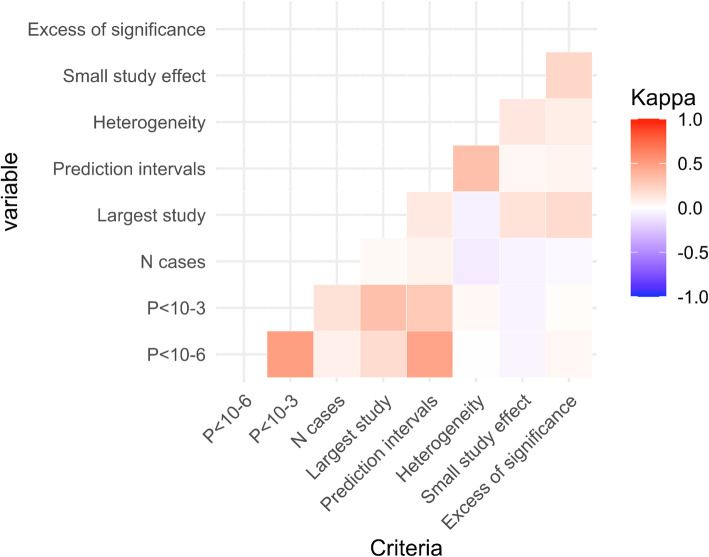
Kappa heatmap for the seven criteria across all umbrella reviews. Only statistically significant associations (with *P* < 0.05 for the random effects summary effect) were included in the Cohen’s kappa analysis. A κ<0.6 (lighter red) represents a weak, 0.6≤κ< 0.8 a moderate (red), and κ≥0.8 (dark red) a strong strength of agreement. Conversely, a κ>−0.6 represents a weak (light blue), −0.8<κ≤−0.6 a moderate (blue), and κ≤−0.8 (dark blue) a strong disagreement. The kappa estimated within each umbrella reviews and combined using random effects meta-analyses are presented eFigure 5 and eFigure 6

### Impact of each pre-specified criterion on number of associations deemed to have convincing evidence

1457 statistically significant associations (*P* < 0.05) had information available on all 7 criteria. Replacing the *P*-value threshold of <10^−6^ by <0.001 as a requirement for convincing evidence, convincing associations increased from 6.8 to 9.2% and increased even further to 9.7% when the threshold was set at <0.05 (Table 4). The most constraining criterion appeared to be the absence of large heterogeneity (I^2^ > 50%); removing it increased the number of convincing associations to 10.9%.

**Table 4:** Changes in number of associations that are graded as having convincing evidence when one criterion is dropped or replaced by a more lenient version

Credibility assessment	N associations *(total=1457)*^*a*^	Proportion
**Convincing**	**99**	**6.8%**
Replace *P* < 10^−6^ by <0.001	134	9.2%
Replace *P* < 10^−6^ by < 0.05	142	9.7%
Without the minimum number of cases criterion	149	10.2%
Without the largest study at *p* < 0.05 criterion	103	7.1%
Without the 95% prediction interval criterion	106	7.3%
Without the heterogeneity I^2^<50% criterion	159	10.9%
Without the small study effects criterion	122	8.4%
Without the excess significance criterion	111	7.6%

^a^These are the associations that are statistically significant (*P* < 0.05) and also have information on all criteria

### Analyses limited to prospective cohort studies

We were able to isolate 1141 associations which included only cohorts or where it was possible to separate cohort studies from other designs. Out of the 1141 associations, 849 were assessed by an unspecified mix of prospective and retrospective cohorts with no means to distinguish them from one another, 126 only by prospective cohort studies, 25 only by retrospective cohort studies, and 141 by a mix of study designs but where it was possible to separate the prospective cohort studies from the other designs. Across the 1141 associations, when limited to cohort studies, convincing associations decreased slightly from 5.7% (*n* = 65) to 4.2% (*n* = 48), and highly suggestive associations decreased from 13.5% (*n* = 154) to 11.7% (*n* = 133) (Fig. 2 and dataset available on the Open Science Framework [82]).

### Comparison against RCTs and MR studies

Only 16 out of the 57 umbrella reviews also investigated evidence from RCTs, [36, 37, 44, 45, 47, 52, 56, 69, 77], MR studies [27, 33, 38, 62, 73], or both [6, 71] in addition to observational studies. Of those 16, 5 had no overlapping associations between the different study designs [37, 44, 47, 52, 77] and one only provided a narrative summary of MR studies [62]. For 121 of the 882 observational associations evaluated in the 16 included umbrella reviews, evidence from 62 meta-analyses of RCTs or 60 MR studies could be juxtaposed; of note, one association was assessed both by a meta-analyses of RCTs and by a MR study. Nine observational associations were assessed by more than one MR study using different genetic instruments, thus resulting in a total of 94 comparisons. Results are presented in Fig. 2 and Additional file 7: Table 2 and Additional file 8: Table 3.

When comparing meta-analyses of observational studies against meta-analyses of RCTs, half of the associations (31/62) were only statistically significant in meta-analyses of observational studies (at the P < 0.05 level), while eight were only statistically significant in meta-analyses of RCTs. Four estimates were statistically significant with point estimates in the same direction for both types of design. Conversely, for one association, the point estimates were statistically significant, but in different direction, statins significantly increased the risk of pancreatitis (OR= 1.41, 95% CI [1.15; 1.74], *P* = 0.04) when limiting the evidence to the meta-analysis of observational studies but the risk was decreased in the meta-analysis of RCTs (OR=0.77, 95% CI [0.61; 0.97]) (36). Overall, 37.1% (23/62) of the estimates showed point estimates in the opposite direction in observational and RCT meta-analyses (Additional file 7: Table 2). When the *P* < 0.005 level was used, only two associations were statistically significant in both meta-analyses of observational studies and RCTs. The differences between the meta-analyses estimates of observational studies and RCTs were beyond chance for 43.5% (27/62) associations (*P* < 0.10 for the χ^2^ Q test) and 12.5% (8/64) differed in their effect sizes by two-fold or more in the two designs (Additional file 9: Figure 44 to 45).

Of 94 comparisons between meta-analyses of observational studies and MR studies, 62.8% (59/94) were solely statistically significant in observational studies (at the *P* < 0.05 level). Eleven comparisons showed a statistically significant evidence in both study designs with point estimates in the same direction. However, seven comparisons resulted in discordant results with statistically significant point estimates in opposite direction. Overall 30.8% (29/94) comparisons had point estimates in opposite directions between meta-analyses of observational studies and MR studies. Between MR studies, differences in the direction of the point estimates were also noted. For example, no significant associations were shown in MR studies between smoking and depression; however, the observational studies showed a significant increased risk of depression in smokers (OR= 1.68, 95% CI [1.55; 1.82]). All MR studies’ point estimates were in the same direction (increased risk) except for one (OR=0.85, 95% CI [0.66; 1.1]) [38] (Additional file 8: Table 3). When using the *P* < 0.005 level for claiming statistical significance, only seven out of 18 associations remained statistically significant in MR studies. When comparing the meta-analysis’ effects in observational studies versus the MR studies, there were significant heterogeneity (P < 0.10 for the χ^2^ Q test) between the two designs for 54.7% (54/94) comparisons and 12 (12.8%) differed by two-fold or more in their effect sizes (Additional file 9: Figures 44 to 45).

Overall, only four associations assessed by observational studies and RCTs and another three comparisons assessed by observational and MR studies had consistently statistically significant results (*P* < 0.05) in the same direction. Of these seven associations, the seven pre-specified criteria had graded two of them as highly suggestive, two as suggestive and three as weak.

## Discussion

We assessed the evidence obtained from observational studies for associations on 3744 putative risk and protective factors assessed by a median of 7 (IQR 4 to 11) estimates per meta-analysis from 57 umbrella reviews on diverse topics. Although the majority (61.1%) of the investigated associations were statistically significant at the traditional *P* < 0.05 level, only 2.6% and 6.7% were classified as having convincing or highly suggestive evidence, respectively, using a set of pre-specified criteria that have been used in the literature of umbrella reviews [3–6]. The proportions of associations meeting the various pre-specified criteria of statistical significance, amount of evidence, consistency, and lack of hints for bias and reaching different level of evidence varied across topics. Variability was highly prominent for the proportion of probed associations that had non-statistically significant (P≥0.05) results (0–80.7%).

The seven criteria that have been previously used to assess evidence from meta-analyses of observational associations have been developed ad hoc [3–6] aiming to capture sufficient statistical support, amount of evidence, consistency, and lack of signals that may herald bias [12, 20, 21]. It is unknown how well they can really identify convincing/strong evidence, let alone causality. A perfect gold standard is missing for causality in observational associations. Nevertheless, we could assess here the performance of these criteria against each other. They mostly showed low concordance among themselves and thus may offer relatively independent, complementary insights into the evidence of an observational association. Most associations did not offer any signal of small-study effects and excess significance. However, these results are to be interpreted with caution since both tests are not definite proof of presence or absence of bias; given the typically small or modest number of studies in each meta-analysis the power of these tests is very limited [83]. Conversely, substantial evidence of heterogeneity was common, with most meta-analyses of observational associations presenting I^2^ estimates exceeding 50%. Heterogeneity was also the most constraining criterion. When removed from the list of criteria to reach a convincing level of evidence, the number of associations increased substantially. Heterogeneity in meta-analyses of observational studies may be due to bias but also genuine difference between studies [5]. It is often hard to detangle between the two.

It is important to acknowledge the limits of our proposed criteria and of the ways that they can be combined to reach an overall grading. *P*-value thresholds are set arbitrarily, the random effects meta-analysis may produce inconsistent results [84], the excess significance of bias has limited power if only a few studies are statistically significant [21, 22], and similarly both small-study effect and excess significance testing may be misleading when there is substantial heterogeneity [85]. Even if all 7 criteria are fulfilled, observational evidence could still remain at risk of unmeasured confounding, undetected bias, and reverse causality [6]. One illustration would be the downgrading of the evidence for associations for which we re-analyzed the data using only cohort studies.

Furthermore, we should acknowledge that different types of observational associations vary a lot in prior plausibility and thus the amount of statistical support that is required to make them convincing is likely to vary. Fields like pharmacoepidemiology might be very reluctant to adopt a *P*-value threshold of *P* < 10^−6^ for signal detection of medication harms. In agnostic searches, conversely, even such *P*-value thresholds may not be low enough [86]. Field-specific setting of *P*-value thresholds has been proposed, e.g. through empirical calibration [87, 88], but such calibrations are still unspecified and lack consensus for the vast majority of fields in epidemiology.

Most decision-makers have required evidence of causality for interventions, but licensing based on observational evidence alone is becoming increasingly common [89, 90]. While discordant results between RCTs and observational studies were highlighted long ago [1, 91, 92], there is ongoing debate on whether overall there are big differences and even on whether these designs can be formally compared when the same factor/intervention is involved [93, 94]. Most of the evidence that has been systematically assessed to-date pertains to situations where therapeutic interventions are assessed [2]. On average, the two designs may give similar results [2], but single comparisons may deviate substantially in the effect size estimates and in some settings even average effects seem to differ markedly across designs [95]. The observational literature that we assessed was mostly compiled to assess risk factors rather than interventions per se. Most of these risk or protective factors would not be possible to operationalize into intervention equivalents. However, when both observational and randomized evidence were available, in our overview, point estimates in different direction were quite common, 37.1% for observational studies versus RCTs and 30.8% for observational versus MR studies. Discrepancies beyond chance in the effect size estimates occurred in 43.5% for observational studies versus RCTs and 54.7% for observational studies versus MR studies.

Our study has several limitations. First, the seven standardized criteria were pre-specified based on what had been done previously in umbrella reviews. However, no consensus exists for a gold standard against which any criteria may be affirmed to truly quantify strength of the evidence and risk of bias [36] in observational studies of risk factors. Other efforts to-date have focused mostly on interventional evidence from RCTs where some observational evidence may be included (e.g., GRADE [96]) or specifically for interventional observational studies (e.g. ROBIS [97]).

Second, even though we included tens of thousands of observational studies, our assessment covers only specific fields for which umbrella reviews had been performed and these may not necessarily be fully generalizable to all observational epidemiology. Furthermore, only 16 out of 57 umbrella reviews also investigated meta-analyses of RCTs and MR studies in addition to meta-analyses of observational studies. Thus, we might not be capturing all meta-analyses of RCTs and all MR studies that reflect our included associations. MR studies are fairly recent and may even be more difficult to capture as they are often included in large genome-wide associations without being clearly identified. Moreover, both false positives and false-negative claims of causality may be made with MR studies, e.g., in the presence of weak genetic instruments.

Third, we used existing umbrella reviews which themselves focus on already existing meta-analyses. We did not appraise ourselves the quality of the included meta-analyses as this was already performed by the umbrella reviews’ authors but flawed meta-analyses are not uncommon [98] and results should be taken with caution. The original studies may also be affected by selection bias, missing data, inadequate follow-up, and poor study conduct. For example, the serum uric acid [6] and statins [36] umbrella reviews also assessed the original studies in depth and found errors (e.g., incorrect data combining different level of exposure, use of duplicated data, and inclusion of different populations) that led to downgraded associations. Such errors require in-depth re-evaluation of the primary studies and their data. If anything, the proportion of associations with convincing or highly suggestive evidence might decrease even further, if one were to downgrade evidence because of the poor quality of meta-analyses and of primary studies. Finally, some of the included meta-analyses in umbrellas of different topics may have had some overlap, but we kept them so as to have each topic represented in its totality. We estimate that approximately 5% of the meta-analyses may be duplicates across two different topics, but the exact number depends on how exactly duplication/overlap is defined. Regardless, the proportion is low to affect the results materially.

## Conclusion

Allowing for these caveats, overall, our bird’s eye view evaluation across 3744 meta-analyses of observational evidence on risk factors suggests that strong, large-scale, consistent, and uncontested observational evidence is probably very uncommon, even though statistically significant results are very common. It is also uncommon to find consistent corroborating evidence from RCTs or MR studies. Associations from meta-analyses of observational studies can offer interesting leads but require great caution, especially when high validity is required for decision-making.

## Supplementary Information

**Additional file 1. Appendix Method 1:** Criteria for evaluating evidence strength.

**Additional file 2: Figures 1 to 5.** Forest plots of the proportions of associations for each level of evidence.

**Additional file 3: Figures 6 to 7.** Missing data.

**Additional file 4: Figure 8 to 15.** Forest plots of the proportions of associations fulfilling each criteria.

**Additional file 5: Table 1.** Kappa matrix across credibility criteria (below) and meta-analyzed across umbrella reviews (above).

**Additional file 6: Figures 16 to 43.** Forest plots of the meta-analyses of kappa estimated for each individual association.

**Additional file 7: Table 2.** Comparisons of observational studies and RCTs.

**Additional file 8: Table 3.** Comparisons of observational studies and MR studies.

**Additional file 9: Figures 44 to 45.** Forest plots of the ROR comparing meta-analyses of observational studies with meta-analyses of RCTs and with MR studies.

## Data Availability

The data used in this analysis are available to share.

## Abbreviations

AMSTAR: A MeaSurement Tool to Assess systematic Reviews
HR: Hazard ratio
MR: Mendelian randomization
OR: Odds ratio
P: *P*-value
RCT: Randomized controlled trials
RR: Relative risks
