# Supplementary material for: Validity of observational evidence on putative risk and protective factors: appraisal of 3744 meta-analyses on 57 topics
Source: BMC Med. 2021 Jul 6;19:157. doi: 10.1186/s12916-021-02020-6 (PMC8259334; doi:10.1186/s12916-021-02020-6)
Supplement: Supplementary file 1 — Additional file 1. Appendix Method 1: Criteria for evaluating evidence strength. [file 12916_2021_2020_MOESM1_ESM.pdf]

Appendix Method 1: Criteria for evaluating evidence strength

Summary effect and heterogeneity

The summary effect with its 95% confidence interval and heterogeneity may have been extracted from each original meta-analysis or re-calculated in a standard fashion by the umbrella review's authors using the study-level data. We preferred the latter option, whenever available. Authors may also choose to convert standardized mean metrics for continuous outcomes to odds ratios[26]. We consistently used random effects estimates for our assessments of the statistical significance criterion.

For heterogeneity between studies, the I^2 measure of inconsistency ranges between 0% and 100% and is the ratio of between-study variance over the sum of the within- and between-study variance[12]. Values exceeding 50% are usually considered to represent large heterogeneity.

Prediction intervals

Prediction intervals have been recommended as the best way to describe uncertainty and to further account for between-study heterogeneity[16,17]. They predict the range of effect size that would be expected in a new individual study. We extracted the 95% prediction intervals calculated by umbrella authors or calculate them, if they were not already available, using the random-effects summary effect size, its corresponding 95% confidence interval, the number of studies and a statistic of heterogeneity (I^2, Q statistic or tau-square). The prediction interval can only be calculated when the meta-analysis contains a minimum of three studies[17].

Asymmetry Tests for Small-Study Effects

Asymmetry, also known as small study effect, corresponds to whether smaller study tend to give a substantially larger estimates of effect size compared with larger studies. It may be due to publication and other selective reporting biases, but may also reflect true heterogeneity or occur by chance[18,19]. The regression asymmetry test proposed by Egger[10] examines whether the association between the estimated effect size and a measure of study size (e.g. the standard error or the variance is usually used) is greater than might be expected to occur by chance[18]. Indication of small study effects is claimed when $P \leq 0.10$. If not available for extraction and only if we had access to extracted study-level data then we performed the test ourselves.

Excess Significance Test

The excess significance test is an exploratory test used to evaluate whether an excess of studies with statistically significant findings ($P<0.05$) exist in the literature[22,23]. It assesses whether the observed (O) number of statistically significant studies differs from the expected (E) number using the χ^2 test: $A=[(O-E)^2/E+(O-E)^2/(n-E)]$ (significance threshold $P<0.10$). Alternatively, a binomial probability test may be used with similar inferences[23]. The expected number of statistically significant studies is estimated for each meta-analysis based on the sum of power estimated for each component study. The power of each component study is calculated with an algorithm that uses a non-central *t* distribution, by assuming the true effect size to be the same as that of the largest component study (with smallest variance) in the meta-analysis[21]. If not available for extraction and only if we had access to extracted study-level data then we performed the test ourselves.

Other Criteria

For the number of cases and the largest study component, we relied on the data extracted and presented by the authors of each umbrella review. When not available in the publication, we extracted the data ourselves. Of note, when the exposure contrast does not include the entire population, the sample size considered for the level of evidence classification is not the entire sample, but the sample included in the exposure contrast categories, e.g. if the contrast is between extreme quintiles, this would be the number of cases (or number of participants for continuous outcomes) in the two extreme quintiles. If prior umbrella reviews have mistakenly used the entire sample size in such cases, we corrected this error.
