# Supplementary material for: Validity of observational evidence on putative risk and protective factors: appraisal of 3744 meta-analyses on 57 topics
Source: BMC Med. 2021 Jul 6;19:157. doi: 10.1186/s12916-021-02020-6 (PMC8259334; doi:10.1186/s12916-021-02020-6)
Supplement: Supplementary file 2 — Additional file 2: Figures 1 to 5. Forest plots of the proportions of associations for each level of evidence. [file 12916_2021_2020_MOESM2_ESM.pdf]

Additional file 2: Figures 1 to Forest plots of the proportions of associations for each level of evidence

1) Convincing evidence

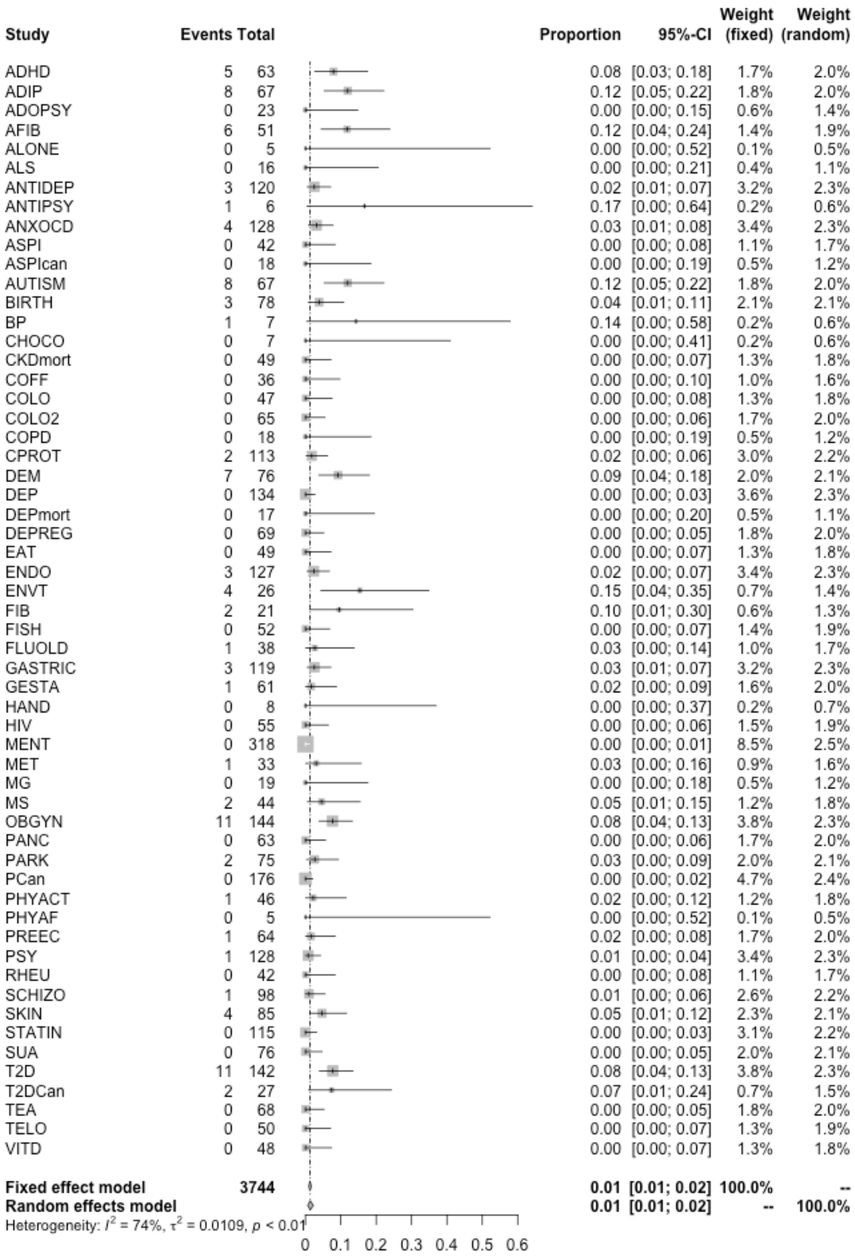

2) Highly suggestive evidence

Study	Events	Total	Proportion	95%-CI	Weight (fixed)	Weight (random)
ADHD	3	63	0.05	[0.01; 0.13]	1.7%	1.9%
ADIP	14	67	0.21	[0.12; 0.33]	1.8%	1.9%
ADOPSY	0	23	0.00	[0.00; 0.15]	0.6%	1.5%
AFIB	11	51	0.22	[0.11; 0.35]	1.4%	1.9%
ALONE	0	5	0.00	[0.00; 0.52]	0.1%	0.7%
ALS	0	16	0.00	[0.00; 0.21]	0.4%	1.3%
ANTIDEP	8	120	0.07	[0.03; 0.13]	3.2%	2.1%
ANTIPSY	2	6	0.33	[0.04; 0.78]	0.2%	0.8%
ANXOCD	2	128	0.02	[0.00; 0.06]	3.4%	2.1%
ASPI	0	42	0.00	[0.00; 0.08]	1.1%	1.8%
ASPIcan	0	18	0.00	[0.00; 0.19]	0.5%	1.4%
AUTISM	7	67	0.10	[0.04; 0.20]	1.8%	1.9%
BIRTH	8	78	0.10	[0.05; 0.19]	2.1%	2.0%
BP	1	7	0.14	[0.00; 0.58]	0.2%	0.9%
CHOCO	0	7	0.00	[0.00; 0.41]	0.2%	0.9%
CKDmort	0	49	0.00	[0.00; 0.07]	1.3%	1.9%
COFF	3	36	0.08	[0.02; 0.22]	1.0%	1.7%
COLO	0	47	0.00	[0.00; 0.08]	1.3%	1.8%
COLO2	0	65	0.00	[0.00; 0.06]	1.7%	1.9%
COPD	0	18	0.00	[0.00; 0.19]	0.5%	1.4%
CPROT	12	113	0.11	[0.06; 0.18]	3.0%	2.1%
DEM	5	76	0.07	[0.02; 0.15]	2.0%	2.0%
DEP	0	134	0.00	[0.00; 0.03]	3.6%	2.1%
DEPmort	4	17	0.24	[0.07; 0.50]	0.5%	1.4%
DEPREG	5	69	0.07	[0.02; 0.16]	1.8%	2.0%
EAT	2	49	0.04	[0.00; 0.14]	1.3%	1.9%
ENDO	13	127	0.10	[0.06; 0.17]	3.4%	2.1%
ENVT	2	26	0.08	[0.01; 0.25]	0.7%	1.6%
FIB	1	21	0.05	[0.00; 0.24]	0.6%	1.5%
FISH	0	52	0.00	[0.00; 0.07]	1.4%	1.9%
FLUOLD	3	38	0.08	[0.02; 0.21]	1.0%	1.8%
GASTRIC	7	119	0.06	[0.02; 0.12]	3.2%	2.1%
GESTA	13	61	0.21	[0.12; 0.34]	1.6%	1.9%
HAND	1	8	0.12	[0.00; 0.53]	0.2%	1.0%
HIV	0	55	0.00	[0.00; 0.06]	1.5%	1.9%
MENT	0	318	0.00	[0.00; 0.01]	8.5%	2.2%
MET	3	33	0.09	[0.02; 0.24]	0.9%	1.7%
MG	0	19	0.00	[0.00; 0.18]	0.5%	1.4%
MS	2	44	0.05	[0.01; 0.15]	1.2%	1.8%
OBGYN	28	144	0.19	[0.13; 0.27]	3.8%	2.1%
PANC	2	63	0.03	[0.00; 0.11]	1.7%	1.9%
PARK	6	75	0.08	[0.03; 0.17]	2.0%	2.0%
PCan	2	176	0.01	[0.00; 0.04]	4.7%	2.1%
PHYACT	2	46	0.04	[0.01; 0.15]	1.2%	1.8%
PHYAF	0	5	0.00	[0.00; 0.52]	0.1%	0.7%
PREEC	11	64	0.17	[0.09; 0.29]	1.7%	1.9%
PSY	2	128	0.02	[0.00; 0.06]	3.4%	2.1%
RHEU	0	42	0.00	[0.00; 0.08]	1.1%	1.8%
SCHIZO	4	98	0.04	[0.01; 0.10]	2.6%	2.0%
SKIN	9	85	0.11	[0.05; 0.19]	2.3%	2.0%
STATIN	2	115	0.02	[0.00; 0.06]	3.1%	2.1%
SUA	7	76	0.09	[0.04; 0.18]	2.0%	2.0%
T2D	34	142	0.24	[0.17; 0.32]	3.8%	2.1%
T2DCan	4	27	0.15	[0.04; 0.34]	0.7%	1.6%
TEA	1	68	0.01	[0.00; 0.08]	1.8%	1.9%
TELO	1	50	0.02	[0.00; 0.11]	1.3%	1.9%
VITD	6	48	0.12	[0.05; 0.25]	1.3%	1.8%
**Fixed effect model**		**3744**	**0.04**	**[0.04; 0.05]**	**100.0%**	**--**
**Random effects model**			**0.05**	**[0.03; 0.07]**	**--**	**100.0%**

Heterogeneity: $I^2 = 86\%$, $\tau^2 = 0.0230$, $p < 0.01$

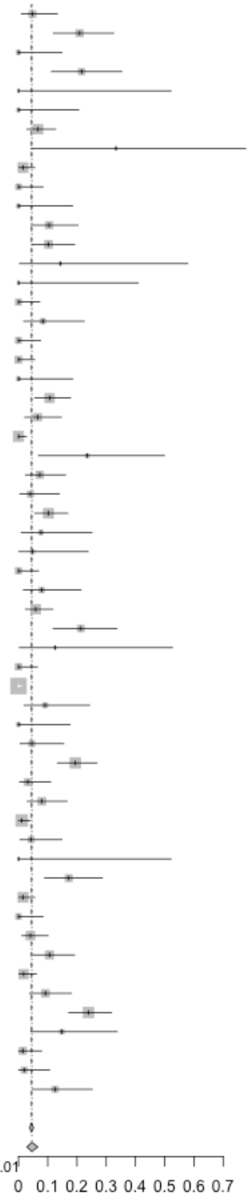

3) Suggestive evidence

Study	Events	Total	Proportion	95%-CI	Weight (fixed)	Weight (random)
ADHD	11	63	0.17	[0.09; 0.29]	1.7%	1.9%
ADIP	15	67	0.22	[0.13; 0.34]	1.8%	2.0%
ADOPSY	1	23	0.04	[0.00; 0.22]	0.6%	1.5%
AFIB	8	51	0.16	[0.07; 0.29]	1.4%	1.9%
ALONE	1	5	0.20	[0.01; 0.72]	0.1%	0.7%
ALS	3	16	0.19	[0.04; 0.46]	0.4%	1.3%
ANTIDEP	24	120	0.20	[0.13; 0.28]	3.2%	2.1%
ANTIPSY	3	6	0.50	[0.12; 0.88]	0.2%	0.8%
ANXOCD	3	128	0.02	[0.00; 0.07]	3.4%	2.1%
ASPI	0	42	0.00	[0.00; 0.08]	1.1%	1.8%
ASPIcan	0	18	0.00	[0.00; 0.19]	0.5%	1.4%
AUTISM	11	67	0.16	[0.08; 0.27]	1.8%	2.0%
BIRTH	10	78	0.13	[0.06; 0.22]	2.1%	2.0%
BP	2	7	0.29	[0.04; 0.71]	0.2%	0.8%
CHOCO	0	7	0.00	[0.00; 0.41]	0.2%	0.8%
CKDmort	22	49	0.45	[0.31; 0.60]	1.3%	1.9%
COFF	7	36	0.19	[0.08; 0.36]	1.0%	1.7%
COLO	4	47	0.09	[0.02; 0.20]	1.3%	1.8%
COLO2	4	65	0.06	[0.02; 0.15]	1.7%	1.9%
COPD	8	18	0.44	[0.22; 0.69]	0.5%	1.4%
CPROT	14	113	0.12	[0.07; 0.20]	3.0%	2.1%
DEM	10	76	0.13	[0.06; 0.23]	2.0%	2.0%
DEP	41	134	0.31	[0.23; 0.39]	3.6%	2.1%
DEPmort	2	17	0.12	[0.01; 0.36]	0.5%	1.3%
DEPREG	11	69	0.16	[0.08; 0.27]	1.8%	2.0%
EAT	4	49	0.08	[0.02; 0.20]	1.3%	1.9%
ENDO	14	127	0.11	[0.06; 0.18]	3.4%	2.1%
ENVT	1	26	0.04	[0.00; 0.20]	0.7%	1.6%
FIB	9	21	0.43	[0.22; 0.66]	0.6%	1.5%
FISH	2	52	0.04	[0.00; 0.13]	1.4%	1.9%
FLUOLD	6	38	0.16	[0.06; 0.31]	1.0%	1.7%
GASTRIC	3	119	0.03	[0.01; 0.07]	3.2%	2.1%
GESTA	9	61	0.15	[0.07; 0.26]	1.6%	1.9%
HAND	1	8	0.12	[0.00; 0.53]	0.2%	0.9%
HIV	9	55	0.16	[0.08; 0.29]	1.5%	1.9%
MENT	3	318	0.01	[0.00; 0.03]	8.5%	2.2%
MET	5	33	0.15	[0.05; 0.32]	0.9%	1.7%
MG	2	19	0.11	[0.01; 0.33]	0.5%	1.4%
MS	2	44	0.05	[0.01; 0.15]	1.2%	1.8%
OBGYN	23	144	0.16	[0.10; 0.23]	3.8%	2.1%
PANC	1	63	0.02	[0.00; 0.09]	1.7%	1.9%
PARK	9	75	0.12	[0.06; 0.22]	2.0%	2.0%
PCan	7	176	0.04	[0.02; 0.08]	4.7%	2.2%
PHYACT	5	46	0.11	[0.04; 0.24]	1.2%	1.8%
PHYAF	0	5	0.00	[0.00; 0.52]	0.1%	0.7%
PREEC	5	64	0.08	[0.03; 0.17]	1.7%	1.9%
PSY	11	128	0.09	[0.04; 0.15]	3.4%	2.1%
RHEU	7	42	0.17	[0.07; 0.31]	1.1%	1.8%
SCHIZO	5	98	0.05	[0.02; 0.12]	2.6%	2.1%
SKIN	11	85	0.13	[0.07; 0.22]	2.3%	2.0%
STATIN	21	115	0.18	[0.12; 0.27]	3.1%	2.1%
SUA	9	76	0.12	[0.06; 0.21]	2.0%	2.0%
T2D	28	142	0.20	[0.14; 0.27]	3.8%	2.1%
T2DCan	4	27	0.15	[0.04; 0.34]	0.7%	1.6%
TEA	2	68	0.03	[0.00; 0.10]	1.8%	2.0%
TELO	0	50	0.00	[0.00; 0.07]	1.3%	1.9%
VITD	7	48	0.15	[0.06; 0.28]	1.3%	1.8%
**Fixed effect model**		**3744**	**0.10**	**[0.09; 0.11]**	**100.0%**	**--**
**Random effects model**			**0.11**	**[0.09; 0.14]**	**--**	**100.0%**

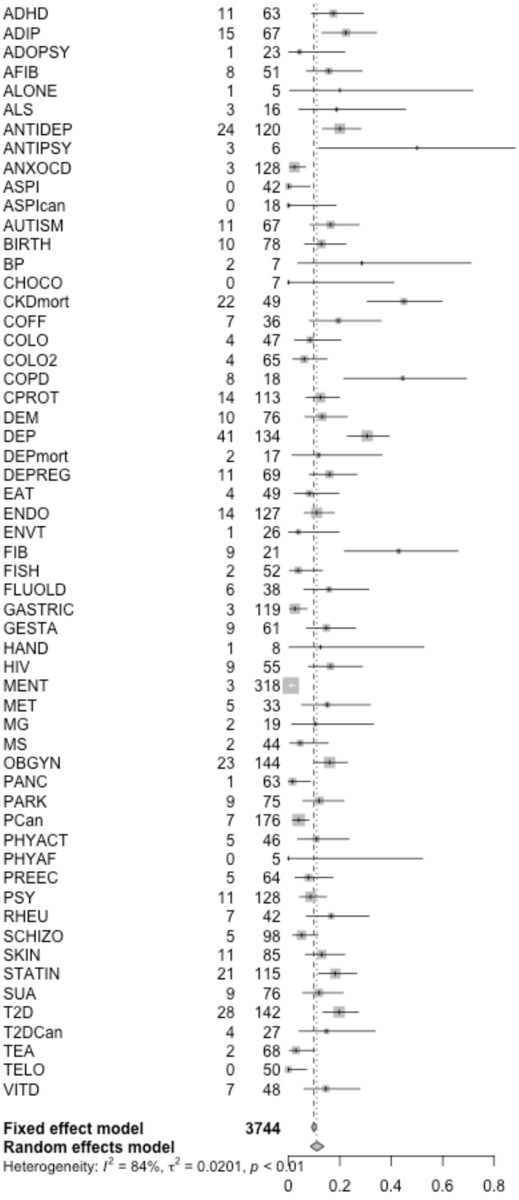

Heterogeneity: $I^2 = 84\%$, $\tau^2 = 0.0201$, $p < 0.01$

4) Weak evidence

Study	Events	Total	Proportion	95%-CI	Weight (fixed)	Weight (random)
ADHD	26	63	0.41	[0.29; 0.54]	1.7%	1.9%
ADIP	11	67	0.16	[0.08; 0.27]	1.8%	1.9%
ADOPSY	8	23	0.35	[0.16; 0.57]	0.6%	1.5%
AFIB	10	51	0.20	[0.10; 0.33]	1.4%	1.9%
ALONE	4	5	0.80	[0.28; 0.99]	0.1%	0.7%
ALS	6	16	0.38	[0.15; 0.65]	0.4%	1.4%
ANTIDEP	38	120	0.32	[0.23; 0.41]	3.2%	2.1%
ANTIPSY	0	6	0.00	[0.00; 0.46]	0.2%	0.8%
ANXOCD	60	128	0.47	[0.38; 0.56]	3.4%	2.1%
ASPI	11	42	0.26	[0.14; 0.42]	1.1%	1.8%
ASPIcan	12	18	0.67	[0.41; 0.87]	0.5%	1.4%
AUTISM	26	67	0.39	[0.27; 0.51]	1.8%	1.9%
BIRTH	29	78	0.37	[0.26; 0.49]	2.1%	2.0%
BP	2	7	0.29	[0.04; 0.71]	0.2%	0.9%
CHOCO	4	7	0.57	[0.18; 0.90]	0.2%	0.9%
CKDmort	11	49	0.22	[0.12; 0.37]	1.3%	1.9%
COFF	7	36	0.19	[0.08; 0.36]	1.0%	1.7%
COLO	27	47	0.57	[0.42; 0.72]	1.3%	1.8%
COLO2	25	65	0.38	[0.27; 0.51]	1.7%	1.9%
COPD	5	18	0.28	[0.10; 0.53]	0.5%	1.4%
CPROT	67	113	0.59	[0.50; 0.68]	3.0%	2.1%
DEM	33	76	0.43	[0.32; 0.55]	2.0%	2.0%
DEP	57	134	0.43	[0.34; 0.51]	3.6%	2.1%
DEPmort	11	17	0.65	[0.38; 0.86]	0.5%	1.4%
DEPREG	18	69	0.26	[0.16; 0.38]	1.8%	1.9%
EAT	35	49	0.71	[0.57; 0.83]	1.3%	1.9%
ENDO	26	127	0.20	[0.14; 0.29]	3.4%	2.1%
ENVT	15	26	0.58	[0.37; 0.77]	0.7%	1.6%
FIB	6	21	0.29	[0.11; 0.52]	0.6%	1.5%
FISH	10	52	0.19	[0.10; 0.33]	1.4%	1.9%
FLUOLD	15	38	0.39	[0.24; 0.57]	1.0%	1.8%
GASTRIC	82	119	0.69	[0.60; 0.77]	3.2%	2.1%
GESTA	28	61	0.46	[0.33; 0.59]	1.6%	1.9%
HAND	4	8	0.50	[0.16; 0.84]	0.2%	1.0%
HIV	30	55	0.55	[0.41; 0.68]	1.5%	1.9%
MENT	175	318	0.55	[0.49; 0.61]	8.5%	2.2%
MET	14	33	0.42	[0.25; 0.61]	0.9%	1.7%
MG	7	19	0.37	[0.16; 0.62]	0.5%	1.4%
MS	17	44	0.39	[0.24; 0.55]	1.2%	1.8%
OBGYN	42	144	0.29	[0.22; 0.37]	3.8%	2.1%
PANC	41	63	0.65	[0.52; 0.77]	1.7%	1.9%
PARK	18	75	0.24	[0.15; 0.35]	2.0%	2.0%
PCan	25	176	0.14	[0.09; 0.20]	4.7%	2.1%
PHYACT	5	46	0.11	[0.04; 0.24]	1.2%	1.8%
PHYAF	3	5	0.60	[0.15; 0.95]	0.1%	0.7%
PREEC	22	64	0.34	[0.23; 0.47]	1.7%	1.9%
PSY	64	128	0.50	[0.41; 0.59]	3.4%	2.1%
RHEU	26	42	0.62	[0.46; 0.76]	1.1%	1.8%
SCHIZO	52	98	0.53	[0.43; 0.63]	2.6%	2.0%
SKIN	34	85	0.40	[0.30; 0.51]	2.3%	2.0%
STATIN	42	115	0.37	[0.28; 0.46]	3.1%	2.1%
SUA	41	76	0.54	[0.42; 0.65]	2.0%	2.0%
T2D	43	142	0.30	[0.23; 0.39]	3.8%	2.1%
T2DCan	10	27	0.37	[0.19; 0.58]	0.7%	1.6%
TEA	18	68	0.26	[0.17; 0.39]	1.8%	1.9%
TELO	23	50	0.46	[0.32; 0.61]	1.3%	1.9%
VITD	16	48	0.33	[0.20; 0.48]	1.3%	1.8%
**Fixed effect model**		**3744**	**0.39**	**[0.38; 0.41]**	**100.0%**	**--**
**Random effects model**			**0.39**	**[0.35; 0.44]**	**--**	**100.0%**

Heterogeneity: $I^2 = 86\%$, $\tau^2 = 0.0241$, $p < 0.01$

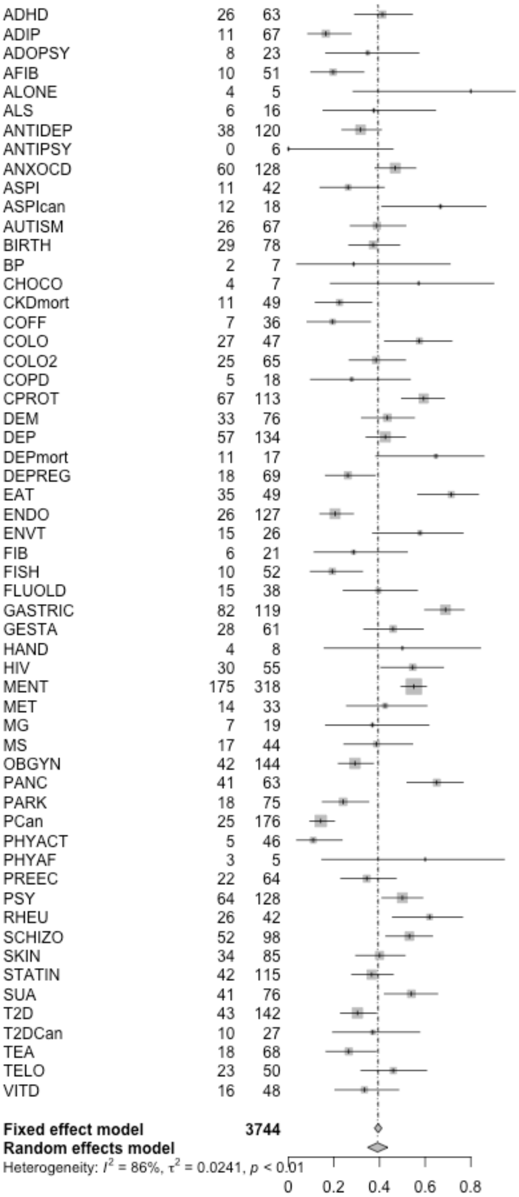

5) Non-significant associations

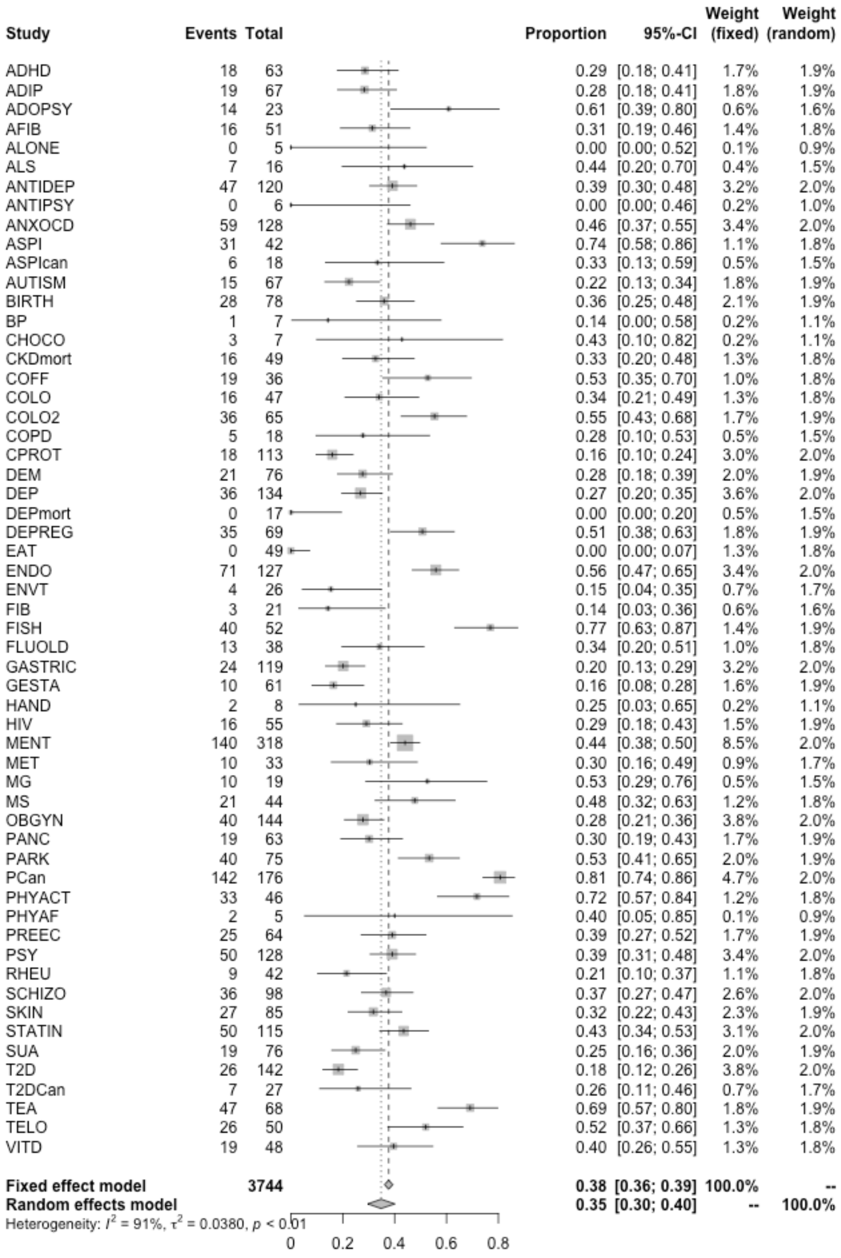

ADHD: Risk factor for attention deficit hyperactivity disorder ; ADIP: Adiposity and cancer outcomes; ADOPSY: Risk and protective factors for mental disorders with onset in childhood/adolescence; AFIB: Environmental factors and serum biomarkers for atrial fibrillation; ALONE: Factors associated to loneliness; ALS: Risk factor for amyotrophic lateral sclerosis; ANTIDEP: Antidepressant and adverse events; ANTIPSY: Antipsychotics and life-threatening events; ANXOCD: Risk and protective factors for anxiety and obsessive compulsive disorders; ASPI: Low-dose aspirin and health outcomes; ASPIcan: Aspirin and cancer outcomes; AUTISM: Environmental risk factors and biomarkers for autism spectrum disorder; BIRTH: Birth weight and later life events; BP: Environmental risk factors for bipolar disorder; CHOCO: Chocolate and health outcomes; CKDmort: Chronic kidney disease and mortality; COFF: Coffee and cancer risk; COLO: Risk factors for colorectal cancer metastasis and recurrence; COLO2: Non-genetic biomarkers and colorectal cancer risk; COPD: Risk factors for chronic obstructive pulmonary disease; CPROT: C-reactive protein and health outcomes; DEM: Environmental risk factors for dementia; DEP: Risk factors for depression; DEPmort: Depression and mortality; DEPPREG: Antidepressants during pregnancy and neonatal outcomes; EAT: Risk factors for eating disorders; ENDO: Risk factors for endometrial cancer; ENVT: Environmental risk factors for obesity; FIB: Dietary Fiber and health

outcomes; FISH: Fish and _-3 Fatty Acids consumptions and cancer outcomes; FLUOLD: Influenza vaccine in elderly and health outcomes; GASTRIC: Prognostic biomarkers for gastric cancer; GESTA: Risk factors for gestational diabetes; HAND: Handgrip strength and health outcomes; HIV: Human immunodeficiency virus infections and health outcomes; MENT: Peripheral biomarkers and major mental disorders; MET: Metformin and cancer outcomes; MG: Magnesium and health outcomes; MS: Environmental risk factors for multiple sclerosis; OBGYN: Obesity and gynecology/obstetric outcomes; PANC: Prognostic biomarkers for pancreatic ductal adenocarcinoma; PARK: Environmental risk factors and Parkinson's; PCan: Risk and protective factors for prostate cancer; PHYACT: Physical activity and cancer outcomes; PHYAF: Physical activity and atrial fibrillation outcomes; PREEC: Non-genetic risk factors for pre-eclampsia; PSY: Risk and protective factors for psychosis; RHEU: Environmental risk factors for rheumatic diseases; SCHIZO: Risk factors and peripheral biomarkers for schizophrenia spectrum disorders; SKIN: Non-genetic risk factors for skin cancer; STATIN: Statins and multiple non-cardiovascular outcomes; SUA: Serum uric acid and health outcomes; T2D: Risk factors for type 2 diabetes mellitus; T2DCan: Type 2 diabetes mellitus and cancer; TEA: Tea consumption and cancer; TELO: Telomere length and health outcomes; VITD: Vitamin D and health outcome
