# Supplementary material for: Validity of observational evidence on putative risk and protective factors: appraisal of 3744 meta-analyses on 57 topics
Source: BMC Med. 2021 Jul 6;19:157. doi: 10.1186/s12916-021-02020-6 (PMC8259334; doi:10.1186/s12916-021-02020-6)

Additional file 3: Figures 6 to 7 Missing data

6) Level of evidence of associations without missing data – all associations

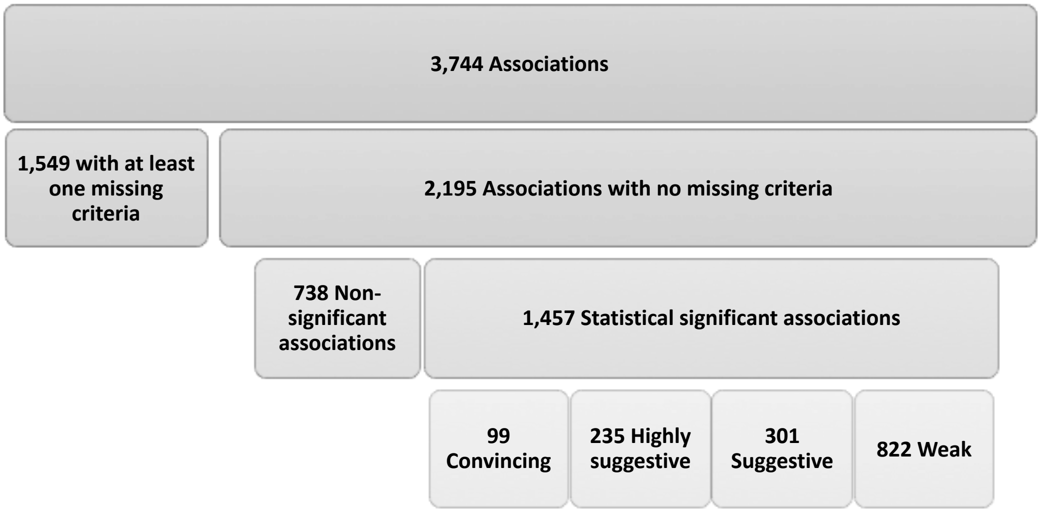

7) Distribution of associations fulfilling the different criteria – statistically significant associations

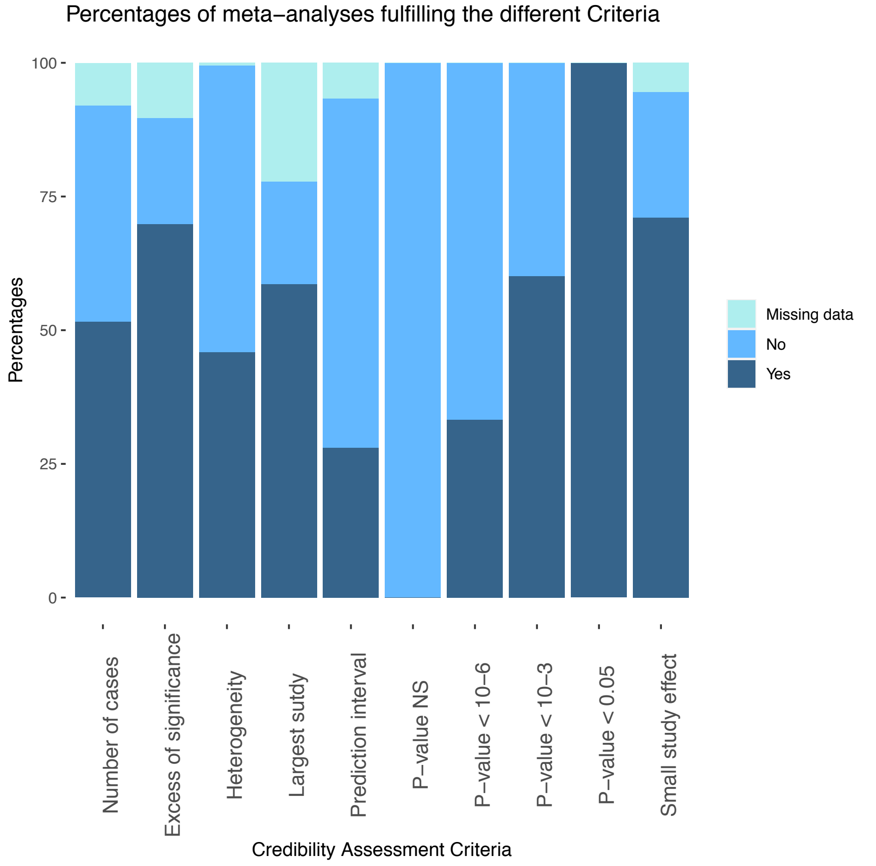

Supplement: Supplementary file 3 — Additional file 3: Figures 6 to 7. Missing data. [file 12916_2021_2020_MOESM3_ESM.pdf]
