# Supplementary material for: Validity of observational evidence on putative risk and protective factors: appraisal of 3744 meta-analyses on 57 topics
Source: BMC Med. 2021 Jul 6;19:157. doi: 10.1186/s12916-021-02020-6 (PMC8259334; doi:10.1186/s12916-021-02020-6)
Supplement: Supplementary file 4 — Additional file 4: Figure 8 to 15. Forest plots of the proportions of associations fulfilling each criteria. [file 12916_2021_2020_MOESM4_ESM.pdf]

Additional file 4: Figures 8 to Forest plots of the proportions of associations fulfilling each criteria

8) Associations with a statistical significance of P<10-6

Study	Events	Total	Proportion	95%-CI	Weight (fixed)	Weight (random)
ADHD	14	45	0.31	[0.18; 0.47]	2.0%	2.1%
ADIP	26	48	0.54	[0.39; 0.69]	2.1%	2.1%
ADOPSY	3	9	0.33	[0.07; 0.70]	0.4%	1.1%
AFIB	18	35	0.51	[0.34; 0.69]	1.5%	1.9%
ALONE	2	5	0.40	[0.05; 0.85]	0.2%	0.7%
ALS	0	9	0.00	[0.00; 0.34]	0.4%	1.1%
ANTIDEP	16	73	0.22	[0.13; 0.33]	3.2%	2.3%
ANTIPSY	3	6	0.50	[0.12; 0.88]	0.3%	0.8%
ANXOCD	20	69	0.29	[0.19; 0.41]	3.0%	2.2%
ASPI	1	11	0.09	[0.00; 0.41]	0.5%	1.2%
ASPIcan	2	12	0.17	[0.02; 0.48]	0.5%	1.3%
AUTISM	18	52	0.35	[0.22; 0.49]	2.3%	2.1%
BIRTH	19	50	0.38	[0.25; 0.53]	2.2%	2.1%
BP	2	6	0.33	[0.04; 0.78]	0.3%	0.8%
CHOCO	0	4	0.00	[0.00; 0.60]	0.2%	0.6%
CKDmort	15	33	0.45	[0.28; 0.64]	1.4%	1.9%
COFF	5	17	0.29	[0.10; 0.56]	0.7%	1.5%
COLO	14	31	0.45	[0.27; 0.64]	1.4%	1.9%
COLO2	3	29	0.10	[0.02; 0.27]	1.3%	1.8%
COPD	7	13	0.54	[0.25; 0.81]	0.6%	1.3%
CPROT	34	95	0.36	[0.26; 0.46]	4.2%	2.3%
DEM	15	55	0.27	[0.16; 0.41]	2.4%	2.2%
DEP	37	98	0.38	[0.28; 0.48]	4.3%	2.4%
DEPmort	7	17	0.41	[0.18; 0.67]	0.7%	1.5%
DEPREG	5	34	0.15	[0.05; 0.31]	1.5%	1.9%
EAT	16	41	0.39	[0.24; 0.55]	1.8%	2.0%
ENDO	22	56	0.39	[0.26; 0.53]	2.4%	2.2%
ENVT	10	22	0.45	[0.24; 0.68]	1.0%	1.7%
FIB	6	18	0.33	[0.13; 0.59]	0.8%	1.5%
FISH	0	12	0.00	[0.00; 0.26]	0.5%	1.3%
FLUOLD	6	25	0.24	[0.09; 0.45]	1.1%	1.7%
GASTRIC	40	95	0.42	[0.32; 0.53]	4.2%	2.3%
GESTA	14	51	0.27	[0.16; 0.42]	2.2%	2.1%
HAND	4	6	0.67	[0.22; 0.96]	0.3%	0.8%
HIV	11	39	0.28	[0.15; 0.45]	1.7%	2.0%
MENT	78	178	0.44	[0.36; 0.51]	7.8%	2.5%
MET	5	23	0.22	[0.07; 0.44]	1.0%	1.7%
MG	1	9	0.11	[0.00; 0.48]	0.4%	1.1%
MS	5	23	0.22	[0.07; 0.44]	1.0%	1.7%
OBGYN	50	104	0.48	[0.38; 0.58]	4.5%	2.4%
PANC	4	44	0.09	[0.03; 0.22]	1.9%	2.1%
PARK	11	35	0.31	[0.17; 0.49]	1.5%	1.9%
PCan	2	34	0.06	[0.01; 0.20]	1.5%	1.9%
PHYACT	4	13	0.31	[0.09; 0.61]	0.6%	1.3%
PHYAF	0	3	0.00	[0.00; 0.71]	0.1%	0.5%
PREEC	14	39	0.36	[0.21; 0.53]	1.7%	2.0%
PSY	33	78	0.42	[0.31; 0.54]	3.4%	2.3%
RHEU	13	33	0.39	[0.23; 0.58]	1.4%	1.9%
SCHIZO	18	62	0.29	[0.18; 0.42]	2.7%	2.2%
SKIN	21	58	0.36	[0.24; 0.50]	2.5%	2.2%
STATIN	4	65	0.06	[0.02; 0.15]	2.8%	2.2%
SUA	15	57	0.26	[0.16; 0.40]	2.5%	2.2%
T2D	46	116	0.40	[0.31; 0.49]	5.1%	2.4%
T2DCan	6	20	0.30	[0.12; 0.54]	0.9%	1.6%
TEA	1	21	0.05	[0.00; 0.24]	0.9%	1.6%
TELO	4	24	0.17	[0.05; 0.37]	1.0%	1.7%
VITD	12	29	0.41	[0.24; 0.61]	1.3%	1.8%
**Fixed effect model**		**2289**	**0.32**	**[0.30; 0.34]**	**100.0%**	**--**
**Random effects model**			**0.29**	**[0.25; 0.33]**	**--**	**100.0%**

Heterogeneity: $I^2 = 75\%$, $\tau^2 = 0.0187$, $p < 0.01$

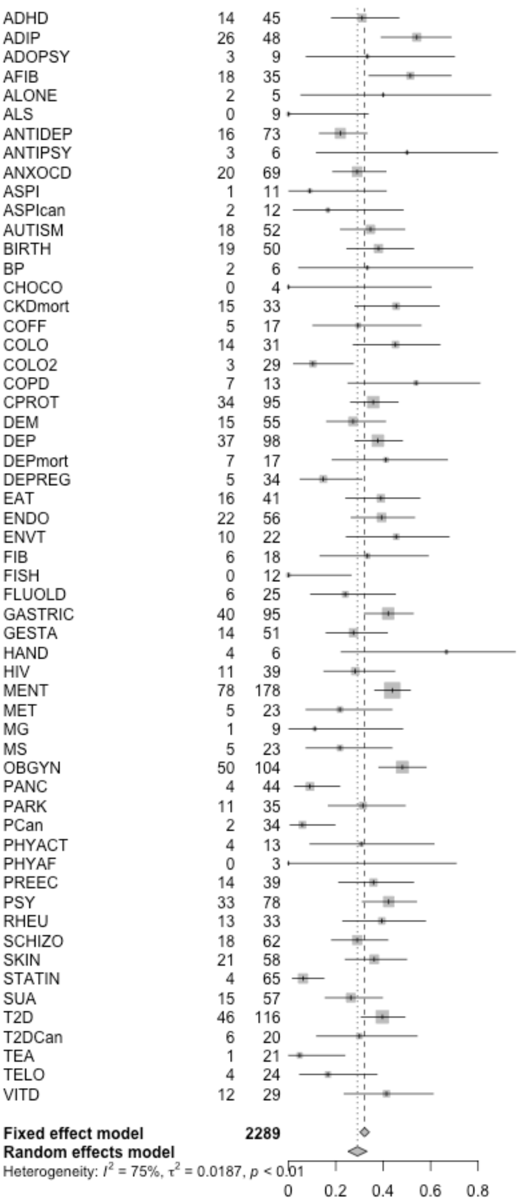

9) Associations with a statistical significance of P<0.001

Study	Events	Total	Proportion	95%-CI	Weight (fixed)	Weight (random)
ADHD	29	45	0.64	[0.49; 0.78]	2.0%	2.1%
ADIP	41	48	0.85	[0.72; 0.94]	2.1%	2.1%
ADOPSY	6	9	0.67	[0.30; 0.93]	0.4%	1.1%
AFIB	27	35	0.77	[0.60; 0.90]	1.5%	1.9%
ALONE	4	5	0.80	[0.28; 0.99]	0.2%	0.7%
ALS	5	9	0.56	[0.21; 0.86]	0.4%	1.1%
ANTIDEP	44	73	0.60	[0.48; 0.72]	3.2%	2.3%
ANTIPSY	6	6	1.00	[0.54; 1.00]	0.3%	0.8%
ANXOCD	41	69	0.59	[0.47; 0.71]	3.0%	2.3%
ASPI	2	11	0.18	[0.02; 0.52]	0.5%	1.2%
ASPIcan	4	12	0.33	[0.10; 0.65]	0.5%	1.2%
AUTISM	33	52	0.63	[0.49; 0.76]	2.3%	2.1%
BIRTH	31	50	0.62	[0.47; 0.75]	2.2%	2.1%
BP	4	6	0.67	[0.22; 0.96]	0.3%	0.8%
CHOCO	0	4	0.00	[0.00; 0.60]	0.2%	0.6%
CKDmort	22	33	0.67	[0.48; 0.82]	1.4%	1.9%
COFF	10	17	0.59	[0.33; 0.82]	0.7%	1.5%
COLO	18	31	0.58	[0.39; 0.75]	1.4%	1.9%
COLO2	7	29	0.24	[0.10; 0.44]	1.3%	1.8%
COPD	11	13	0.85	[0.55; 0.98]	0.6%	1.3%
CPROT	67	95	0.71	[0.60; 0.79]	4.2%	2.4%
DEM	30	55	0.55	[0.41; 0.68]	2.4%	2.2%
DEP	66	98	0.67	[0.57; 0.76]	4.3%	2.4%
DEPmort	10	17	0.59	[0.33; 0.82]	0.7%	1.5%
DEPREG	18	34	0.53	[0.35; 0.70]	1.5%	1.9%
EAT	27	41	0.66	[0.49; 0.80]	1.8%	2.0%
ENDO	35	56	0.62	[0.49; 0.75]	2.4%	2.2%
ENVT	15	22	0.68	[0.45; 0.86]	1.0%	1.7%
FIB	14	18	0.78	[0.52; 0.94]	0.8%	1.5%
FISH	3	12	0.25	[0.05; 0.57]	0.5%	1.2%
FLUOLD	15	25	0.60	[0.39; 0.79]	1.1%	1.7%
GASTRIC	68	95	0.72	[0.61; 0.80]	4.2%	2.4%
GESTA	29	51	0.57	[0.42; 0.71]	2.2%	2.1%
HAND	5	6	0.83	[0.36; 1.00]	0.3%	0.8%
HIV	30	39	0.77	[0.61; 0.89]	1.7%	2.0%
MENT	78	178	0.44	[0.36; 0.51]	7.8%	2.5%
MET	11	23	0.48	[0.27; 0.69]	1.0%	1.7%
MG	2	9	0.22	[0.03; 0.60]	0.4%	1.1%
MS	11	23	0.48	[0.27; 0.69]	1.0%	1.7%
OBGYN	78	104	0.75	[0.66; 0.83]	4.5%	2.4%
PANC	24	44	0.55	[0.39; 0.70]	1.9%	2.1%
PARK	21	35	0.60	[0.42; 0.76]	1.5%	1.9%
PCan	9	34	0.26	[0.13; 0.44]	1.5%	1.9%
PHYACT	8	13	0.62	[0.32; 0.86]	0.6%	1.3%
PHYAF	0	3	0.00	[0.00; 0.71]	0.1%	0.5%
PREEC	23	39	0.59	[0.42; 0.74]	1.7%	2.0%
PSY	47	78	0.60	[0.49; 0.71]	3.4%	2.3%
RHEU	21	33	0.64	[0.45; 0.80]	1.4%	1.9%
SCHIZO	37	62	0.60	[0.46; 0.72]	2.7%	2.2%
SKIN	35	58	0.60	[0.47; 0.73]	2.5%	2.2%
STATIN	35	65	0.54	[0.41; 0.66]	2.8%	2.2%
SUA	37	57	0.65	[0.51; 0.77]	2.5%	2.2%
T2D	78	116	0.67	[0.58; 0.76]	5.1%	2.4%
T2DCan	11	20	0.55	[0.32; 0.77]	0.9%	1.6%
TEA	3	21	0.14	[0.03; 0.36]	0.9%	1.6%
TELO	9	24	0.38	[0.19; 0.59]	1.0%	1.7%
VITD	22	29	0.76	[0.56; 0.90]	1.3%	1.8%
**Fixed effect model**		**2289**	**0.60**	**[0.58; 0.62]**	**100.0%**	**--**
**Random effects model**			**0.59**	**[0.54; 0.63]**	**--**	**100.0%**

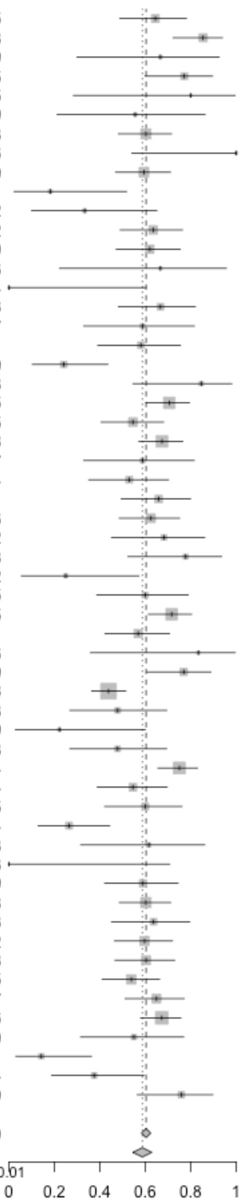

Heterogeneity: $I^2 = 73\%$, $\tau^2 = 0.0173$, $p < 0.01$

10) More than 1000 cases included (or more than 20 000 participants for continuous outcomes)

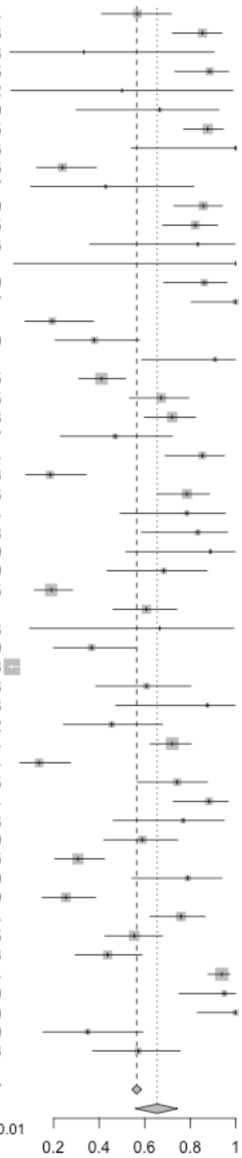

Study	Events	Total	Proportion	95%-CI	Weight (fixed)	Weight (random)
ADHD	25	44	0.57	[0.41; 0.72]	2.1%	1.9%
ADIP	41	48	0.85	[0.72; 0.94]	2.3%	2.0%
ADOPSY	1	3	0.33	[0.01; 0.91]	0.1%	1.2%
AFIB	31	35	0.89	[0.73; 0.97]	1.7%	1.9%
ALONE	1	2	0.50	[0.01; 0.99]	0.1%	1.0%
ALS	6	9	0.67	[0.30; 0.93]	0.4%	1.7%
ANTIDEP	57	65	0.88	[0.77; 0.95]	3.1%	2.0%
ANTIPSY	6	6	1.00	[0.54; 1.00]	0.3%	1.5%
ANXOCD	11	46	0.24	[0.13; 0.39]	2.2%	2.0%
ASPI	3	7	0.43	[0.10; 0.82]	0.3%	1.6%
AUTISM	42	49	0.86	[0.73; 0.94]	2.3%	2.0%
BIRTH	37	45	0.82	[0.68; 0.92]	2.1%	2.0%
BP	5	6	0.83	[0.36; 1.00]	0.3%	1.5%
CHOCO	1	1	1.00	[0.03; 1.00]	0.0%	0.7%
CKDmort	25	29	0.86	[0.68; 0.96]	1.4%	1.9%
COFF	17	17	1.00	[0.80; 1.00]	0.8%	1.8%
COLO	6	31	0.19	[0.07; 0.37]	1.5%	1.9%
COLO2	11	29	0.38	[0.21; 0.58]	1.4%	1.9%
COPD	10	11	0.91	[0.59; 1.00]	0.5%	1.7%
CPROT	39	95	0.41	[0.31; 0.52]	4.5%	2.0%
DEM	37	55	0.67	[0.53; 0.79]	2.6%	2.0%
DEP	49	68	0.72	[0.60; 0.82]	3.2%	2.0%
DEPmort	8	17	0.47	[0.23; 0.72]	0.8%	1.8%
DEPREG	29	34	0.85	[0.69; 0.95]	1.6%	1.9%
EAT	7	38	0.18	[0.08; 0.34]	1.8%	1.9%
ENDO	44	56	0.79	[0.66; 0.88]	2.7%	2.0%
ENVT	11	14	0.79	[0.49; 0.95]	0.7%	1.8%
FIB	15	18	0.83	[0.59; 0.96]	0.9%	1.8%
FISH	8	9	0.89	[0.52; 1.00]	0.4%	1.7%
FLUOLD	13	19	0.68	[0.43; 0.87]	0.9%	1.8%
GASTRIC	18	95	0.19	[0.12; 0.28]	4.5%	2.0%
GESTA	31	51	0.61	[0.46; 0.74]	2.4%	2.0%
HAND	2	3	0.67	[0.09; 0.99]	0.1%	1.2%
HIV	11	30	0.37	[0.20; 0.56]	1.4%	1.9%
MENT	3	178	0.02	[0.00; 0.05]	8.4%	2.0%
MET	14	23	0.61	[0.39; 0.80]	1.1%	1.9%
MG	7	8	0.88	[0.47; 1.00]	0.4%	1.6%
MS	10	22	0.45	[0.24; 0.68]	1.0%	1.9%
OBGYN	75	104	0.72	[0.62; 0.80]	4.9%	2.0%
PANC	6	44	0.14	[0.05; 0.27]	2.1%	1.9%
PARK	26	35	0.74	[0.57; 0.88]	1.7%	1.9%
PCan	30	34	0.88	[0.73; 0.97]	1.6%	1.9%
PHYACT	10	13	0.77	[0.46; 0.95]	0.6%	1.8%
PREEC	23	39	0.59	[0.42; 0.74]	1.9%	1.9%
PSY	23	75	0.31	[0.21; 0.42]	3.6%	2.0%
RHEU	15	19	0.79	[0.54; 0.94]	0.9%	1.8%
SCHIZO	15	59	0.25	[0.15; 0.38]	2.8%	2.0%
SKIN	41	54	0.76	[0.62; 0.87]	2.6%	2.0%
STATIN	36	65	0.55	[0.43; 0.68]	3.1%	2.0%
SUA	21	48	0.44	[0.29; 0.59]	2.3%	2.0%
T2D	107	114	0.94	[0.88; 0.97]	5.4%	2.0%
T2DCan	19	20	0.95	[0.75; 1.00]	0.9%	1.9%
TEA	20	20	1.00	[0.83; 1.00]	0.9%	1.9%
TELO	7	20	0.35	[0.15; 0.59]	0.9%	1.9%
VITD	16	28	0.57	[0.37; 0.76]	1.3%	1.9%
**Fixed effect model**		**2107**	**0.57**	**[0.54; 0.59]**	**100.0%**	**--**
**Random effects model**			**0.65**	**[0.56; 0.74]**	**--**	**100.0%**

Heterogeneity: $I^2 = 95\%$, $\tau^2 = 0.1237$, $p < 0.01$

11) The largest component study reporting a significant result P<0.05

Study	Events	Total	Proportion	95%-CI	Weight (fixed)	Weight (random)
ADHD	34	45	0.76	[0.60; 0.87]	2.5%	2.7%
ADIP	43	48	0.90	[0.77; 0.97]	2.7%	2.7%
AFIB	32	35	0.91	[0.77; 0.98]	2.0%	2.4%
ALONE	4	5	0.80	[0.28; 0.99]	0.3%	0.7%
ALS	7	9	0.78	[0.40; 0.97]	0.5%	1.1%
ANTIDEP	52	73	0.71	[0.59; 0.81]	4.1%	3.1%
ANTIPSY	5	6	0.83	[0.36; 1.00]	0.3%	0.8%
ANXOCD	58	69	0.84	[0.73; 0.92]	3.9%	3.0%
ASPIcan	5	12	0.42	[0.15; 0.72]	0.7%	1.4%
AUTISM	40	52	0.77	[0.63; 0.87]	2.9%	2.8%
BIRTH	33	50	0.66	[0.51; 0.79]	2.8%	2.8%
BP	5	5	1.00	[0.48; 1.00]	0.3%	0.7%
CHOCO	2	4	0.50	[0.07; 0.93]	0.2%	0.6%
COFF	11	17	0.65	[0.38; 0.86]	1.0%	1.7%
CPROT	75	95	0.79	[0.69; 0.87]	5.3%	3.2%
DEM	39	55	0.71	[0.57; 0.82]	3.1%	2.8%
DEPmort	12	17	0.71	[0.44; 0.90]	1.0%	1.7%
DEPREG	27	34	0.79	[0.62; 0.91]	1.9%	2.4%
EAT	28	41	0.68	[0.52; 0.82]	2.3%	2.6%
ENDO	44	56	0.79	[0.66; 0.88]	3.1%	2.9%
ENVT	19	22	0.86	[0.65; 0.97]	1.2%	2.0%
FIB	11	18	0.61	[0.36; 0.83]	1.0%	1.8%
FLUOLD	19	23	0.83	[0.61; 0.95]	1.3%	2.0%
GASTRIC	71	95	0.75	[0.65; 0.83]	5.3%	3.2%
GESTA	37	51	0.73	[0.58; 0.84]	2.9%	2.8%
HAND	6	6	1.00	[0.54; 1.00]	0.3%	0.8%
MET	14	23	0.61	[0.39; 0.80]	1.3%	2.0%
MS	18	23	0.78	[0.56; 0.93]	1.3%	2.0%
OBGYN	94	104	0.90	[0.83; 0.95]	5.8%	3.3%
PANC	33	44	0.75	[0.60; 0.87]	2.5%	2.6%
PARK	19	35	0.54	[0.37; 0.71]	2.0%	2.4%
PCan	22	34	0.65	[0.46; 0.80]	1.9%	2.4%
PHYACT	8	13	0.62	[0.32; 0.86]	0.7%	1.5%
PHYAF	1	2	0.50	[0.01; 0.99]	0.1%	0.3%
PREEC	21	37	0.57	[0.39; 0.73]	2.1%	2.5%
PSY	49	78	0.63	[0.51; 0.74]	4.4%	3.1%
SCHIZO	49	62	0.79	[0.67; 0.88]	3.5%	2.9%
SKIN	45	58	0.78	[0.65; 0.87]	3.3%	2.9%
STATIN	52	65	0.80	[0.68; 0.89]	3.6%	3.0%
SUA	45	53	0.85	[0.72; 0.93]	3.0%	2.8%
T2D	97	116	0.84	[0.76; 0.90]	6.5%	3.3%
T2DCan	17	20	0.85	[0.62; 0.97]	1.1%	1.9%
TEA	6	21	0.29	[0.11; 0.52]	1.2%	1.9%
TELO	14	22	0.64	[0.41; 0.83]	1.2%	2.0%
VITD	20	28	0.71	[0.51; 0.87]	1.6%	2.2%
**Fixed effect model**		**1781**	**0.76**	**[0.74; 0.78]**	**100.0%**	**--**
**Random effects model**			**0.75**	**[0.71; 0.78]**	**--**	**100.0%**

Heterogeneity: $I^2 = 64\%$, $\tau^2 = 0.0112$, $p < 0.01$

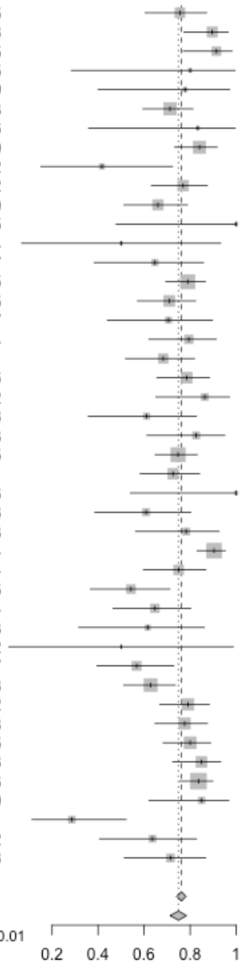

12) A 95% prediction interval that excluded the null

Study	Events	Total	Proportion	95%-CI	Weight (fixed)	Weight (random)
ADHD	16	43	0.37	[0.23; 0.53]	2.0%	2.1%
ADIP	19	43	0.44	[0.29; 0.60]	2.0%	2.1%
ADOPSY	3	8	0.38	[0.09; 0.76]	0.4%	1.0%
AFIB	12	35	0.34	[0.19; 0.52]	1.6%	2.0%
ALONE	3	5	0.60	[0.15; 0.95]	0.2%	0.7%
ALS	3	9	0.33	[0.07; 0.70]	0.4%	1.1%
ANTIDEP	24	69	0.35	[0.24; 0.47]	3.2%	2.4%
ANTIPSY	2	6	0.33	[0.04; 0.78]	0.3%	0.8%
ANXOCD	17	49	0.35	[0.22; 0.50]	2.3%	2.2%
ASPI	6	11	0.55	[0.23; 0.83]	0.5%	1.2%
ASPIcan	2	12	0.17	[0.02; 0.48]	0.6%	1.3%
AUTISM	19	47	0.40	[0.26; 0.56]	2.2%	2.2%
BIRTH	20	50	0.40	[0.26; 0.55]	2.3%	2.2%
BP	1	5	0.20	[0.01; 0.72]	0.2%	0.7%
CHOCO	1	4	0.25	[0.01; 0.81]	0.2%	0.6%
CKDmort	7	32	0.22	[0.09; 0.40]	1.5%	2.0%
COFF	5	17	0.29	[0.10; 0.56]	0.8%	1.5%
COLO	19	31	0.61	[0.42; 0.78]	1.5%	1.9%
COLO2	25	29	0.86	[0.68; 0.96]	1.4%	1.9%
COPD	3	13	0.23	[0.05; 0.54]	0.6%	1.3%
CPROT	24	95	0.25	[0.17; 0.35]	4.4%	2.5%
DEM	16	55	0.29	[0.18; 0.43]	2.6%	2.3%
DEP	25	98	0.26	[0.17; 0.35]	4.6%	2.5%
DEPmort	3	17	0.18	[0.04; 0.43]	0.8%	1.5%
DEPREG	9	34	0.26	[0.13; 0.44]	1.6%	2.0%
EAT	9	38	0.24	[0.11; 0.40]	1.8%	2.1%
ENDO	17	48	0.35	[0.22; 0.51]	2.2%	2.2%
ENVT	8	21	0.38	[0.18; 0.62]	1.0%	1.7%
FIB	5	18	0.28	[0.10; 0.53]	0.8%	1.6%
FISH	2	10	0.20	[0.03; 0.56]	0.5%	1.1%
FLUOLD	4	16	0.25	[0.07; 0.52]	0.7%	1.5%
GASTRIC	36	94	0.38	[0.28; 0.49]	4.4%	2.5%
GESTA	18	51	0.35	[0.22; 0.50]	2.4%	2.2%
HAND	2	6	0.33	[0.04; 0.78]	0.3%	0.8%
HIV	5	38	0.13	[0.04; 0.28]	1.8%	2.1%
MENT	16	178	0.09	[0.05; 0.14]	8.3%	2.7%
MET	2	22	0.09	[0.01; 0.29]	1.0%	1.7%
MG	1	9	0.11	[0.00; 0.48]	0.4%	1.1%
MS	10	20	0.50	[0.27; 0.73]	0.9%	1.6%
OBGYN	41	95	0.43	[0.33; 0.54]	4.4%	2.5%
PANC	8	34	0.24	[0.11; 0.41]	1.6%	2.0%
PARK	11	34	0.32	[0.17; 0.51]	1.6%	2.0%
PCan	11	31	0.35	[0.19; 0.55]	1.5%	1.9%
PHYACT	3	12	0.25	[0.05; 0.57]	0.6%	1.3%
PREEC	9	38	0.24	[0.11; 0.40]	1.8%	2.1%
PSY	27	71	0.38	[0.27; 0.50]	3.3%	2.4%
RHEU	3	33	0.09	[0.02; 0.24]	1.5%	2.0%
SCHIZO	15	61	0.25	[0.14; 0.37]	2.9%	2.3%
SKIN	17	58	0.29	[0.18; 0.43]	2.7%	2.3%
STATIN	13	59	0.22	[0.12; 0.35]	2.8%	2.3%
SUA	19	51	0.37	[0.24; 0.52]	2.4%	2.2%
T2D	34	116	0.29	[0.21; 0.38]	5.4%	2.6%
T2DCan	4	20	0.20	[0.06; 0.44]	0.9%	1.6%
TEA	3	21	0.14	[0.03; 0.36]	1.0%	1.7%
VITD	5	16	0.31	[0.11; 0.59]	0.7%	1.5%
**Fixed effect model**		**2136**	**0.29**	**[0.27; 0.31]**	**100.0%**	**--**
**Random effects model**			**0.30**	**[0.26; 0.34]**	**--**	**100.0%**

Heterogeneity: $I^2 = 71\%$, $\tau^2 = 0.0160$, $p < 0.01$

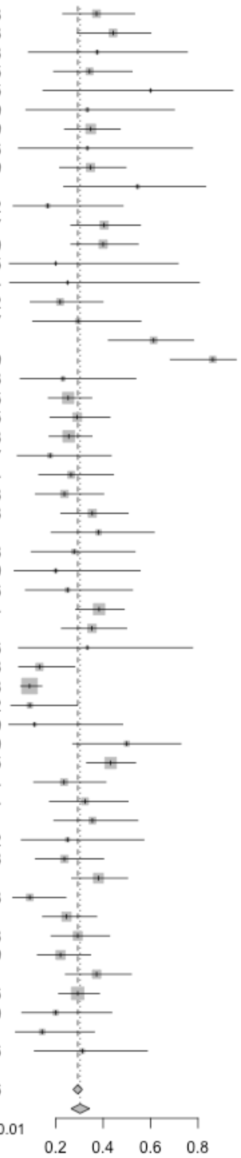

13) Absence of large heterogeneity I2<50%

Study	Events	Total	Proportion	95%-CI	Weight (fixed)	Weight (random)
ADHD	22	45	0.49	[0.34; 0.64]	2.0%	2.0%
ADIP	28	48	0.58	[0.43; 0.72]	2.1%	2.1%
ADOPSY	4	9	0.44	[0.14; 0.79]	0.4%	1.2%
AFIB	14	35	0.40	[0.24; 0.58]	1.5%	1.9%
ALONE	1	5	0.20	[0.01; 0.72]	0.2%	0.8%
ALS	7	9	0.78	[0.40; 0.97]	0.4%	1.2%
ANTIDEP	33	73	0.45	[0.34; 0.57]	3.2%	2.2%
ANTIPSY	1	6	0.17	[0.00; 0.64]	0.3%	0.9%
ANXOCD	44	69	0.64	[0.51; 0.75]	3.0%	2.2%
ASPI	5	11	0.45	[0.17; 0.77]	0.5%	1.3%
ASPIcan	5	12	0.42	[0.15; 0.72]	0.5%	1.3%
AUTISM	28	52	0.54	[0.39; 0.68]	2.3%	2.1%
BIRTH	30	50	0.60	[0.45; 0.74]	2.2%	2.1%
BP	3	6	0.50	[0.12; 0.88]	0.3%	0.9%
CHOCO	2	4	0.50	[0.07; 0.93]	0.2%	0.7%
CKDmort	9	33	0.27	[0.13; 0.46]	1.4%	1.9%
COFF	11	17	0.65	[0.38; 0.86]	0.7%	1.6%
COLO	21	31	0.68	[0.49; 0.83]	1.4%	1.9%
COLO2	13	29	0.45	[0.26; 0.64]	1.3%	1.8%
COPD	2	13	0.15	[0.02; 0.45]	0.6%	1.4%
CPROT	51	95	0.54	[0.43; 0.64]	4.2%	2.3%
DEM	31	55	0.56	[0.42; 0.70]	2.4%	2.1%
DEP	37	98	0.38	[0.28; 0.48]	4.3%	2.3%
DEPmort	6	17	0.35	[0.14; 0.62]	0.7%	1.6%
DEPREG	20	34	0.59	[0.41; 0.75]	1.5%	1.9%
EAT	18	41	0.44	[0.28; 0.60]	1.8%	2.0%
ENDO	36	56	0.64	[0.50; 0.77]	2.5%	2.1%
ENVT	8	22	0.36	[0.17; 0.59]	1.0%	1.7%
FIB	11	18	0.61	[0.36; 0.83]	0.8%	1.6%
FISH	9	12	0.75	[0.43; 0.95]	0.5%	1.3%
FLUOLD	11	25	0.44	[0.24; 0.65]	1.1%	1.8%
GASTRIC	54	95	0.57	[0.46; 0.67]	4.2%	2.3%
GESTA	26	51	0.51	[0.37; 0.65]	2.2%	2.1%
HAND	0	6	0.00	[0.00; 0.46]	0.3%	0.9%
HIV	14	39	0.36	[0.21; 0.53]	1.7%	2.0%
MENT	32	178	0.18	[0.13; 0.24]	7.8%	2.4%
MET	9	23	0.39	[0.20; 0.61]	1.0%	1.7%
MG	6	9	0.67	[0.30; 0.93]	0.4%	1.2%
MS	10	22	0.45	[0.24; 0.68]	1.0%	1.7%
OBGYN	53	104	0.51	[0.41; 0.61]	4.6%	2.3%
PANC	28	44	0.64	[0.48; 0.78]	1.9%	2.0%
PARK	19	35	0.54	[0.37; 0.71]	1.5%	1.9%
PCan	30	34	0.88	[0.73; 0.97]	1.5%	1.9%
PHYACT	8	13	0.62	[0.32; 0.86]	0.6%	1.4%
PHYAF	0	2	0.00	[0.00; 0.84]	0.1%	0.4%
PREEC	19	39	0.49	[0.32; 0.65]	1.7%	2.0%
PSY	51	78	0.65	[0.54; 0.76]	3.4%	2.2%
RHEU	5	33	0.15	[0.05; 0.32]	1.4%	1.9%
SCHIZO	15	62	0.24	[0.14; 0.37]	2.7%	2.1%
SKIN	22	58	0.38	[0.26; 0.52]	2.5%	2.1%
STATIN	32	65	0.49	[0.37; 0.62]	2.9%	2.2%
SUA	13	51	0.25	[0.14; 0.40]	2.2%	2.1%
T2D	43	116	0.37	[0.28; 0.47]	5.1%	2.3%
T2DCan	6	20	0.30	[0.12; 0.54]	0.9%	1.7%
TEA	15	21	0.71	[0.48; 0.89]	0.9%	1.7%
TELO	6	24	0.25	[0.10; 0.47]	1.1%	1.8%
VITD	13	25	0.52	[0.31; 0.72]	1.1%	1.8%
**Fixed effect model**		**2277**	**0.46**	**[0.44; 0.48]**	**100.0%**	**--**
**Random effects model**			**0.47**	**[0.42; 0.51]**	**--**	**100.0%**

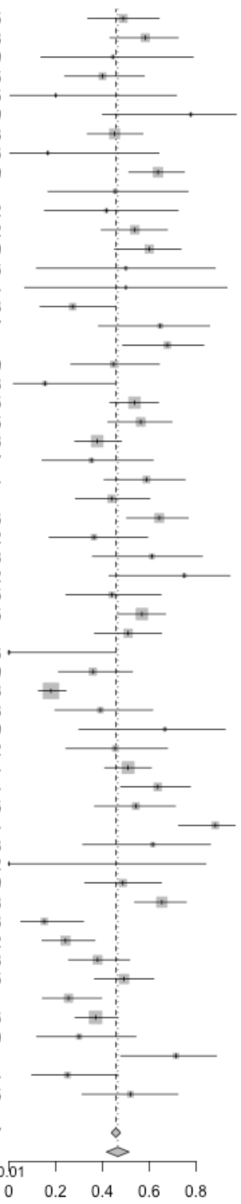

Heterogeneity: $I^2 = 80\%$, $\tau^2 = 0.0246$, $p < 0.01$

14) No evidence of small study effect P>0.10

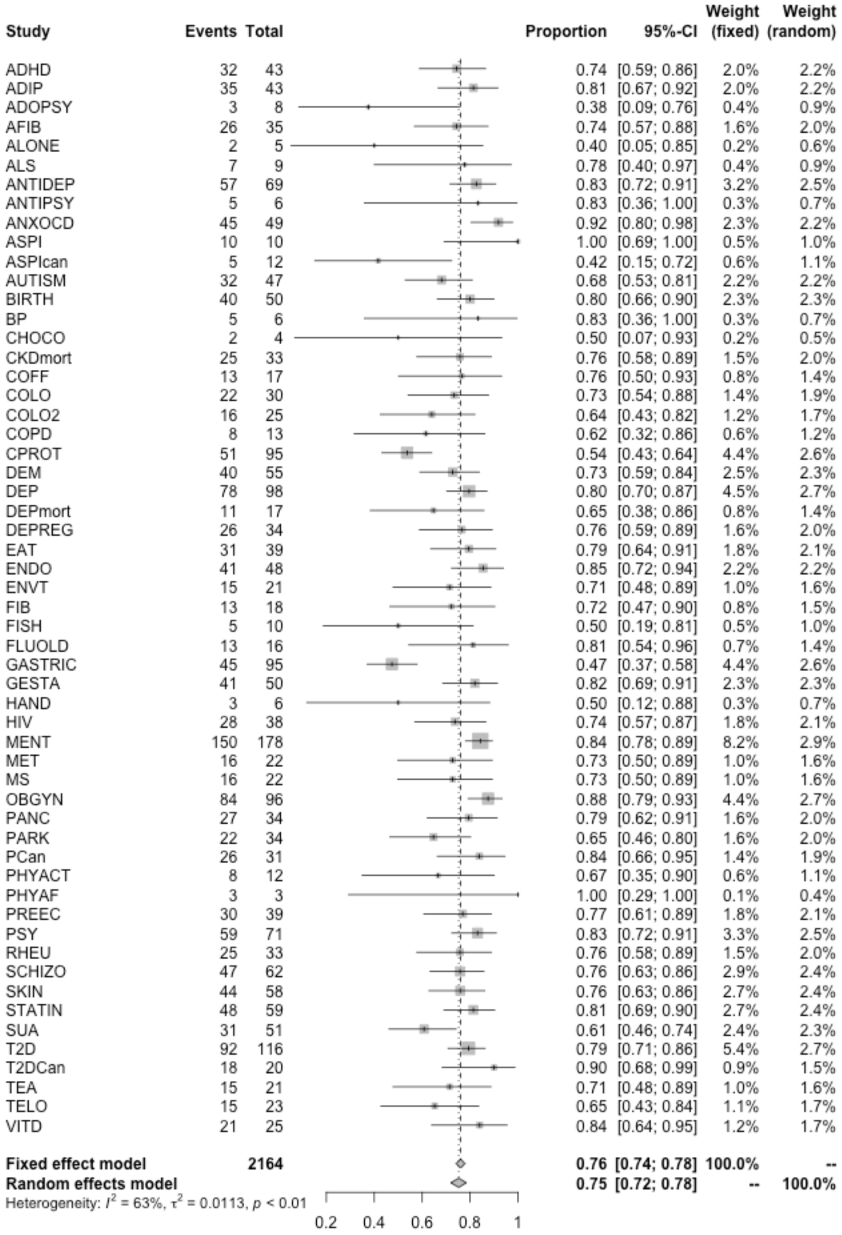

15) No evidence of excess significance P>0.10

Study	Events	Total	Proportion	95%-CI	Weight (fixed)	Weight (random)
ADHD	41	45	0.91	[0.79; 0.98]	2.2%	2.1%
ADIP	46	48	0.96	[0.86; 0.99]	2.3%	2.2%
ADOPSY	7	9	0.78	[0.40; 0.97]	0.4%	1.4%
AFIB	23	35	0.66	[0.48; 0.81]	1.7%	2.1%
ALONE	1	2	0.50	[0.01; 0.99]	0.1%	0.5%
ALS	8	9	0.89	[0.52; 1.00]	0.4%	1.4%
ANTIDEP	56	66	0.85	[0.74; 0.92]	3.2%	2.2%
ANTIPSY	6	6	1.00	[0.54; 1.00]	0.3%	1.1%
ANXOCD	55	69	0.80	[0.68; 0.88]	3.4%	2.3%
ASPI	8	10	0.80	[0.44; 0.97]	0.5%	1.4%
ASPIcan	5	12	0.42	[0.15; 0.72]	0.6%	1.5%
AUTISM	29	31	0.94	[0.79; 0.99]	1.5%	2.0%
BIRTH	35	47	0.74	[0.60; 0.86]	2.3%	2.2%
BP	4	5	0.80	[0.28; 0.99]	0.2%	1.0%
CHOCO	1	1	1.00	[0.03; 1.00]	0.0%	0.3%
CKDmort	32	33	0.97	[0.84; 1.00]	1.6%	2.0%
COFF	14	17	0.82	[0.57; 0.96]	0.8%	1.7%
COLO	17	31	0.55	[0.36; 0.73]	1.5%	2.0%
COLO2	6	9	0.67	[0.30; 0.93]	0.4%	1.4%
COPD	7	11	0.64	[0.31; 0.89]	0.5%	1.5%
CPROT	54	95	0.57	[0.46; 0.67]	4.6%	2.3%
DEM	40	55	0.73	[0.59; 0.84]	2.7%	2.2%
DEP	63	78	0.81	[0.70; 0.89]	3.8%	2.3%
DEPmort	6	17	0.35	[0.14; 0.62]	0.8%	1.7%
EAT	24	33	0.73	[0.54; 0.87]	1.6%	2.0%
ENDO	48	56	0.86	[0.74; 0.94]	2.7%	2.2%
ENVT	15	16	0.94	[0.70; 1.00]	0.8%	1.7%
FIB	16	18	0.89	[0.65; 0.99]	0.9%	1.8%
FISH	1	12	0.08	[0.00; 0.38]	0.6%	1.5%
FLUOLD	17	18	0.94	[0.73; 1.00]	0.9%	1.8%
GASTRIC	82	95	0.86	[0.78; 0.93]	4.6%	2.3%
GESTA	39	51	0.76	[0.63; 0.87]	2.5%	2.2%
HAND	2	3	0.67	[0.09; 0.99]	0.1%	0.7%
HIV	8	24	0.33	[0.16; 0.55]	1.2%	1.9%
MENT	165	178	0.93	[0.88; 0.96]	8.7%	2.4%
MET	12	23	0.52	[0.31; 0.73]	1.1%	1.9%
MG	7	9	0.78	[0.40; 0.97]	0.4%	1.4%
MS	19	20	0.95	[0.75; 1.00]	1.0%	1.8%
OBGYN	91	94	0.97	[0.91; 0.99]	4.6%	2.3%
PANC	18	44	0.41	[0.26; 0.57]	2.1%	2.1%
PARK	20	35	0.57	[0.39; 0.74]	1.7%	2.1%
PCan	27	34	0.79	[0.62; 0.91]	1.7%	2.0%
PHYACT	9	13	0.69	[0.39; 0.91]	0.6%	1.6%
PHYAF	2	2	1.00	[0.16; 1.00]	0.1%	0.5%
PREEC	26	39	0.67	[0.50; 0.81]	1.9%	2.1%
PSY	48	63	0.76	[0.64; 0.86]	3.1%	2.2%
RHEU	28	33	0.85	[0.68; 0.95]	1.6%	2.0%
SCHIZO	38	58	0.66	[0.52; 0.78]	2.8%	2.2%
SKIN	29	41	0.71	[0.54; 0.84]	2.0%	2.1%
STATIN	60	65	0.92	[0.83; 0.97]	3.2%	2.2%
SUA	49	56	0.88	[0.76; 0.95]	2.7%	2.2%
T2D	80	114	0.70	[0.61; 0.78]	5.6%	2.3%
T2DCan	16	20	0.80	[0.56; 0.94]	1.0%	1.8%
TELO	13	15	0.87	[0.60; 0.98]	0.7%	1.7%
VITD	26	29	0.90	[0.73; 0.98]	1.4%	2.0%
**Fixed effect model**		**2052**	**0.80**	**[0.78; 0.82]**	**100.0%**	**--**
**Random effects model**			**0.78**	**[0.73; 0.82]**	**--**	**100.0%**

Heterogeneity: $I^2 = 83\%$, $\tau^2 = 0.0333$, $p < 0.01$

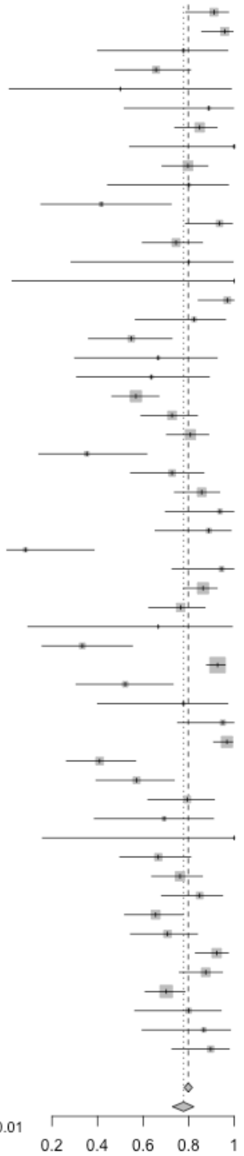

ADHD: Risk factor for attention deficit hyperactivity disorder ; ADIP: Adiposity and cancer outcomes; ADOPSY: Risk and protective factors for mental disorders with onset in childhood/adolescence; AFIB: Environmental factors and serum biomarkers for atrial fibrillation; ALONE: Factors associated to loneliness; ALS: Risk factor for amyotrophic lateral sclerosis; ANTIDEP: Antidepressant and adverse events; ANTIPSY: Antipsychotics and life-threatening events; ANXOCD: Risk and protective factors for anxiety and obsessive compulsive disorders; ASPI: Low-dose aspirin and health outcomes; ASPIcan: Aspirin and cancer outcomes; AUTISM: Environmental risk factors and biomarkers for autism spectrum disorder; BIRTH: Birth weight and later life events; BP: Environmental risk factors for bipolar disorder; CHOCO: Chocolate and health outcomes; CKDmort: Chronic kidney disease and mortality; COFF: Coffee and cancer risk; COLO: Risk factors for colorectal cancer metastasis and recurrence; COLO2: Non-genetic biomarkers and colorectal cancer risk; COPD: Risk factors for chronic obstructive pulmonary disease; CPROT: C-reactive protein and health outcomes; DEM: Environmental risk factors for dementia; DEP: Risk factors for depression; DEPmort: Depression and mortality; DEPPREG: Antidepressants during pregnancy and neonatal outcomes; EAT: Risk factors for eating disorders; ENDO: Risk factors for endometrial cancer; ENVT: Environmental risk factors for obesity; FIB: Dietary Fiber and health

outcomes; FISH: Fish and _-3 Fatty Acids consumptions and cancer outcomes; FLUOLD: Influenza vaccine in elderly and health outcomes; GASTRIC: Prognostic biomarkers for gastric cancer; GESTA: Risk factors for gestational diabetes; HAND: Handgrip strength and health outcomes; HIV: Human immunodeficiency virus infections and health outcomes; MENT: Peripheral biomarkers and major mental disorders; MET: Metformin and cancer outcomes; MG: Magnesium and health outcomes; MS: Environmental risk factors for multiple sclerosis; OBGYN: Obesity and gynecology/obstetric outcomes; PANC: Prognostic biomarkers for pancreatic ductal adenocarcinoma; PARK: Environmental risk factors and Parkinson's; PCan: Risk and protective factors for prostate cancer; PHYACT: Physical activity and cancer outcomes; PHYAF: Physical activity and atrial fibrillation outcomes; PREEC: Non-genetic risk factors for pre-eclampsia; PSY: Risk and protective factors for psychosis; RHEU: Environmental risk factors for rheumatic diseases; SCHIZO: Risk factors and peripheral biomarkers for schizophrenia spectrum disorders; SKIN: Non-genetic risk factors for skin cancer; STATIN: Statins and multiple non-cardiovascular outcomes; SUA: Serum uric acid and health outcomes; T2D: Risk factors for type 2 diabetes mellitus; T2DCan: Type 2 diabetes mellitus and cancer; TEA: Tea consumption and cancer; TELO: Telomere length and health outcomes; VITD: Vitamin D and health outcome
