# Supplementary material for: Validity of observational evidence on putative risk and protective factors: appraisal of 3744 meta-analyses on 57 topics
Source: BMC Med. 2021 Jul 6;19:157. doi: 10.1186/s12916-021-02020-6 (PMC8259334; doi:10.1186/s12916-021-02020-6)
Supplement: Supplementary file 5 — Additional file 5: Table 1. Kappa matrix across credibility criteria (below) and meta-analyzed across umbrella reviews (above). [file 12916_2021_2020_MOESM5_ESM.pdf]

Additional file 5: Table 1 Kappa matrix across credibility criteria (below) and meta-analyzed across umbrella reviews (above)

	**P < 10-6**	**P< 10-3**	**Number of cases**	**Largest study**	**Prediction interval**	**I² > 50%**	**Small study effect**	**Excess of significance**
**P < 10-6**		0.43<br>[0.39; 0.48]<br>N=51; I2=50%	0.07<br>[0.04; 0.10]<br>N=46; I2=40%	0.16<br>[0.12; 0.19]<br>N=40; I2=47%	0.44<br>[0.35; 0.53]<br>N=52; I2=88%	0.03<br>[-0.02; 0.07]<br>N=52; I2=38%	-0.04<br>[-0.07; 0.00]<br>N=51; I2=30%	0.02<br>[0.00; 0.04]<br>N=49; I2=22%
**P< 10-3**	0.5<br>[0.47; 0.55]<br>N=2289		0.10<br>[0.05; 0.14]<br>N=47; I2=51%	0.30<br>[0.25; 0.35]<br>N=39; I2=27%	0.25<br>[0.21; 0.29]<br>N=53; I2=39%	0.03<br>[-0.02; 0.08]<br>N=53; I2=43%	0.06<br>[-0.10; -0.02]<br>N=52; I2=18%	-0.02<br>[-0.06; 0.02]<br>N=51; I2=44%
**Number of cases**	0.08<br>[0.04; 0.12]<br>N=2107	0.15<br>[0.11; 0.2]<br>N=2107		0.00<br>[-0.06; 0.05]<br>N=36; I2=61%	0.04<br>[0.01; 0.06]<br>N=47; I2=39%	-0.13<br>[-0.18; -0.09]<br>N=49; I2=56%	-0.11<br>[-0.15; -0.06]<br>N=45; I2=70%	-0.06<br>[-0.09; -0.03]<br>N=49; I2=42%
**Largest studies**	0.18<br>[0.16; 0.21]<br>N=1781	0.33<br>[0.28; 0.37]<br>N=1781	0.03<br>[-0.01; 0.08]<br>N=1678		0.10<br>[0.07; 0.14]<br>N=41; I2=38%	-0.05<br>[-0.10; 0.00]<br>N=42; I2=52%	0.10<br>[0.04; 0.16]<br>N=42; I2=48%	0.12<br>[0.05; 0.19]<br>N=37; I2=72%
**Prediction interval**	0.47<br>[0.43; 0.51]<br>N=2136	0.25<br>[0.28;0.31]<br>N=2136	0.06<br>[0.03; 0.1]<br>N=1978	0.12<br>[0.09; 0.15]<br>N=1642		0.30<br>[0.24; 0.36]<br>N=54; I2=63%	0.06<br>[0.03; 0.08]<br>N=53; I2=28%	0.04<br>[0.02; 0.07]<br>N=49; I2=37%
**I² > 50%**	0.01<br>[-0.03; 0.05]<br>N=2277	0.04<br>[-0.00; 0.08]<br>N=2277	-0.09<br>[-0.13; -0.04]<br>N=2099	-0.06<br>[-0.1; -0.02]<br>N=1772	0.33<br>[0.29; 0.37]<br>N=2136		0.13<br>[0.09; 0.18]<br>N=52; I2=50%	0.09<br>[0.05; 0.13]<br>N=50; I2=64%
**Small study effect**	-0.05<br>[-0.08; -0.02]<br>N=2164	-0.05<br>[-0.09;- 0.01]<br>N=2164	-0.05<br>[-0.09; -0.01]<br>N=2000	0.15<br>[0.1; 0.2]<br>N=1679	0.04<br>[0.02; 0.07]<br>N=2120	0.12<br>[0.09; 0.16]<br>N=2163		0.14<br>[0.07; 0.22]<br>N=46; I2=76%
**Excess of significance**	0.04<br>[0.01; 0.07]<br>N=2052	0.02<br>[-0.02; 0.06]<br>N=2052	-0.03<br>[-0.07; 0.01]<br>N=1941	0.19<br>[0.13; 0.24]<br>N=1608	0.06<br>[0.03; 0.09]<br>N=1943	0.09<br>[0.06; 0.13]<br>N=2043	0.21<br>[0.16; 0.26]<br>N=1963	
