# Supplementary material for: Validity of observational evidence on putative risk and protective factors: appraisal of 3744 meta-analyses on 57 topics
Source: BMC Med. 2021 Jul 6;19:157. doi: 10.1186/s12916-021-02020-6 (PMC8259334; doi:10.1186/s12916-021-02020-6)
Supplement: Supplementary file 6 — Additional file 6: Figures 16 to 43. Forest plots of the meta-analyses of kappa estimated for each individual association. [file 12916_2021_2020_MOESM6_ESM.pdf]

Additional file 6: Figure 16 to 43 Forest plots of the meta-analyses of kappa estimated for each individual association.

16) Number of cases and excess of significance bias

Study	TE	seTE	95%–CI	Weight (fixed)	Weight (random)
ADHD	0.03	0.0982	0.03 [−0.16; 0.22]	0.0%	2.0%
ADIP	−0.07	0.0398	−0.07 [−0.15; 0.01]	0.2%	5.6%
ADOPSY	0.40	0.3919	0.40 [−0.37; 1.17]	0.0%	0.2%
AFIB	0.09	0.1449	0.09 [−0.19; 0.38]	0.0%	1.0%
ALONE	.	.		0.0%	0.0%
ALS	−0.20	0.1649	−0.20 [−0.52; 0.12]	0.0%	0.8%
ANTIDEP	0.10	0.1438	0.10 [−0.18; 0.38]	0.0%	1.0%
ANTIPSY	.	.		0.0%	0.0%
ANXOCD	0.06	0.0620	0.06 [−0.06; 0.18]	0.1%	3.7%
ASPI	0.18	0.1920	0.18 [−0.19; 0.56]	0.0%	0.6%
AUTISM	−0.11	0.0539	−0.11 [−0.21; 0.00]	0.1%	4.3%
BIRTH	0.14	0.1606	0.14 [−0.18; 0.45]	0.0%	0.8%
BP	−0.25	0.1712	−0.25 [−0.59; 0.09]	0.0%	0.8%
CHOCO	.	.		0.0%	0.0%
CKDmort	−0.06	0.0485	−0.06 [−0.15; 0.04]	0.1%	4.8%
COFF	.	.		0.0%	0.0%
COLO	−0.16	0.1370	−0.16 [−0.43; 0.11]	0.0%	1.1%
COLO2	0.00	0.2667	0.00 [−0.52; 0.52]	0.0%	0.3%
COPD	−0.17	0.1489	−0.17 [−0.46; 0.12]	0.0%	1.0%
CPROT	−0.21	0.0969	−0.21 [−0.40; −0.02]	0.0%	2.0%
DEM	0.01	0.1341	0.01 [−0.25; 0.27]	0.0%	1.2%
DEP	0.09	0.1303	0.09 [−0.17; 0.34]	0.0%	1.2%
DEPmort	0.04	0.2356	0.04 [−0.42; 0.50]	0.0%	0.4%
EAT	−0.01	0.1016	−0.01 [−0.21; 0.19]	0.0%	1.9%
ENDO	−0.09	0.1090	−0.09 [−0.30; 0.13]	0.0%	1.7%
ENVT	−0.11	0.0828	−0.11 [−0.28; 0.05]	0.0%	2.5%
FIB	−0.15	0.0748	−0.15 [−0.30; −0.01]	0.1%	2.9%
FISH	−0.25	0.2531	−0.25 [−0.74; 0.25]	0.0%	0.4%
FLUOLD	−0.11	0.0961	−0.11 [−0.29; 0.08]	0.0%	2.0%
GASTRIC	−0.07	0.0502	−0.07 [−0.17; 0.02]	0.1%	4.6%
GESTA	−0.06	0.1280	−0.06 [−0.31; 0.19]	0.0%	1.3%
HAND	−0.50	0.3062	−0.50 [−1.10; 0.10]	0.0%	0.3%
HIV	−0.41	0.1633	−0.41 [−0.73; −0.09]	0.0%	0.8%
MENT	0.00	0.0017	0.00 [0.00; 0.01]	98.1%	8.9%
MET	−0.23	0.1985	−0.23 [−0.62; 0.16]	0.0%	0.6%
MG	−0.14	0.1000	−0.14 [−0.34; 0.05]	0.0%	1.9%
MS	0.10	0.0932	0.10 [−0.09; 0.28]	0.0%	2.1%
OBGYN	−0.06	0.0314	−0.06 [−0.12; 0.00]	0.3%	6.5%
PANC	−0.05	0.1141	−0.05 [−0.27; 0.18]	0.0%	1.5%
PARK	0.02	0.1576	0.02 [−0.29; 0.33]	0.0%	0.9%
PCan	0.04	0.1726	0.04 [−0.30; 0.38]	0.0%	0.7%
PHYACT	−0.36	0.1357	−0.36 [−0.62; −0.09]	0.0%	1.1%
PREEC	0.07	0.1589	0.07 [−0.24; 0.38]	0.0%	0.9%
PSY	−0.07	0.0922	−0.07 [−0.25; 0.11]	0.0%	2.2%
RHEU	−0.31	0.1009	−0.31 [−0.50; −0.11]	0.0%	1.9%
SCHIZO	0.05	0.0895	0.05 [−0.13; 0.22]	0.0%	2.2%
SKIN	−0.09	0.0585	−0.09 [−0.21; 0.02]	0.1%	3.9%
STATIN	−0.08	0.0679	−0.08 [−0.22; 0.05]	0.1%	3.3%
SUA	−0.15	0.1006	−0.15 [−0.35; 0.05]	0.0%	1.9%
T2D	−0.00	0.0630	−0.00 [−0.13; 0.12]	0.1%	3.6%
T2DCan	−0.09	0.0732	−0.09 [−0.23; 0.06]	0.1%	3.0%
TELO	0.29	0.1792	0.29 [−0.07; 0.64]	0.0%	0.7%
VITD	0.11	0.1360	0.11 [−0.15; 0.38]	0.0%	1.1%
**Fixed effect model**			0.00 [0.00; 0.00]	100.0%	—
**Random effects model**			−0.06 [−0.09; −0.03]	—	100.0%

Heterogeneity: $I^2 = 42\%$, $\tau^2 = 0.0027$, $p < 0.01$

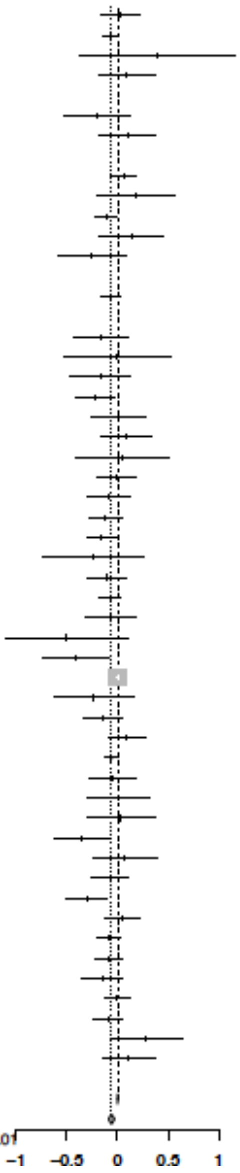

17) Number of cases and I^2 > 50%

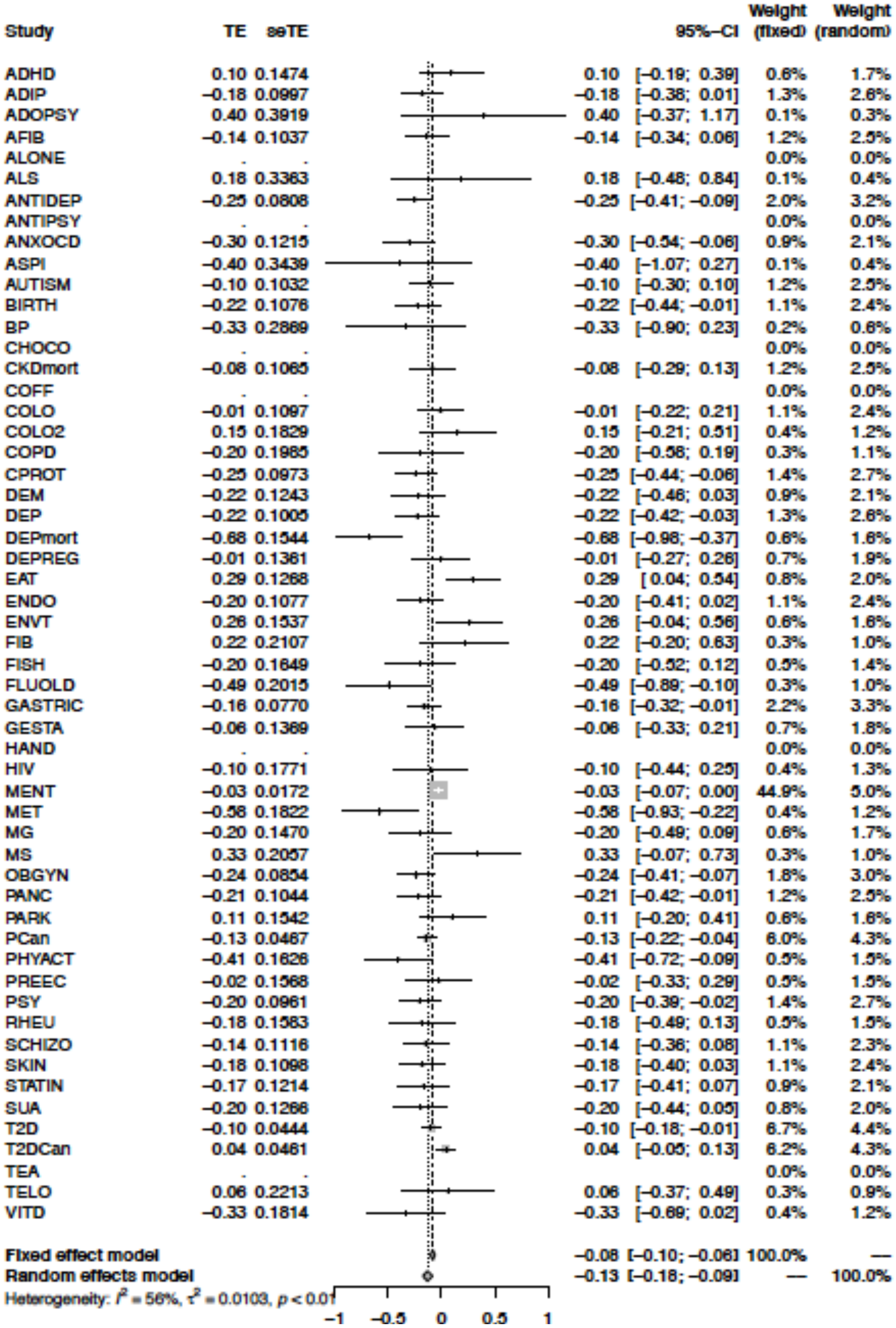

18) Number of cases and largest studies

Study	TE	seTE	95%-CI		Weight (fixed)	Weight (random)
ADHD	0.22	0.1391	0.22	[−0.05; 0.49]	1.1%	2.2%
ADIP	−0.14	0.0423	−0.14	[−0.22; −0.06]	11.6%	4.9%
AFIB	−0.11	0.0428	−0.11	[−0.19; −0.02]	11.3%	4.9%
ALONE	.	.			0.0%	0.0%
ALS	0.73	0.2474	0.73	[0.24; 1.21]	0.3%	0.9%
ANTIDEP	0.13	0.1172	0.13	[−0.10; 0.36]	1.5%	2.6%
ANTIPSY	.	.			0.0%	0.0%
ANXOCD	0.11	0.0475	0.11	[0.01; 0.20]	9.2%	4.8%
AUTISM	−0.22	0.0588	−0.22	[−0.34; −0.10]	6.0%	4.4%
BIRTH	0.13	0.1407	0.13	[−0.15; 0.40]	1.0%	2.1%
BP	.	.			0.0%	0.0%
CHOCO	.	.			0.0%	0.0%
COFF	.	.			0.0%	0.0%
CPROT	−0.14	0.0784	−0.14	[−0.30; 0.01]	3.4%	3.7%
DEM	0.23	0.1378	0.23	[−0.04; 0.51]	1.1%	2.2%
DEPmort	0.08	0.2138	0.08	[−0.34; 0.50]	0.5%	1.2%
DEPREG	0.20	0.1975	0.20	[−0.19; 0.58]	0.5%	1.3%
EAT	0.03	0.0965	0.03	[−0.15; 0.22]	2.2%	3.2%
ENDO	−0.17	0.1003	−0.17	[−0.36; 0.03]	2.1%	3.1%
ENVT	−0.21	0.1005	−0.21	[−0.40; −0.01]	2.1%	3.1%
FIB	−0.04	0.1964	−0.04	[−0.43; 0.34]	0.5%	1.3%
FLUOLD	0.46	0.2218	0.46	[0.03; 0.90]	0.4%	1.1%
GASTRIC	−0.01	0.0548	−0.01	[−0.12; 0.09]	6.9%	4.5%
GESTA	0.22	0.1367	0.22	[−0.05; 0.49]	1.1%	2.2%
HAND	.	.			0.0%	0.0%
MET	0.27	0.2044	0.27	[−0.13; 0.67]	0.5%	1.3%
MS	−0.13	0.1720	−0.13	[−0.46; 0.21]	0.7%	1.6%
OBGYN	0.07	0.0887	0.07	[−0.10; 0.25]	2.6%	3.4%
PANC	0.03	0.0595	0.03	[−0.08; 0.15]	5.8%	4.4%
PARK	−0.01	0.1534	−0.01	[−0.31; 0.29]	0.9%	1.9%
PCan	−0.21	0.0888	−0.21	[−0.39; −0.04]	2.6%	3.4%
PHYACT	−0.05	0.2543	−0.05	[−0.55; 0.44]	0.3%	0.9%
PREEC	0.33	0.1564	0.33	[0.02; 0.64]	0.8%	1.9%
PSY	−0.07	0.0955	−0.07	[−0.26; 0.12]	2.3%	3.2%
SCHIZO	0.06	0.0648	0.06	[−0.07; 0.18]	4.9%	4.2%
SKIN	−0.18	0.1016	−0.18	[−0.38; 0.02]	2.0%	3.0%
STATIN	0.14	0.1067	0.14	[−0.06; 0.35]	1.8%	2.9%
SUA	0.02	0.1026	0.02	[−0.18; 0.22]	2.0%	3.0%
T2D	−0.01	0.0762	−0.01	[−0.16; 0.14]	3.6%	3.8%
T2DCan	−0.08	0.0640	−0.08	[−0.21; 0.04]	5.1%	4.2%
TEA	.	.			0.0%	0.0%
TELO	−0.23	0.2010	−0.23	[−0.62; 0.17]	0.5%	1.3%
VITD	−0.20	0.1739	−0.20	[−0.54; 0.14]	0.7%	1.6%
**Fixed effect model**			−0.03	[−0.06; −0.01]	100.0%	—
**Random effects model**			−0.00	[−0.06; 0.05]	—	100.0%

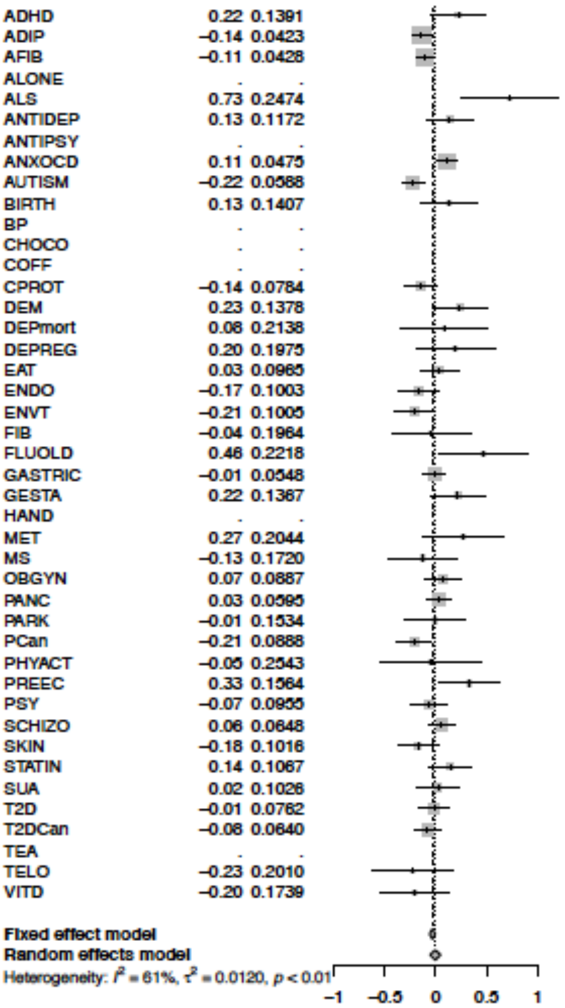

Heterogeneity: $I^2 = 61\%$, $\tau^2 = 0.0120$, $p < 0.01$

19) Number of cases and prediction intervals

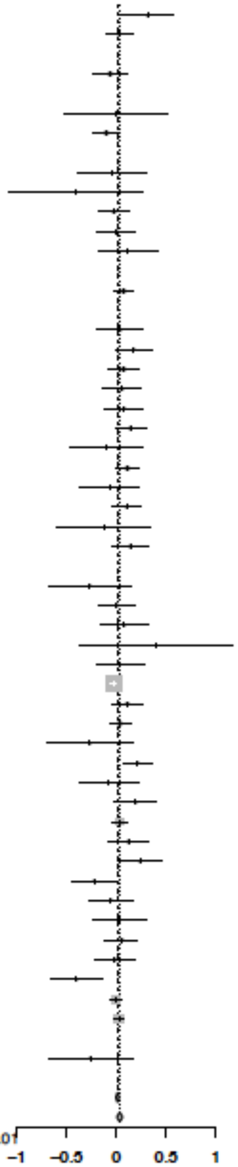

Study	TE	seTE	95%−CI	Weight (fixed)	Weight (random)
ADHD	0.31	0.1310	0.31 [0.06; 0.57]	0.5%	1.2%
ADIP	0.03	0.0685	0.03 [−0.11; 0.16]	1.7%	3.1%
ADOPSY	.	.		0.0%	0.0%
AFIB	−0.06	0.0902	−0.06 [−0.23; 0.12]	1.0%	2.1%
ALONE	.	.		0.0%	0.0%
ALS	0.00	0.2667	0.00 [−0.52; 0.52]	0.1%	0.3%
ANTIDEP	−0.10	0.0676	−0.10 [−0.23; 0.04]	1.8%	3.1%
ANTIPSY	.	.		0.0%	0.0%
ANXOCD	−0.04	0.1731	−0.04 [−0.38; 0.30]	0.3%	0.7%
ASPI	−0.40	0.3439	−0.40 [−1.07; 0.27]	0.1%	0.2%
AUTISM	−0.02	0.0786	−0.02 [−0.18; 0.13]	1.3%	2.6%
BIRTH	0.00	0.0954	0.00 [−0.19; 0.19]	0.9%	1.9%
BP	0.12	0.1516	0.12 [−0.18; 0.41]	0.3%	0.9%
CHOCO	.	.		0.0%	0.0%
CKDmort	0.08	0.0500	0.08 [−0.02; 0.17]	3.2%	4.4%
COFF	.	.		0.0%	0.0%
COLO	0.04	0.1191	0.04 [−0.20; 0.27]	0.6%	1.4%
COLO2	0.18	0.0900	0.18 [0.00; 0.35]	1.0%	2.1%
COPD	0.07	0.0789	0.07 [−0.08; 0.23]	1.3%	2.6%
CPROT	0.05	0.0971	0.05 [−0.14; 0.24]	0.9%	1.9%
DEM	0.08	0.0969	0.08 [−0.11; 0.27]	0.9%	1.9%
DEP	0.14	0.0792	0.14 [−0.01; 0.30]	1.3%	2.6%
DEPmort	−0.10	0.1881	−0.10 [−0.47; 0.27]	0.2%	0.6%
DEPREG	0.12	0.0594	0.12 [0.00; 0.23]	2.3%	3.7%
EAT	−0.06	0.1517	−0.06 [−0.36; 0.23]	0.3%	0.9%
ENDO	0.10	0.0722	0.10 [−0.04; 0.24]	1.5%	2.9%
ENVT	−0.12	0.2371	−0.12 [−0.59; 0.34]	0.1%	0.4%
FIB	0.14	0.0931	0.14 [−0.04; 0.33]	0.9%	2.0%
FISH	.	.		0.0%	0.0%
FLUOLD	−0.26	0.2113	−0.26 [−0.68; 0.15]	0.2%	0.5%
GASTRIC	0.01	0.0924	0.01 [−0.18; 0.19]	0.9%	2.0%
GESTA	0.08	0.1212	0.08 [−0.16; 0.32]	0.5%	1.3%
HAND	0.40	0.3919	0.40 [−0.37; 1.17]	0.1%	0.1%
HIV	0.04	0.1213	0.04 [−0.20; 0.28]	0.5%	1.3%
MENT	−0.03	0.0145	−0.03 [−0.06; 0.00]	38.2%	7.7%
MET	0.11	0.0785	0.11 [−0.05; 0.26]	1.3%	2.6%
MG	0.04	0.0549	0.04 [−0.07; 0.15]	2.7%	4.0%
MS	−0.27	0.2206	−0.27 [−0.70; 0.17]	0.2%	0.5%
OBGYN	0.22	0.0752	0.22 [0.07; 0.36]	1.4%	2.7%
PANC	−0.07	0.1526	−0.07 [−0.37; 0.23]	0.3%	0.9%
PARK	0.19	0.1088	0.19 [−0.02; 0.41]	0.7%	1.6%
PCan	0.04	0.0366	0.04 [−0.04; 0.11]	6.0%	5.6%
PHYACT	0.12	0.1033	0.12 [−0.08; 0.33]	0.8%	1.7%
PREEC	0.24	0.1104	0.24 [0.03; 0.46]	0.7%	1.5%
PSY	−0.22	0.1107	−0.22 [−0.44; −0.01]	0.7%	1.5%
RHEU	−0.06	0.1145	−0.06 [−0.28; 0.17]	0.6%	1.5%
SCHIZO	0.03	0.1340	0.03 [−0.23; 0.30]	0.4%	1.1%
SKIN	0.05	0.0840	0.05 [−0.11; 0.22]	1.1%	2.4%
STATIN	−0.02	0.1008	−0.02 [−0.21; 0.18]	0.8%	1.8%
SUA	−0.40	0.1326	−0.40 [−0.66; −0.14]	0.5%	1.1%
T2D	0.00	0.0300	0.00 [−0.06; 0.06]	8.9%	6.2%
T2DCan	0.03	0.0285	0.03 [−0.03; 0.08]	9.9%	6.4%
TEA	.	.		0.0%	0.0%
VITD	−0.26	0.2171	−0.26 [−0.68; 0.17]	0.2%	0.5%
**Fixed effect model**			0.01 [−0.01; 0.03]	100.0%	—
**Random effects model**			0.04 [0.01; 0.06]	—	100.0%

Heterogeneity: $I^2 = 39\%$, $\tau^2 = 0.0028$, $p < 0.01$

20) Number of cases and small study effects

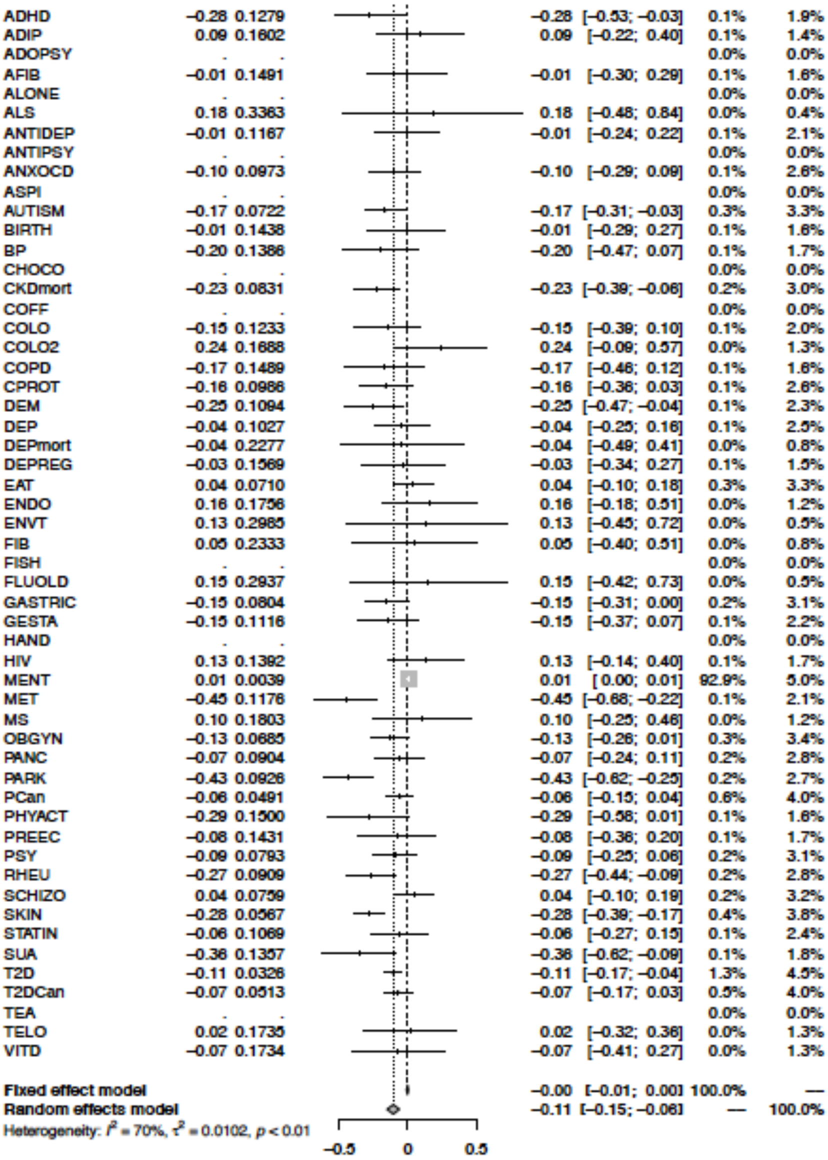

Study	TE	seTE		95%-CI	Weight (fixed)	Weight (random)
ADHD	−0.28	0.1279	−0.28	[−0.53; −0.03]	0.1%	1.9%
ADIP	0.09	0.1602	0.09	[−0.22; 0.40]	0.1%	1.4%
ADOPSY	.	.			0.0%	0.0%
AFIB	−0.01	0.1491	−0.01	[−0.30; 0.29]	0.1%	1.6%
ALONE	.	.			0.0%	0.0%
ALS	0.18	0.3363	0.18	[−0.48; 0.84]	0.0%	0.4%
ANTIDEP	−0.01	0.1167	−0.01	[−0.24; 0.22]	0.1%	2.1%
ANTIPSY	.	.			0.0%	0.0%
ANXOCD	−0.10	0.0973	−0.10	[−0.29; 0.09]	0.1%	2.6%
ASPI	.	.			0.0%	0.0%
AUTISM	−0.17	0.0722	−0.17	[−0.31; −0.03]	0.3%	3.3%
BIRTH	−0.01	0.1438	−0.01	[−0.29; 0.27]	0.1%	1.6%
BP	−0.20	0.1386	−0.20	[−0.47; 0.07]	0.1%	1.7%
CHOCO	.	.			0.0%	0.0%
CKDmort	−0.23	0.0831	−0.23	[−0.39; −0.06]	0.2%	3.0%
COFF	.	.			0.0%	0.0%
COLO	−0.15	0.1233	−0.15	[−0.39; 0.10]	0.1%	2.0%
COLO2	0.24	0.1688	0.24	[−0.09; 0.57]	0.0%	1.3%
COPD	−0.17	0.1489	−0.17	[−0.46; 0.12]	0.1%	1.6%
CPROT	−0.16	0.0986	−0.16	[−0.36; 0.03]	0.1%	2.6%
DEM	−0.25	0.1094	−0.25	[−0.47; −0.04]	0.1%	2.3%
DEP	−0.04	0.1027	−0.04	[−0.25; 0.16]	0.1%	2.5%
DEPmort	−0.04	0.2277	−0.04	[−0.49; 0.41]	0.0%	0.8%
DEPREG	−0.03	0.1569	−0.03	[−0.34; 0.27]	0.1%	1.5%
EAT	0.04	0.0710	0.04	[−0.10; 0.18]	0.3%	3.3%
ENDO	0.16	0.1756	0.16	[−0.18; 0.51]	0.0%	1.2%
ENVT	0.13	0.2985	0.13	[−0.45; 0.72]	0.0%	0.5%
FIB	0.05	0.2333	0.05	[−0.40; 0.51]	0.0%	0.8%
FISH	.	.			0.0%	0.0%
FLUOLD	0.15	0.2937	0.15	[−0.42; 0.73]	0.0%	0.5%
GASTRIC	−0.15	0.0804	−0.15	[−0.31; 0.00]	0.2%	3.1%
GESTA	−0.15	0.1116	−0.15	[−0.37; 0.07]	0.1%	2.2%
HAND	.	.			0.0%	0.0%
HIV	0.13	0.1392	0.13	[−0.14; 0.40]	0.1%	1.7%
MENT	0.01	0.0039	0.01	[0.00; 0.01]	92.9%	5.0%
MET	−0.45	0.1176	−0.45	[−0.68; −0.22]	0.1%	2.1%
MS	0.10	0.1803	0.10	[−0.25; 0.46]	0.0%	1.2%
OBGYN	−0.13	0.0685	−0.13	[−0.26; 0.01]	0.3%	3.4%
PANC	−0.07	0.0904	−0.07	[−0.24; 0.11]	0.2%	2.8%
PARK	−0.43	0.0926	−0.43	[−0.62; −0.25]	0.2%	2.7%
PCan	−0.06	0.0491	−0.06	[−0.15; 0.04]	0.6%	4.0%
PHYACT	−0.29	0.1500	−0.29	[−0.58; 0.01]	0.1%	1.6%
PREEC	−0.08	0.1431	−0.08	[−0.36; 0.20]	0.1%	1.7%
PSY	−0.09	0.0793	−0.09	[−0.25; 0.06]	0.2%	3.1%
RHEU	−0.27	0.0909	−0.27	[−0.44; −0.09]	0.2%	2.8%
SCHIZO	0.04	0.0759	0.04	[−0.10; 0.19]	0.2%	3.2%
SKIN	−0.28	0.0567	−0.28	[−0.39; −0.17]	0.4%	3.8%
STATIN	−0.06	0.1069	−0.06	[−0.27; 0.15]	0.1%	2.4%
SUA	−0.36	0.1357	−0.36	[−0.62; −0.09]	0.1%	1.8%
T2D	−0.11	0.0326	−0.11	[−0.17; −0.04]	1.3%	4.5%
T2DCan	−0.07	0.0513	−0.07	[−0.17; 0.03]	0.5%	4.0%
TEA	.	.			0.0%	0.0%
TELO	0.02	0.1735	0.02	[−0.32; 0.36]	0.0%	1.3%
VITD	−0.07	0.1734	−0.07	[−0.41; 0.27]	0.0%	1.3%
**Fixed effect model**			−0.00	[−0.01; 0.00]	100.0%	—
**Random effects model**			−0.11	[−0.15; −0.06]	—	100.0%

Heterogeneity: $I^2 = 70\%$, $\tau^2 = 0.0102$, $p < 0.01$

21) I^2 > 50% and excess of significance bias

Study	TE	seTE		95%–CI	Weight (fixed)	Weight (random)
ADHD	0.08	0.0823	0.08	[−0.08; 0.24]	1.2%	2.7%
ADIP	0.02	0.0689	0.02	[−0.12; 0.15]	1.8%	3.1%
ADOPSY	−0.05	0.2616	−0.05	[−0.56; 0.47]	0.1%	0.5%
AFIB	0.19	0.1418	0.19	[−0.08; 0.47]	0.4%	1.4%
ALONE	.	.			0.0%	0.0%
ALS	−0.17	0.1273	−0.17	[−0.42; 0.08]	0.5%	1.7%
ANTIDEP	0.16	0.0817	0.16	[0.00; 0.32]	1.3%	2.7%
ANTIPSY	.	.			0.0%	0.0%
ANXOCD	0.20	0.1163	0.20	[−0.03; 0.43]	0.6%	1.9%
ASPI	−0.07	0.2283	−0.07	[−0.52; 0.38]	0.2%	0.7%
ASPIcan	−0.03	0.2881	−0.03	[−0.59; 0.54]	0.1%	0.5%
AUTISM	0.20	0.1245	0.20	[−0.05; 0.44]	0.5%	1.7%
BIRTH	−0.08	0.1344	−0.08	[−0.34; 0.18]	0.5%	1.5%
BP	0.29	0.2738	0.29	[−0.25; 0.82]	0.1%	0.5%
CHOCO	.	.			0.0%	0.0%
CKDmort	0.02	0.0240	0.02	[−0.02; 0.07]	14.6%	4.4%
COFF	−0.02	0.2160	−0.02	[−0.44; 0.41]	0.2%	0.7%
COLO	−0.07	0.1716	−0.07	[−0.41; 0.27]	0.3%	1.1%
COLO2	0.25	0.1849	0.25	[−0.11; 0.61]	0.2%	1.0%
COPD	−0.08	0.2031	−0.08	[−0.48; 0.31]	0.2%	0.8%
CPROT	0.51	0.0885	0.51	[0.34; 0.68]	1.1%	2.5%
DEM	0.11	0.1273	0.11	[−0.14; 0.36]	0.5%	1.7%
DEP	0.07	0.0713	0.07	[−0.07; 0.21]	1.7%	3.0%
DEPmort	0.23	0.2434	0.23	[−0.25; 0.70]	0.1%	0.6%
EAT	−0.22	0.1495	−0.22	[−0.52; 0.07]	0.4%	1.3%
ENDO	−0.08	0.1050	−0.08	[−0.28; 0.13]	0.8%	2.1%
ENVT	0.08	0.0783	0.08	[−0.08; 0.23]	1.4%	2.8%
FIB	−0.21	0.1273	−0.21	[−0.46; 0.04]	0.5%	1.7%
FISH	0.06	0.0649	0.06	[−0.07; 0.19]	2.0%	3.2%
FLUOLD	0.09	0.0889	0.09	[−0.08; 0.26]	1.1%	2.5%
GASTRIC	0.06	0.0791	0.06	[−0.09; 0.22]	1.3%	2.8%
GESTA	0.01	0.1200	0.01	[−0.23; 0.24]	0.6%	1.8%
HAND	.	.			0.0%	0.0%
HIV	−0.06	0.1990	−0.06	[−0.45; 0.33]	0.2%	0.9%
MENT	0.02	0.0150	0.02	[−0.01; 0.05]	37.3%	4.6%
MET	0.40	0.1840	0.40	[0.04; 0.76]	0.2%	1.0%
MG	0.73	0.2474	0.73	[0.24; 1.21]	0.1%	0.6%
MS	0.10	0.0970	0.10	[−0.09; 0.29]	0.9%	2.3%
OBGYN	0.02	0.0336	0.02	[−0.05; 0.08]	7.4%	4.2%
PANC	0.31	0.1263	0.31	[0.06; 0.55]	0.5%	1.7%
PARK	0.48	0.1490	0.48	[0.19; 0.77]	0.4%	1.3%
PCan	−0.18	0.0624	−0.18	[−0.30; −0.05]	2.2%	3.3%
PHYACT	0.83	0.1599	0.83	[0.52; 1.14]	0.3%	1.2%
PREEC	0.24	0.1449	0.24	[−0.05; 0.52]	0.4%	1.4%
PSY	0.19	0.1242	0.19	[−0.06; 0.43]	0.5%	1.7%
RHEU	0.06	0.0373	0.06	[−0.01; 0.13]	6.0%	4.1%
SCHIZO	0.17	0.0837	0.17	[0.00; 0.33]	1.2%	2.6%
SKIN	0.12	0.1182	0.12	[−0.11; 0.35]	0.6%	1.8%
STATIN	0.15	0.0638	0.15	[0.02; 0.27]	2.1%	3.2%
SUA	−0.01	0.0640	−0.01	[−0.14; 0.11]	2.0%	3.2%
T2D	0.18	0.0705	0.18	[0.04; 0.31]	1.7%	3.0%
T2DCan	0.19	0.1066	0.19	[−0.02; 0.40]	0.7%	2.1%
TELO	−0.07	0.1475	−0.07	[−0.36; 0.22]	0.4%	1.4%
VITD	0.26	0.1326	0.26	[0.00; 0.52]	0.5%	1.6%
**Fixed effect model**			0.05	[0.03; 0.07]	100.0%	—
**Random effects model**			0.09	[0.05; 0.13]	—	100.0%

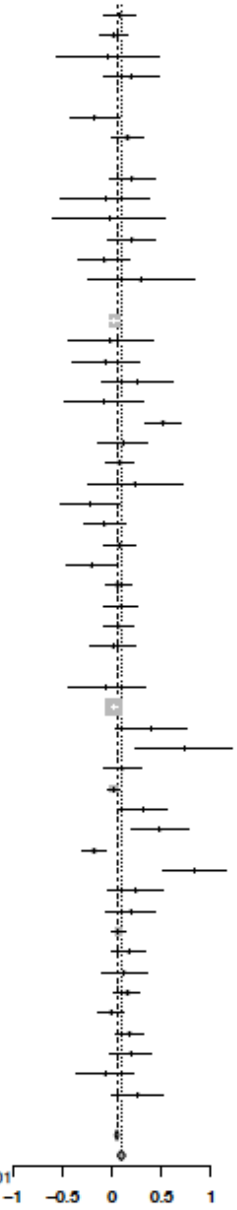

Heterogeneity: $I^2 = 64\%$, $\tau^2 = 0.0088$, $p < 0.01$

22) $I^2 > 50\%$ and small study effects

Study	TE	seTE	95%-CI	Weight (fixed)	Weight (random)
ADHD	0.10	0.1267	0.10 [−0.15; 0.35]	1.1%	1.9%
ADIP	0.22	0.1234	0.22 [−0.02; 0.46]	1.2%	2.0%
ADOPSY	0.47	0.3227	0.47 [−0.17; 1.10]	0.2%	0.4%
AFIB	0.06	0.1295	0.06 [−0.19; 0.32]	1.1%	1.8%
ALONE	−0.36	0.2716	−0.36 [−0.90; 0.17]	0.2%	0.6%
ALS	0.36	0.3665	0.36 [−0.36; 1.08]	0.1%	0.3%
ANTIDEP	0.07	0.0816	0.07 [−0.09; 0.22]	2.6%	3.0%
ANTIPSY	0.08	0.1025	0.08 [−0.12; 0.28]	1.7%	2.4%
ANXOCD	0.09	0.0799	0.09 [−0.07; 0.24]	2.8%	3.0%
ASPI	.	.		0.0%	0.0%
ASPIcan	0.31	0.2772	0.31 [−0.23; 0.86]	0.2%	0.6%
AUTISM	0.23	0.1338	0.23 [−0.03; 0.49]	1.0%	1.8%
BIRTH	0.09	0.1288	0.09 [−0.16; 0.34]	1.1%	1.9%
BP	0.33	0.2889	0.33 [−0.23; 0.90]	0.2%	0.5%
CHOCO	.	.		0.0%	0.0%
CKDmort	0.12	0.0960	0.12 [−0.07; 0.30]	1.9%	2.6%
COFF	−0.11	0.2183	−0.11 [−0.54; 0.31]	0.4%	0.9%
COLO	0.05	0.1833	0.05 [−0.31; 0.41]	0.5%	1.1%
COLO2	0.61	0.1427	0.61 [0.33; 0.89]	0.9%	1.6%
COPD	−0.06	0.1759	−0.06 [−0.41; 0.28]	0.6%	1.2%
CPROT	0.53	0.0870	0.53 [0.36; 0.70]	2.3%	2.8%
DEM	0.34	0.1222	0.34 [0.10; 0.58]	1.2%	2.0%
DEP	0.06	0.0669	0.06 [−0.08; 0.19]	3.9%	3.4%
DEPmort	0.03	0.2027	0.03 [−0.37; 0.42]	0.4%	1.0%
DEPREG	0.09	0.1603	0.09 [−0.22; 0.41]	0.7%	1.4%
EAT	−0.32	0.1258	−0.32 [−0.57; −0.08]	1.1%	1.9%
ENDO	0.10	0.1175	0.10 [−0.13; 0.33]	1.3%	2.1%
ENVT	0.22	0.1612	0.22 [−0.09; 0.54]	0.7%	1.4%
FIB	0.01	0.2291	0.01 [−0.44; 0.46]	0.3%	0.8%
FISH	0.60	0.2319	0.60 [0.15; 1.05]	0.3%	0.8%
FLUOLD	0.19	0.1187	0.19 [−0.04; 0.42]	1.3%	2.0%
GASTRIC	0.23	0.0979	0.23 [0.03; 0.42]	1.8%	2.5%
GESTA	0.04	0.1086	0.04 [−0.17; 0.25]	1.5%	2.3%
HAND	.	.		0.0%	0.0%
HIV	0.13	0.1105	0.13 [−0.09; 0.35]	1.4%	2.2%
MENT	0.03	0.0253	0.03 [−0.02; 0.08]	27.5%	4.6%
MET	0.35	0.1391	0.35 [0.08; 0.63]	0.9%	1.7%
MS	0.30	0.1694	0.30 [−0.03; 0.63]	0.6%	1.3%
OBGYN	−0.01	0.0655	−0.01 [−0.14; 0.12]	4.1%	3.5%
PANC	−0.04	0.1428	−0.04 [−0.32; 0.24]	0.9%	1.6%
PARK	0.16	0.1651	0.16 [−0.16; 0.49]	0.6%	1.3%
PCan	0.35	0.2287	0.35 [−0.10; 0.80]	0.3%	0.8%
PHYACT	0.12	0.2855	0.12 [−0.44; 0.68]	0.2%	0.5%
PREEC	−0.06	0.1332	−0.06 [−0.32; 0.20]	1.0%	1.8%
PSY	0.18	0.1101	0.18 [−0.04; 0.39]	1.5%	2.2%
RHEU	0.11	0.0566	0.11 [0.00; 0.22]	5.5%	3.7%
SCHIZO	0.03	0.0707	0.03 [−0.11; 0.17]	3.5%	3.3%
SKIN	0.20	0.0889	0.20 [0.03; 0.38]	2.2%	2.8%
STATIN	0.20	0.0915	0.20 [0.02; 0.37]	2.1%	2.7%
SUA	0.29	0.0965	0.29 [0.10; 0.48]	1.9%	2.6%
T2D	0.09	0.0601	0.09 [−0.03; 0.20]	4.9%	3.6%
T2DCan	0.09	0.0691	0.09 [−0.04; 0.23]	3.7%	3.3%
TEA	−0.17	0.1931	−0.17 [−0.55; 0.21]	0.5%	1.0%
TELO	0.26	0.1183	0.26 [0.03; 0.49]	1.3%	2.1%
VITD	0.18	0.1502	0.18 [−0.12; 0.47]	0.8%	1.5%
**Fixed effect model**			0.10 [0.08; 0.13]	100.0%	—
**Random effects model**			0.13 [0.09; 0.18]	—	100.0%

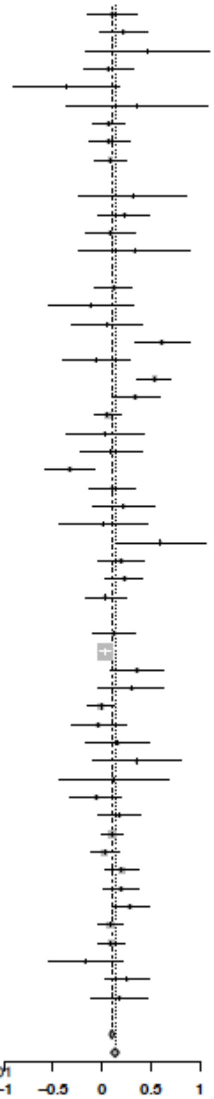

Heterogeneity: $I^2 = 50\%$, $\tau^2 = 0.0100$, $p < 0.01$

23) Largest studies and excess of significance bias

Study	TE	seTE		95%-CI	Weight (fixed)	Weight (random)
ADHD	0.46	0.1573	0.46	[0.16; 0.77]	0.8%	2.5%
ADIP	−0.06	0.0337	−0.06	[−0.13; 0.00]	18.3%	4.7%
AFIB	−0.00	0.1207	−0.00	[−0.24; 0.23]	1.4%	3.1%
ALONE	.	.			0.0%	0.0%
ALS	−0.17	0.1273	−0.17	[−0.42; 0.08]	1.3%	3.0%
ANTIDEP	−0.09	0.1009	−0.09	[−0.28; 0.11]	2.0%	3.5%
ANTIPSY	.	.			0.0%	0.0%
ANXOCD	0.27	0.1410	0.27	[−0.01; 0.55]	1.0%	2.7%
ASPIcan	−0.03	0.2881	−0.03	[−0.59; 0.54]	0.3%	1.1%
AUTISM	0.53	0.2284	0.53	[0.08; 0.98]	0.4%	1.6%
BIRTH	0.50	0.1355	0.50	[0.23; 0.76]	1.1%	2.8%
BP	.	.			0.0%	0.0%
CHOCO	.	.			0.0%	0.0%
COFF	−0.02	0.2160	−0.02	[−0.44; 0.41]	0.4%	1.7%
CPROT	0.06	0.0910	0.06	[−0.12; 0.24]	2.5%	3.7%
DEM	0.24	0.1409	0.24	[−0.04; 0.51]	1.0%	2.7%
DEPmort	−0.05	0.1917	−0.05	[−0.43; 0.33]	0.6%	2.0%
EAT	0.24	0.1735	0.24	[−0.10; 0.58]	0.7%	2.2%
ENDO	0.03	0.1357	0.03	[−0.23; 0.30]	1.1%	2.8%
ENVT	0.45	0.3044	0.45	[−0.15; 1.04]	0.2%	1.0%
FIB	0.06	0.1814	0.06	[−0.30; 0.42]	0.6%	2.1%
FLUOLD	−0.10	0.0826	−0.10	[−0.26; 0.06]	3.1%	3.9%
GASTRIC	0.11	0.1061	0.11	[−0.10; 0.32]	1.8%	3.4%
GESTA	−0.24	0.1014	−0.24	[−0.44; −0.04]	2.0%	3.5%
HAND	.	.			0.0%	0.0%
MET	0.12	0.2041	0.12	[−0.28; 0.52]	0.5%	1.8%
MS	−0.09	0.0732	−0.09	[−0.23; 0.06]	3.9%	4.1%
OBGYN	−0.05	0.0226	−0.05	[−0.09; −0.01]	40.6%	4.8%
PANC	−0.12	0.1215	−0.12	[−0.36; 0.11]	1.4%	3.1%
PARK	0.60	0.1365	0.60	[0.33; 0.86]	1.1%	2.8%
PCan	−0.21	0.1330	−0.21	[−0.47; 0.05]	1.2%	2.9%
PHYACT	0.49	0.2493	0.49	[0.00; 0.98]	0.3%	1.4%
PREEC	0.21	0.1592	0.21	[−0.11; 0.52]	0.8%	2.4%
PSY	0.26	0.1235	0.26	[0.02; 0.50]	1.4%	3.0%
SCHIZO	0.49	0.1201	0.49	[0.26; 0.73]	1.4%	3.1%
SKIN	0.38	0.1599	0.38	[0.07; 0.69]	0.8%	2.4%
STATIN	0.38	0.1483	0.38	[0.08; 0.67]	0.9%	2.6%
SUA	0.15	0.1661	0.15	[−0.18; 0.47]	0.8%	2.3%
T2D	0.21	0.0960	0.21	[0.02; 0.40]	2.3%	3.6%
T2DCan	0.48	0.2542	0.48	[−0.02; 0.98]	0.3%	1.4%
TELO	0.06	0.2075	0.06	[−0.34; 0.47]	0.5%	1.8%
VITD	0.03	0.1657	0.03	[−0.29; 0.36]	0.8%	2.3%
**Fixed effect model**			0.01	[−0.02; 0.04]	100.0%	—
**Random effects model**			0.12	[0.05; 0.19]	—	100.0%

Heterogeneity: $I^2 = 72\%$, $\tau^2 = 0.0246$, $p < 0.01$

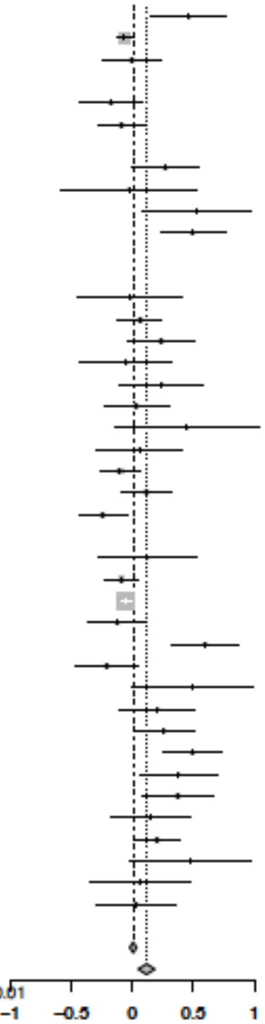

24) Largest studies and $I^2 > 50\%$

Study	TE	seTE	95%–CI	Weight (fixed)	Weight (random)
ADHD	0.12	0.1253	0.12 [−0.12; 0.37]	1.8%	2.4%
ADIP	−0.20	0.0793	−0.20 [−0.36; −0.04]	4.6%	3.6%
AFIB	−0.08	0.0878	−0.08 [−0.25; 0.09]	3.8%	3.3%
ALONE	0.12	0.1516	0.12 [−0.18; 0.41]	1.3%	1.9%
ALS	−0.29	0.1369	−0.29 [−0.55; −0.02]	1.5%	2.2%
ANTIDEP	−0.40	0.1004	−0.40 [−0.59; −0.20]	2.9%	3.0%
ANTIPSY	0.08	0.1025	0.08 [−0.12; 0.28]	2.8%	2.9%
ANXOCD	−0.21	0.0810	−0.21 [−0.37; −0.05]	4.4%	3.5%
ASPIcan	−0.37	0.2571	−0.37 [−0.88; 0.13]	0.4%	0.9%
AUTISM	0.20	0.1211	0.20 [−0.04; 0.43]	2.0%	2.5%
BIRTH	−0.15	0.1348	−0.15 [−0.42; 0.11]	1.6%	2.2%
BP	.	.		0.0%	0.0%
CHOCO	0.00	0.5000	0.00 [−0.98; 0.98]	0.1%	0.3%
COFF	−0.03	0.2410	−0.03 [−0.50; 0.44]	0.5%	1.0%
CPROT	0.12	0.0876	0.12 [−0.05; 0.29]	3.8%	3.3%
DEM	0.00	0.1283	0.00 [−0.25; 0.25]	1.8%	2.4%
DEPmort	−0.05	0.1917	−0.05 [−0.43; 0.33]	0.8%	1.4%
DEPREG	−0.12	0.1450	−0.12 [−0.40; 0.17]	1.4%	2.1%
EAT	−0.12	0.1393	−0.12 [−0.39; 0.15]	1.5%	2.2%
ENDO	−0.28	0.0939	−0.28 [−0.46; −0.10]	3.3%	3.2%
ENVT	−0.14	0.1363	−0.14 [−0.41; 0.13]	1.6%	2.2%
FIB	0.06	0.2366	0.06 [−0.40; 0.53]	0.5%	1.0%
FLUOLD	−0.20	0.1538	−0.20 [−0.50; 0.10]	1.2%	1.9%
GASTRIC	0.07	0.0955	0.07 [−0.11; 0.26]	3.2%	3.1%
GESTA	−0.31	0.1187	−0.31 [−0.54; −0.07]	2.1%	2.6%
HAND	.	.		0.0%	0.0%
MET	−0.08	0.1909	−0.08 [−0.45; 0.29]	0.8%	1.4%
MS	−0.13	0.1720	−0.13 [−0.46; 0.21]	1.0%	1.6%
OBGYN	−0.11	0.0576	−0.11 [−0.23; 0.00]	8.7%	4.2%
PANC	−0.11	0.1384	−0.11 [−0.38; 0.17]	1.5%	2.2%
PARK	0.31	0.1612	0.31 [−0.01; 0.63]	1.1%	1.8%
PCan	0.24	0.1516	0.24 [−0.06; 0.54]	1.3%	1.9%
PHYACT	0.35	0.2660	0.35 [−0.17; 0.87]	0.4%	0.8%
PREEC	−0.41	0.1481	−0.41 [−0.70; −0.12]	1.3%	2.0%
PSY	−0.11	0.1097	−0.11 [−0.33; 0.10]	2.4%	2.8%
SCHIZO	−0.04	0.0728	−0.04 [−0.19; 0.10]	5.5%	3.8%
SKIN	0.12	0.0888	0.12 [−0.06; 0.29]	3.7%	3.3%
STATIN	0.15	0.0969	0.15 [−0.04; 0.34]	3.1%	3.1%
SUA	−0.06	0.0775	−0.06 [−0.22; 0.09]	4.8%	3.6%
T2D	0.06	0.0539	0.06 [−0.05; 0.17]	10.0%	4.3%
T2DCan	−0.02	0.1173	−0.02 [−0.25; 0.21]	2.1%	2.6%
TEA	0.11	0.1397	0.11 [−0.16; 0.39]	1.5%	2.1%
TELO	0.03	0.1607	0.03 [−0.29; 0.34]	1.1%	1.8%
VITD	0.19	0.1866	0.19 [−0.18; 0.55]	0.8%	1.5%
**Fixed effect model**			−0.05 [−0.08; −0.02]	100.0%	—
**Random effects model**			−0.05 [−0.10; 0.00]	—	100.0%

Heterogeneity: $I^2 = 52\%$, $\tau^2 = 0.0136$, $p < 0.01$

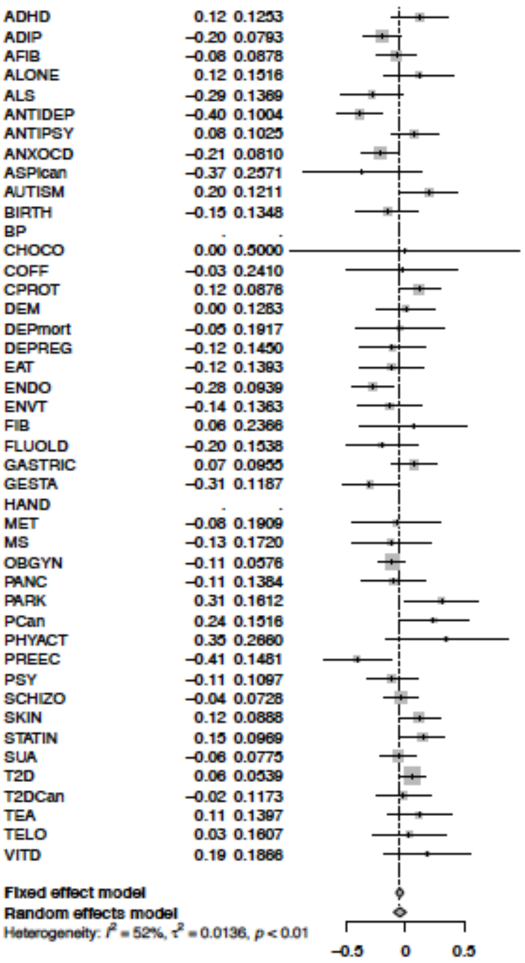

25) Largest studies and prediction intervals

Study	TE	seTE	95%-CI	Weight (fixed)	Weight (random)
ADHD	0.26	0.1025	0.26 [0.05; 0.46]	1.7%	2.4%
ADIP	0.02	0.0884	0.02 [−0.16; 0.19]	2.3%	2.9%
AFIB	0.00	0.0709	0.00 [−0.14; 0.14]	3.6%	3.7%
ALONE	0.55	0.3621	0.55 [−0.16; 1.26]	0.1%	0.3%
ALS	0.25	0.1849	0.25 [−0.11; 0.61]	0.5%	0.9%
ANTIDEP	−0.17	0.0970	−0.17 [−0.36; 0.02]	1.9%	2.6%
ANTIPSY	0.18	0.1920	0.18 [−0.19; 0.56]	0.5%	0.9%
ANXOCD	0.01	0.0718	0.01 [−0.14; 0.15]	3.5%	3.7%
ASPIcan	0.06	0.2431	0.06 [−0.41; 0.54]	0.3%	0.6%
AUTISM	0.22	0.1062	0.22 [0.01; 0.43]	1.6%	2.3%
BIRTH	0.29	0.1146	0.29 [0.06; 0.51]	1.4%	2.0%
BP	.	.		0.0%	0.0%
CHOCO	−0.50	0.3750	−0.50 [−1.23; 0.23]	0.1%	0.3%
COFF	−0.05	0.1917	−0.05 [−0.43; 0.33]	0.5%	0.9%
CPROT	0.17	0.0438	0.17 [0.08; 0.25]	9.5%	5.4%
DEM	0.16	0.0858	0.16 [0.00; 0.33]	2.5%	3.0%
DEPmort	0.16	0.1049	0.16 [−0.04; 0.37]	1.7%	2.3%
DEPREG	−0.11	0.1106	−0.11 [−0.32; 0.11]	1.5%	2.1%
EAT	0.10	0.1059	0.10 [−0.11; 0.30]	1.6%	2.3%
ENDO	0.06	0.0966	0.06 [−0.12; 0.25]	2.0%	2.6%
ENVT	0.02	0.1243	0.02 [−0.22; 0.27]	1.2%	1.8%
FIB	−0.01	0.1880	−0.01 [−0.38; 0.36]	0.5%	0.9%
FLUOLD	−0.25	0.1990	−0.25 [−0.64; 0.14]	0.5%	0.8%
GASTRIC	0.11	0.0738	0.11 [−0.04; 0.25]	3.4%	3.6%
GESTA	−0.00	0.1056	−0.00 [−0.21; 0.20]	1.6%	2.3%
HAND	.	.		0.0%	0.0%
MET	0.13	0.0913	0.13 [−0.05; 0.31]	2.2%	2.8%
MS	0.40	0.1640	0.40 [0.08; 0.72]	0.7%	1.2%
OBGYN	0.11	0.0495	0.11 [0.01; 0.21]	7.5%	5.0%
PANC	0.23	0.0892	0.23 [0.05; 0.40]	2.3%	2.9%
PARK	0.25	0.1506	0.25 [−0.04; 0.55]	0.8%	1.3%
PCan	−0.09	0.1591	−0.09 [−0.40; 0.22]	0.7%	1.2%
PHYACT	0.38	0.1980	0.38 [0.00; 0.77]	0.5%	0.8%
PREEC	0.19	0.1244	0.19 [−0.05; 0.44]	1.2%	1.8%
PSY	0.28	0.0989	0.28 [0.09; 0.47]	1.9%	2.5%
SCHIZO	0.05	0.0616	0.05 [−0.07; 0.17]	4.8%	4.2%
SKIN	0.16	0.0697	0.16 [0.02; 0.29]	3.8%	3.8%
STATIN	0.15	0.0515	0.15 [0.05; 0.25]	6.9%	4.9%
SUA	0.19	0.0691	0.19 [0.05; 0.32]	3.8%	3.8%
T2D	0.12	0.0400	0.12 [0.04; 0.20]	11.4%	5.7%
T2DCan	0.08	0.0604	0.08 [−0.03; 0.20]	5.0%	4.3%
TEA	−0.24	0.1003	−0.24 [−0.43; −0.04]	1.8%	2.5%
VITD	0.20	0.1932	0.20 [−0.18; 0.58]	0.5%	0.9%
**Fixed effect model**			0.11 [0.08; 0.13]	100.0%	--
**Random effects model**			0.10 [0.07; 0.14]	--	100.0%

Heterogeneity: $I^2 = 38\%$, $\tau^2 = 0.0048$, $p < 0.01$

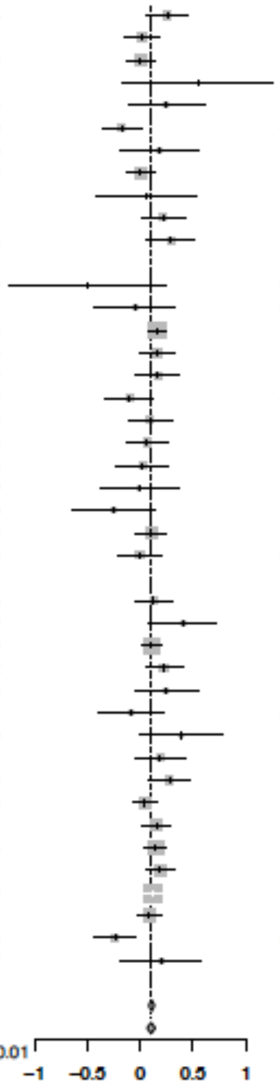

26) Largest studies and small study effects

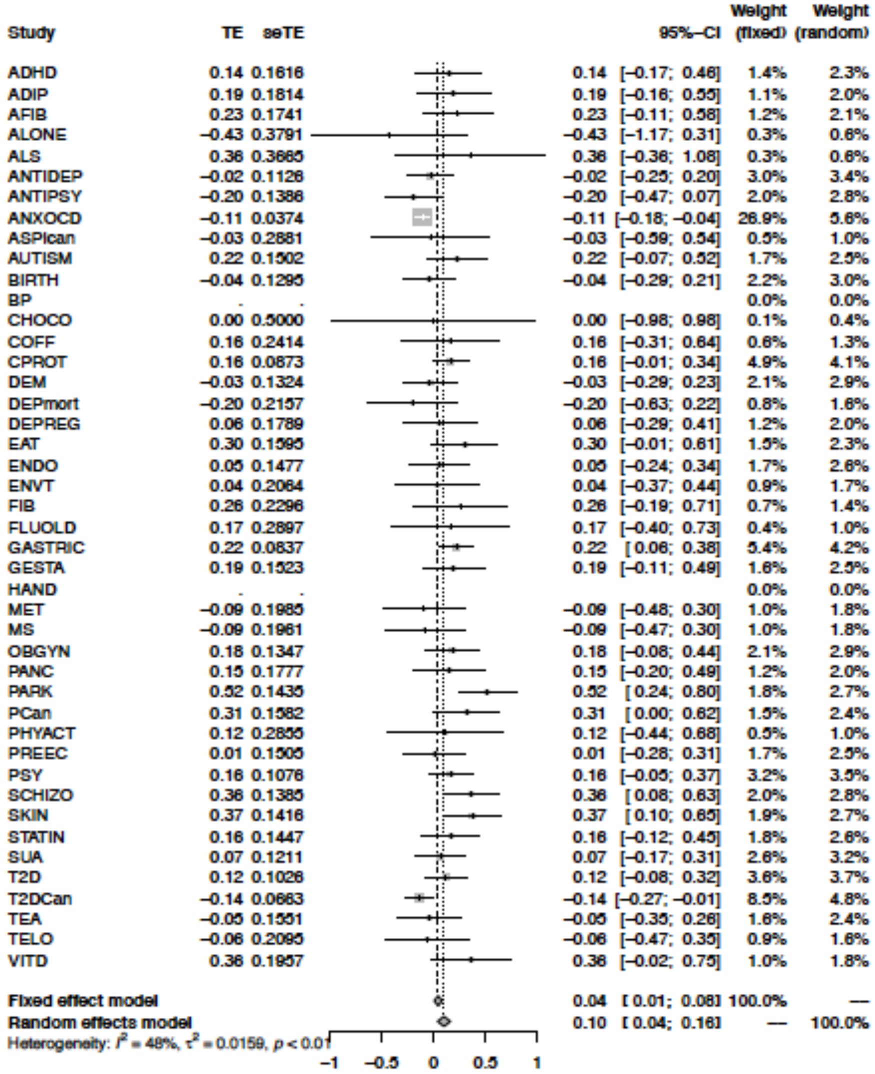

27) Prediction intervals and excess of significance bias

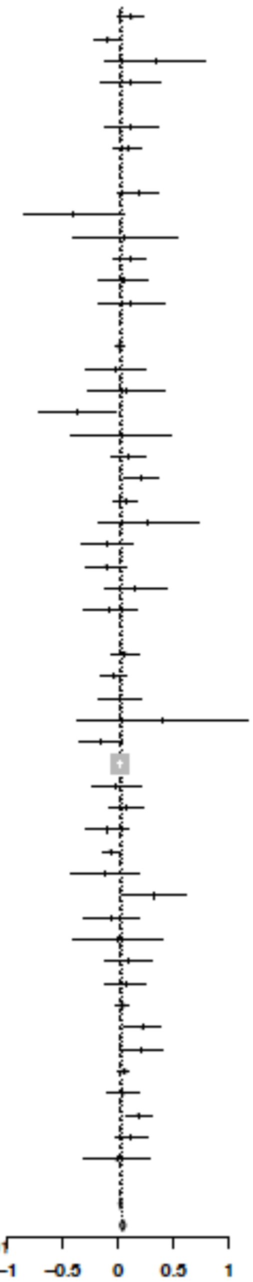

Study	TE	seTE		95%−CI	Weight (fixed)	Weight (random)
ADHD	0.11	0.0585	0.11	[0.00; 0.23]	0.7%	3.2%
ADIP	−0.09	0.0652	−0.09	[−0.22; 0.03]	0.6%	2.7%
ADOPSY	0.33	0.2268	0.33	[−0.11; 0.78]	0.0%	0.3%
AFIB	0.12	0.1341	0.12	[−0.15; 0.38]	0.1%	0.8%
ALONE	.	.			0.0%	0.0%
ALS	0.12	0.1238	0.12	[−0.12; 0.36]	0.2%	0.9%
ANTIDEP	0.08	0.0674	0.08	[−0.05; 0.22]	0.5%	2.6%
ANTIPSY	.	.			0.0%	0.0%
ANXOCD	0.18	0.0984	0.18	[−0.01; 0.37]	0.3%	1.4%
ASPI	−0.40	0.2319	−0.40	[−0.85; 0.05]	0.0%	0.3%
ASPIcan	0.06	0.2431	0.06	[−0.41; 0.54]	0.0%	0.3%
AUTISM	0.10	0.0725	0.10	[−0.04; 0.25]	0.4%	2.3%
BIRTH	0.05	0.1092	0.05	[−0.17; 0.26]	0.2%	1.2%
BP	0.12	0.1516	0.12	[−0.18; 0.41]	0.1%	0.6%
CHOCO	.	.			0.0%	0.0%
CKDmort	0.02	0.0189	0.02	[−0.02; 0.05]	6.6%	8.6%
COFF	−0.02	0.1372	−0.02	[−0.29; 0.25]	0.1%	0.8%
COLO	0.08	0.1781	0.08	[−0.27; 0.43]	0.1%	0.5%
COLO2	−0.36	0.1734	−0.36	[−0.70; −0.02]	0.1%	0.5%
COPD	0.03	0.2273	0.03	[−0.42; 0.47]	0.0%	0.3%
CPROT	0.09	0.0811	0.09	[−0.07; 0.25]	0.4%	1.9%
DEM	0.21	0.0797	0.21	[0.05; 0.36]	0.4%	2.0%
DEP	0.06	0.0516	0.06	[−0.04; 0.17]	0.9%	3.8%
DEPmort	0.27	0.2314	0.27	[−0.18; 0.73]	0.0%	0.3%
EAT	−0.10	0.1182	−0.10	[−0.34; 0.13]	0.2%	1.0%
ENDO	−0.11	0.0905	−0.11	[−0.28; 0.07]	0.3%	1.6%
ENVT	0.15	0.1418	0.15	[−0.13; 0.43]	0.1%	0.7%
FIB	−0.07	0.1197	−0.07	[−0.31; 0.16]	0.2%	1.0%
FISH	.	.			0.0%	0.0%
FLUOLD	0.06	0.0638	0.06	[−0.07; 0.18]	0.6%	2.8%
GASTRIC	−0.04	0.0612	−0.04	[−0.16; 0.08]	0.6%	3.0%
GESTA	0.02	0.0974	0.02	[−0.17; 0.21]	0.2%	1.4%
HAND	0.40	0.3919	0.40	[−0.37; 1.17]	0.0%	0.1%
HIV	−0.16	0.0996	−0.16	[−0.36; 0.03]	0.2%	1.4%
MENT	0.02	0.0057	0.02	[0.00; 0.03]	72.8%	10.6%
MET	−0.02	0.1145	−0.02	[−0.24; 0.21]	0.2%	1.1%
MG	0.07	0.0801	0.07	[−0.09; 0.23]	0.4%	2.0%
MS	−0.10	0.0970	−0.10	[−0.29; 0.09]	0.3%	1.4%
OBGYN	−0.06	0.0367	−0.06	[−0.14; 0.01]	1.8%	5.5%
PANC	−0.11	0.1544	−0.11	[−0.42; 0.19]	0.1%	0.6%
PARK	0.32	0.1418	0.32	[0.04; 0.60]	0.1%	0.7%
PCan	−0.06	0.1282	−0.06	[−0.31; 0.19]	0.1%	0.9%
PHYACT	0.00	0.2020	0.00	[−0.40; 0.40]	0.1%	0.4%
PREEC	0.10	0.1059	0.10	[−0.11; 0.30]	0.2%	1.2%
PSY	0.07	0.0949	0.07	[−0.12; 0.26]	0.3%	1.5%
RHEU	0.04	0.0249	0.04	[−0.01; 0.08]	3.8%	7.5%
SCHIZO	0.22	0.0805	0.22	[0.06; 0.38]	0.4%	1.9%
SKIN	0.21	0.0976	0.21	[0.02; 0.40]	0.2%	1.4%
STATIN	0.05	0.0258	0.05	[0.00; 0.10]	3.5%	7.4%
SUA	0.04	0.0756	0.04	[−0.11; 0.19]	0.4%	2.2%
T2D	0.19	0.0610	0.19	[0.07; 0.30]	0.6%	3.0%
T2DCan	0.12	0.0758	0.12	[−0.03; 0.27]	0.4%	2.1%
VITD	−0.01	0.1486	−0.01	[−0.30; 0.28]	0.1%	0.7%
**Fixed effect model**			0.02	[0.01; 0.03]	100.0%	—
**Random effects model**			0.04	[0.02; 0.07]	—	100.0%

Heterogeneity: $I^2 = 37\%$, $\tau^2 = 0.0014$, $p < 0.01$

28) Prediction intervals and I^2 > 50%

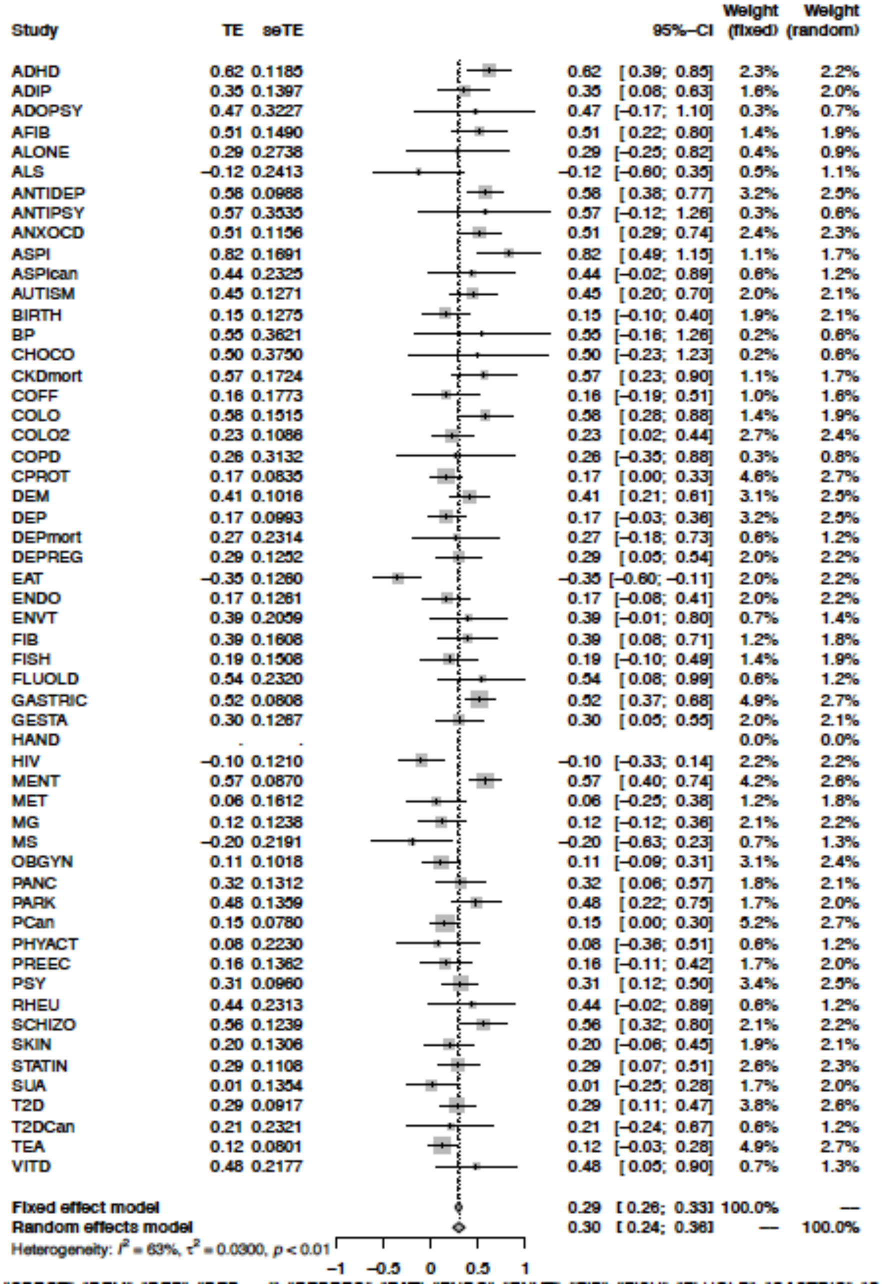

29) Prediction intervals and small study effects

Study	TE	seTE	95%-CI	Weight (fixed)	Weight (random)
ADHD	0.01	0.1140	0.01 [−0.22; 0.23]	0.7%	1.4%
ADIP	0.13	0.1057	0.13 [−0.07; 0.34]	0.8%	1.6%
ADOPSY	−0.07	0.3493	−0.07 [−0.75; 0.62]	0.1%	0.2%
AFIB	0.21	0.1093	0.21 [−0.01; 0.42]	0.8%	1.5%
ALONE	−0.92	0.3241	−0.92 [−1.56; −0.29]	0.1%	0.2%
ALS	0.25	0.1849	0.25 [−0.11; 0.61]	0.3%	0.6%
ANTIDEP	−0.04	0.0752	−0.04 [−0.19; 0.11]	1.7%	2.7%
ANTIPSY	0.18	0.1920	0.18 [−0.19; 0.56]	0.3%	0.6%
ANXOCD	0.03	0.0558	0.03 [−0.08; 0.13]	3.0%	4.0%
ASPI	.	.		0.0%	0.0%
ASPIcan	0.06	0.2431	0.06 [−0.41; 0.54]	0.2%	0.4%
AUTISM	−0.07	0.1263	−0.07 [−0.32; 0.17]	0.6%	1.2%
BIRTH	−0.07	0.1021	−0.07 [−0.27; 0.13]	0.9%	1.7%
BP	0.12	0.1516	0.12 [−0.18; 0.41]	0.4%	0.9%
CHOCO	0.50	0.3750	0.50 [−0.23; 1.23]	0.1%	0.2%
CKDmort	0.07	0.0890	0.07 [−0.10; 0.25]	1.2%	2.1%
COFF	−0.16	0.1774	−0.16 [−0.51; 0.19]	0.3%	0.6%
COLO	−0.03	0.1732	−0.03 [−0.37; 0.31]	0.3%	0.7%
COLO2	0.31	0.1848	0.31 [−0.05; 0.67]	0.3%	0.6%
COPD	−0.23	0.2226	−0.23 [−0.67; 0.20]	0.2%	0.4%
CPROT	0.05	0.0853	0.05 [−0.12; 0.21]	1.3%	2.3%
DEM	0.08	0.0866	0.08 [−0.09; 0.25]	1.3%	2.2%
DEP	0.07	0.0496	0.07 [−0.03; 0.16]	3.8%	4.5%
DEPmort	−0.19	0.1742	−0.19 [−0.53; 0.16]	0.3%	0.7%
DEPREG	−0.08	0.1126	−0.08 [−0.30; 0.14]	0.7%	1.5%
EAT	−0.01	0.0874	−0.01 [−0.18; 0.16]	1.2%	2.2%
ENDO	0.10	0.0722	0.10 [−0.04; 0.24]	1.8%	2.9%
ENVT	0.40	0.1487	0.40 [0.10; 0.69]	0.4%	0.9%
FIB	−0.11	0.1718	−0.11 [−0.45; 0.22]	0.3%	0.7%
FISH	0.40	0.2319	0.40 [−0.05; 0.85]	0.2%	0.4%
FLUOLD	0.14	0.0973	0.14 [−0.05; 0.33]	1.0%	1.9%
GASTRIC	0.05	0.1016	0.05 [−0.15; 0.25]	0.9%	1.7%
GESTA	0.15	0.0787	0.15 [0.00; 0.31]	1.5%	2.6%
HAND	0.00	0.3849	0.00 [−0.75; 0.75]	0.1%	0.1%
HIV	0.02	0.0669	0.02 [−0.11; 0.16]	2.1%	3.2%
MENT	0.02	0.0151	0.02 [−0.01; 0.05]	41.4%	8.4%
MET	−0.06	0.0977	−0.06 [−0.25; 0.13]	1.0%	1.8%
MS	−0.20	0.2008	−0.20 [−0.59; 0.19]	0.2%	0.5%
OBGYN	0.05	0.0598	0.05 [−0.07; 0.16]	2.6%	3.7%
PANC	0.06	0.0809	0.06 [−0.10; 0.22]	1.4%	2.5%
PARK	0.09	0.1350	0.09 [−0.17; 0.36]	0.5%	1.1%
PCan	0.19	0.0882	0.19 [0.02; 0.36]	1.2%	2.2%
PHYACT	−0.29	0.2397	−0.29 [−0.76; 0.18]	0.2%	0.4%
PREEC	0.01	0.0905	0.01 [−0.17; 0.19]	1.2%	2.1%
PSY	0.12	0.0684	0.12 [−0.01; 0.26]	2.0%	3.1%
RHEU	0.06	0.0400	0.06 [−0.02; 0.14]	5.9%	5.5%
SCHIZO	0.02	0.0706	0.02 [−0.12; 0.16]	1.9%	3.0%
SKIN	0.23	0.0692	0.23 [0.10; 0.37]	2.0%	3.1%
STATIN	0.07	0.0522	0.07 [−0.03; 0.17]	3.5%	4.3%
SUA	0.33	0.1150	0.33 [0.11; 0.56]	0.7%	1.4%
T2D	0.00	0.0551	0.00 [−0.11; 0.11]	3.1%	4.1%
T2DCan	−0.08	0.1056	−0.08 [−0.29; 0.13]	0.8%	1.6%
TEA	−0.17	0.1423	−0.17 [−0.45; 0.11]	0.5%	1.0%
VITD	0.26	0.1412	0.26 [−0.01; 0.54]	0.5%	1.0%
**Fixed effect model**			0.04 [0.02; 0.06]	100.0%	—
**Random effects model**			0.06 [0.03; 0.08]	—	100.0%

Heterogeneity: $I^2 = 28\%$, $\tau^2 = 0.0024$, $p = 0.03$

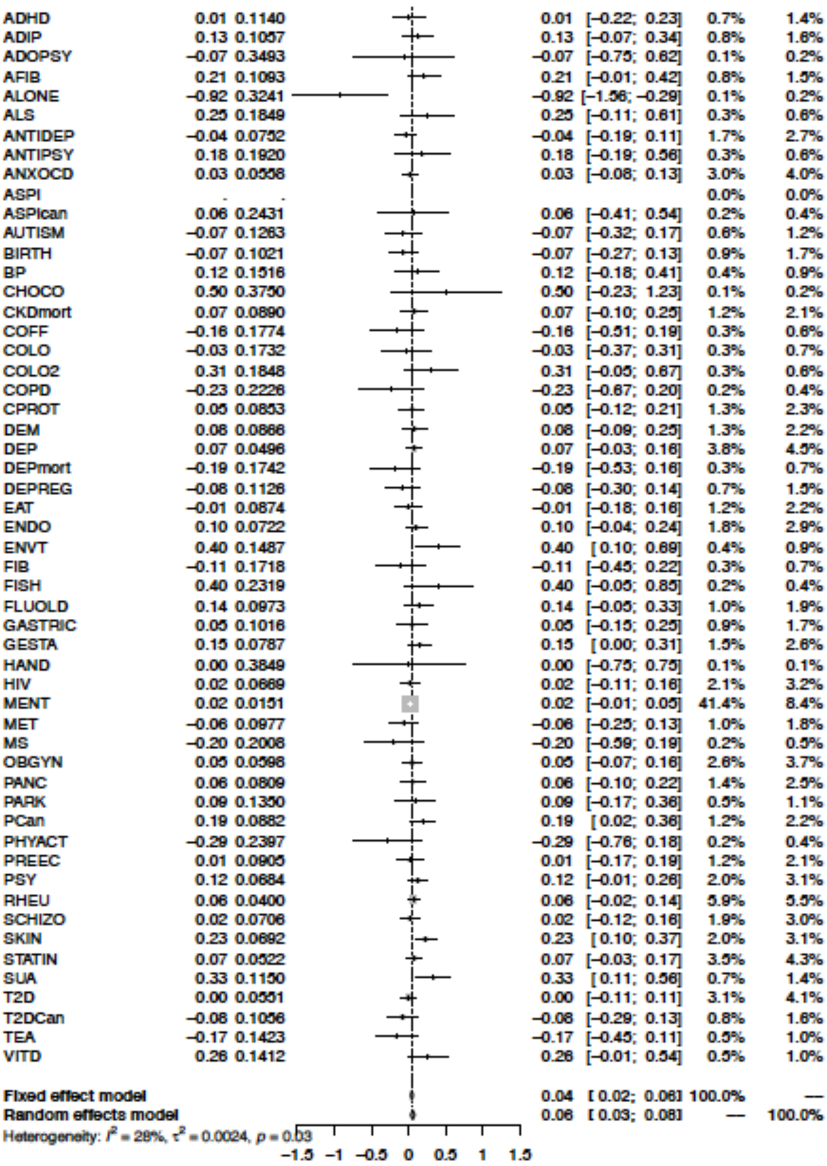

30) P-values < 10^{-6} and number of cases

Study	TE	seTE		95%-CI	Weight (fixed)	Weight (random)
ADHD	0.14	0.1257	0.14	[−0.11; 0.39]	0.5%	1.4%
ADIP	−0.02	0.1074	−0.02	[−0.23; 0.19]	0.7%	1.8%
ADOPSY	.	.			0.0%	0.0%
AFIB	0.12	0.1103	0.12	[−0.09; 0.34]	0.7%	1.7%
ALONE	.	.			0.0%	0.0%
ALS	.	.			0.0%	0.0%
ANTIDEP	−0.05	0.0578	−0.05	[−0.16; 0.06]	2.4%	4.1%
ANTIPSY	.	.			0.0%	0.0%
ANXOCD	0.22	0.1477	0.22	[−0.06; 0.51]	0.4%	1.0%
ASPI	.	.			0.0%	0.0%
AUTISM	0.04	0.0780	0.04	[−0.11; 0.19]	1.3%	2.9%
BIRTH	0.05	0.0842	0.05	[−0.12; 0.21]	1.1%	2.6%
BP	0.18	0.1920	0.18	[−0.19; 0.56]	0.2%	0.6%
CHOCO	.	.			0.0%	0.0%
CKDmort	0.29	0.1280	0.29	[0.04; 0.54]	0.5%	1.3%
COFF	.	.			0.0%	0.0%
COLO	0.04	0.1511	0.04	[−0.26; 0.34]	0.3%	1.0%
COLO2	−0.19	0.0971	−0.19	[−0.38; 0.00]	0.8%	2.1%
COPD	0.21	0.1939	0.21	[−0.17; 0.59]	0.2%	0.6%
CPROT	0.09	0.1024	0.09	[−0.11; 0.29]	0.8%	1.9%
DEM	0.12	0.0915	0.12	[−0.06; 0.30]	1.0%	2.3%
DEP	0.12	0.0987	0.12	[−0.08; 0.31]	0.8%	2.0%
DEPmort	0.41	0.2212	0.41	[−0.03; 0.84]	0.2%	0.5%
DEPREG	0.06	0.0349	0.06	[−0.01; 0.13]	6.5%	6.2%
EAT	0.05	0.1488	0.05	[−0.24; 0.34]	0.4%	1.0%
ENDO	−0.02	0.0962	−0.02	[−0.21; 0.17]	0.9%	2.1%
ENVT	−0.37	0.1473	−0.37	[−0.65; −0.08]	0.4%	1.0%
FIB	0.18	0.1109	0.18	[−0.04; 0.40]	0.6%	1.7%
FISH	.	.			0.0%	0.0%
FLUOLD	−0.02	0.1760	−0.02	[−0.36; 0.33]	0.3%	0.8%
GASTRIC	0.16	0.0901	0.16	[−0.02; 0.34]	1.0%	2.3%
GESTA	0.39	0.0956	0.39	[0.21; 0.58]	0.9%	2.1%
HAND	0.40	0.3919	0.40	[−0.37; 1.17]	0.1%	0.2%
HIV	0.07	0.1770	0.07	[−0.28; 0.41]	0.3%	0.8%
MENT	0.04	0.0244	0.04	[0.00; 0.09]	13.4%	7.3%
MET	0.30	0.1300	0.30	[0.05; 0.56]	0.5%	1.3%
MG	0.04	0.0549	0.04	[−0.07; 0.15]	2.6%	4.3%
MS	0.33	0.1816	0.33	[−0.03; 0.69]	0.2%	0.7%
OBGYN	0.15	0.0850	0.15	[−0.02; 0.32]	1.1%	2.5%
PANC	0.33	0.2094	0.33	[−0.08; 0.74]	0.2%	0.6%
PARK	0.18	0.1037	0.18	[−0.03; 0.38]	0.7%	1.9%
PCan	0.02	0.0141	0.02	[−0.01; 0.04]	39.9%	8.3%
PHYACT	0.24	0.1476	0.24	[−0.05; 0.52]	0.4%	1.0%
PREEC	0.37	0.1304	0.37	[0.11; 0.62]	0.5%	1.3%
PSY	−0.09	0.1103	−0.09	[−0.30; 0.13]	0.7%	1.7%
RHEU	−0.06	0.1724	−0.06	[−0.40; 0.28]	0.3%	0.8%
SCHIZO	0.06	0.1331	0.06	[−0.20; 0.32]	0.5%	1.2%
SKIN	0.08	0.0888	0.08	[−0.09; 0.26]	1.0%	2.4%
STATIN	−0.01	0.0548	−0.01	[−0.12; 0.10]	2.7%	4.3%
SUA	0.08	0.1381	0.08	[−0.19; 0.35]	0.4%	1.2%
T2D	0.08	0.0322	0.08	[0.02; 0.15]	7.7%	6.5%
T2DCan	0.04	0.0461	0.04	[−0.05; 0.13]	3.8%	5.1%
TEA	.	.			0.0%	0.0%
TELO	−0.01	0.1910	−0.01	[−0.39; 0.36]	0.2%	0.7%
VITD	−0.04	0.1776	−0.04	[−0.39; 0.31]	0.3%	0.7%
**Fixed effect model**			0.05	[0.03; 0.06]	100.0%	—
**Random effects model**			0.07	[0.04; 0.10]	—	100.0%

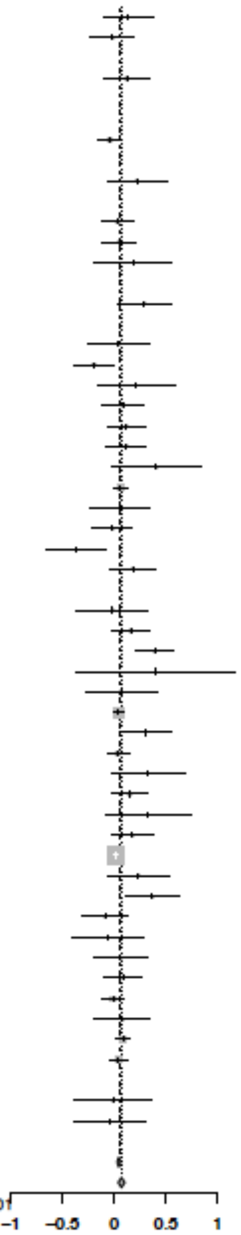

Heterogeneity: $I^2 = 40\%$, $\tau^2 = 0.0029$, $p < 0.01$

31) P-values < 10^{-6} and excess of significance bias

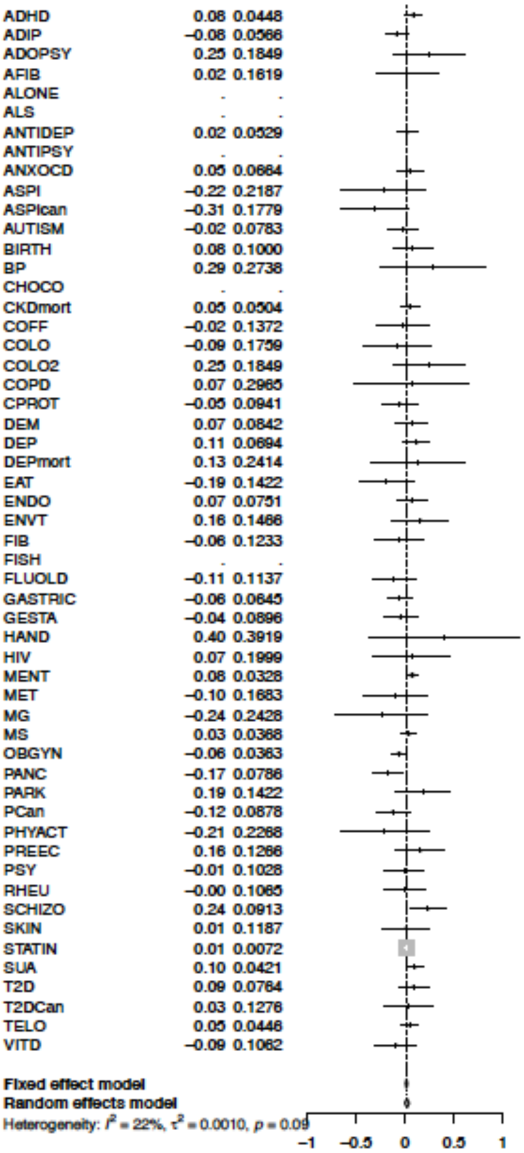

Study	TE	seTE		95%–CI	Weight (fixed)	Weight (random)
ADHD	0.08	0.0448	0.08	[0.00; 0.17]	1.8%	4.8%
ADIP	−0.08	0.0566	−0.08	[−0.19; 0.03]	1.1%	3.4%
ADOPSY	0.25	0.1849	0.25	[−0.11; 0.61]	0.1%	0.4%
AFIB	0.02	0.1619	0.02	[−0.30; 0.34]	0.1%	0.5%
ALONE	.	.			0.0%	0.0%
ALS	.	.			0.0%	0.0%
ANTIDEP	0.02	0.0529	0.02	[−0.08; 0.12]	1.3%	3.8%
ANTIPSY	.	.			0.0%	0.0%
ANXOCD	0.05	0.0664	0.05	[−0.08; 0.18]	0.8%	2.7%
ASPI	−0.22	0.2187	−0.22	[−0.64; 0.21]	0.1%	0.3%
ASPIcan	−0.31	0.1779	−0.31	[−0.66; 0.04]	0.1%	0.4%
AUTISM	−0.02	0.0783	−0.02	[−0.17; 0.14]	0.6%	2.0%
BIRTH	0.08	0.1000	0.08	[−0.12; 0.28]	0.4%	1.3%
BP	0.29	0.2738	0.29	[−0.25; 0.82]	0.0%	0.2%
CHOCO	.	.			0.0%	0.0%
CKDmort	0.05	0.0504	0.05	[−0.05; 0.15]	1.4%	4.1%
COFF	−0.02	0.1372	−0.02	[−0.29; 0.25]	0.2%	0.7%
COLO	−0.09	0.1759	−0.09	[−0.43; 0.26]	0.1%	0.4%
COLO2	0.25	0.1849	0.25	[−0.11; 0.61]	0.1%	0.4%
COPD	0.07	0.2965	0.07	[−0.51; 0.65]	0.0%	0.2%
CPROT	−0.05	0.0941	−0.05	[−0.24; 0.13]	0.4%	1.5%
DEM	0.07	0.0842	0.07	[−0.10; 0.23]	0.5%	1.8%
DEP	0.11	0.0694	0.11	[−0.02; 0.25]	0.8%	2.5%
DEPmort	0.13	0.2414	0.13	[−0.34; 0.60]	0.1%	0.2%
EAT	−0.19	0.1422	−0.19	[−0.47; 0.09]	0.2%	0.7%
ENDO	0.07	0.0751	0.07	[−0.08; 0.22]	0.6%	2.2%
ENVT	0.16	0.1466	0.16	[−0.13; 0.45]	0.2%	0.6%
FIB	−0.06	0.1233	−0.06	[−0.30; 0.18]	0.2%	0.9%
FISH	.	.			0.0%	0.0%
FLUOLD	−0.11	0.1137	−0.11	[−0.34; 0.11]	0.3%	1.0%
GASTRIC	−0.06	0.0645	−0.06	[−0.18; 0.07]	0.9%	2.8%
GESTA	−0.04	0.0896	−0.04	[−0.22; 0.13]	0.5%	1.6%
HAND	0.40	0.3919	0.40	[−0.37; 1.17]	0.0%	0.1%
HIV	0.07	0.1999	0.07	[−0.32; 0.46]	0.1%	0.3%
MENT	0.08	0.0328	0.08	[0.01; 0.14]	3.4%	7.0%
MET	−0.10	0.1683	−0.10	[−0.43; 0.23]	0.1%	0.5%
MG	−0.24	0.2428	−0.24	[−0.72; 0.23]	0.1%	0.2%
MS	0.03	0.0368	0.03	[−0.04; 0.11]	2.7%	6.1%
OBGYN	−0.06	0.0363	−0.06	[−0.14; 0.01]	2.8%	6.2%
PANC	−0.17	0.0786	−0.17	[−0.33; −0.02]	0.6%	2.0%
PARK	0.19	0.1422	0.19	[−0.09; 0.46]	0.2%	0.7%
PCan	−0.12	0.0878	−0.12	[−0.30; 0.05]	0.5%	1.6%
PHYACT	−0.21	0.2268	−0.21	[−0.65; 0.24]	0.1%	0.3%
PREEC	0.16	0.1266	0.16	[−0.09; 0.40]	0.2%	0.8%
PSY	−0.01	0.1028	−0.01	[−0.21; 0.20]	0.3%	1.2%
RHEU	−0.00	0.1065	−0.00	[−0.21; 0.21]	0.3%	1.2%
SCHIZO	0.24	0.0913	0.24	[0.06; 0.41]	0.4%	1.5%
SKIN	0.01	0.1187	0.01	[−0.22; 0.24]	0.3%	0.9%
STATIN	0.01	0.0072	0.01	[0.00; 0.03]	69.8%	13.8%
SUA	0.10	0.0421	0.10	[0.02; 0.18]	2.1%	5.2%
T2D	0.09	0.0764	0.09	[−0.06; 0.24]	0.6%	2.1%
T2DCan	0.03	0.1276	0.03	[−0.22; 0.28]	0.2%	0.8%
TELO	0.05	0.0446	0.05	[−0.04; 0.13]	1.8%	4.8%
VITD	−0.09	0.1062	−0.09	[−0.30; 0.12]	0.3%	1.2%
**Fixed effect model**			0.01	[0.00; 0.03]	100.0%	—
**Random effects model**			0.02	[0.00; 0.04]	—	100.0%

Heterogeneity: $I^2 = 22\%$, $\tau^2 = 0.0010$, $p = 0.09$

32) P-values < 10^{-6} and I^2 > 50%

Study	TE	seTE	95%–CI	Weight (fixed)	Weight (random)
ADHD	0.28	0.1340	0.28 [0.02; 0.55]	0.7%	1.9%
ADIP	−0.27	0.1369	−0.27 [−0.54; 0.00]	0.6%	1.8%
ADOPSY	−0.15	0.3153	−0.15 [−0.77; 0.46]	0.1%	0.4%
AFIB	0.20	0.1608	0.20 [−0.11; 0.52]	0.5%	1.4%
ALONE	−0.36	0.2716	−0.36 [−0.90; 0.17]	0.2%	0.6%
ALS	.	.		0.0%	0.0%
ANTIDEP	0.04	0.1024	0.04 [−0.16; 0.25]	1.2%	2.7%
ANTIPSY	0.33	0.2869	0.33 [−0.23; 0.90]	0.1%	0.5%
ANXOCD	0.17	0.0885	0.17 [0.00; 0.34]	1.5%	3.2%
ASPI	−0.18	0.1643	−0.18 [−0.50; 0.14]	0.4%	1.4%
ASPIcan	0.06	0.2431	0.06 [−0.41; 0.54]	0.2%	0.7%
AUTISM	0.17	0.1256	0.17 [−0.07; 0.42]	0.8%	2.0%
BIRTH	−0.11	0.1294	−0.11 [−0.36; 0.15]	0.7%	2.0%
BP	0.00	0.3849	0.00 [−0.75; 0.75]	0.1%	0.3%
CHOCO	.	.		0.0%	0.0%
CKDmort	0.11	0.1616	0.11 [−0.20; 0.43]	0.5%	1.4%
COFF	−0.05	0.1917	−0.05 [−0.43; 0.33]	0.3%	1.0%
COLO	0.19	0.1568	0.19 [−0.12; 0.50]	0.5%	1.5%
COLO2	0.10	0.1262	0.10 [−0.15; 0.35]	0.8%	2.0%
COPD	−0.02	0.1904	−0.02 [−0.40; 0.35]	0.3%	1.1%
CPROT	−0.01	0.0961	−0.01 [−0.20; 0.18]	1.3%	2.9%
DEM	0.24	0.1062	0.24 [0.04; 0.45]	1.1%	2.6%
DEP	0.04	0.1015	0.04 [−0.15; 0.24]	1.2%	2.7%
DEPmort	−0.12	0.2353	−0.12 [−0.58; 0.34]	0.2%	0.7%
DEPREG	0.11	0.0998	0.11 [−0.08; 0.31]	1.2%	2.8%
EAT	−0.00	0.1554	−0.00 [−0.31; 0.30]	0.5%	1.5%
ENDO	−0.28	0.1204	−0.28 [−0.51; −0.04]	0.8%	2.2%
ENVT	0.07	0.2097	0.07 [−0.34; 0.48]	0.3%	0.9%
FIB	0.28	0.1870	0.28 [−0.09; 0.64]	0.3%	1.1%
FISH	.	.		0.0%	0.0%
FLUOLD	0.23	0.1807	0.23 [−0.12; 0.59]	0.4%	1.2%
GASTRIC	0.34	0.0915	0.34 [0.16; 0.52]	1.5%	3.1%
GESTA	−0.24	0.1211	−0.24 [−0.48; −0.01]	0.8%	2.2%
HAND	.	.		0.0%	0.0%
HIV	−0.11	0.1506	−0.11 [−0.41; 0.18]	0.5%	1.6%
MENT	0.12	0.0631	0.12 [0.00; 0.25]	3.0%	4.5%
MET	−0.19	0.1712	−0.19 [−0.53; 0.15]	0.4%	1.3%
MG	−0.24	0.2301	−0.24 [−0.69; 0.22]	0.2%	0.8%
MS	0.14	0.1882	0.14 [−0.23; 0.51]	0.3%	1.1%
OBGYN	−0.17	0.0965	−0.17 [−0.36; 0.02]	1.3%	2.9%
PANC	−0.04	0.0732	−0.04 [−0.18; 0.10]	2.3%	3.9%
PARK	0.11	0.1494	0.11 [−0.18; 0.41]	0.5%	1.6%
PCan	0.02	0.0141	0.02 [−0.01; 0.04]	60.7%	7.2%
PHYACT	−0.13	0.2354	−0.13 [−0.59; 0.33]	0.2%	0.7%
PREEC	−0.19	0.1513	−0.19 [−0.48; 0.11]	0.5%	1.5%
PSY	−0.03	0.1019	−0.03 [−0.23; 0.17]	1.2%	2.7%
RHEU	0.00	0.1435	0.00 [−0.28; 0.29]	0.6%	1.7%
SCHIZO	0.38	0.1302	0.38 [0.13; 0.64]	0.7%	1.9%
SKIN	−0.15	0.1264	−0.15 [−0.39; 0.10]	0.8%	2.0%
STATIN	0.06	0.0607	0.06 [−0.05; 0.18]	3.3%	4.6%
SUA	−0.08	0.1322	−0.08 [−0.34; 0.18]	0.7%	1.9%
T2D	−0.00	0.0927	−0.00 [−0.18; 0.18]	1.4%	3.1%
T2DCan	0.05	0.2271	0.05 [−0.40; 0.49]	0.2%	0.8%
TEA	−0.10	0.0982	−0.10 [−0.29; 0.09]	1.3%	2.9%
TELO	0.25	0.2243	0.25 [−0.19; 0.69]	0.2%	0.8%
VITD	−0.11	0.1892	−0.11 [−0.48; 0.26]	0.3%	1.1%
**Fixed effect model**			0.02 [0.00; 0.04]	100.0%	—
**Random effects model**			0.03 [−0.02; 0.07]	—	100.0%

Heterogeneity: $I^2 = 38\%$, $\tau^2 = 0.0060$, $p < 0.01$

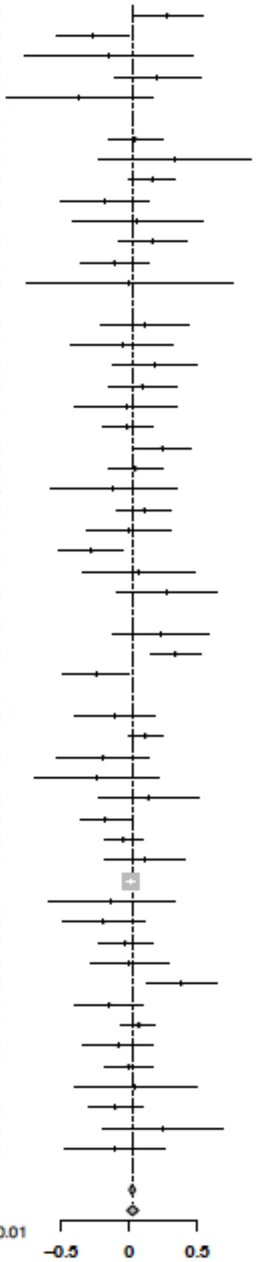

33) P-values < 10^{-6} and largest studies

Study	TE	seTE	95%-CI	Weight (fixed)	Weight (random)
ADHD	0.18	0.0873	0.18 [0.01; 0.35]	1.4%	2.5%
ADIP	0.24	0.0963	0.24 [0.05; 0.43]	1.1%	2.2%
AFIB	0.18	0.0972	0.18 [−0.01; 0.37]	1.1%	2.2%
ALONE	0.29	0.2738	0.29 [−0.25; 0.82]	0.1%	0.4%
ALS	.	.		0.0%	0.0%
ANTIDEP	0.12	0.0627	0.12 [−0.01; 0.24]	2.7%	3.7%
ANTIPSY	0.33	0.2869	0.33 [−0.23; 0.90]	0.1%	0.3%
ANXOCD	0.10	0.0523	0.10 [0.00; 0.20]	3.9%	4.3%
ASPIcan	0.44	0.2325	0.44 [−0.02; 0.89]	0.2%	0.5%
AUTISM	0.21	0.0842	0.21 [0.04; 0.37]	1.5%	2.7%
BIRTH	0.26	0.1126	0.26 [0.04; 0.48]	0.8%	1.8%
BP	.	.		0.0%	0.0%
CHOCO	.	.		0.0%	0.0%
COFF	−0.05	0.1917	−0.05 [−0.43; 0.33]	0.3%	0.7%
CPROT	0.19	0.0613	0.19 [0.07; 0.31]	2.8%	3.8%
DEM	0.21	0.0797	0.21 [0.05; 0.36]	1.7%	2.9%
DEPmort	0.23	0.1908	0.23 [−0.14; 0.61]	0.3%	0.7%
DEPREG	0.09	0.0469	0.09 [−0.01; 0.18]	4.8%	4.7%
EAT	0.28	0.1210	0.28 [0.04; 0.51]	0.7%	1.6%
ENDO	0.24	0.0856	0.24 [0.07; 0.40]	1.4%	2.6%
ENVT	0.23	0.1261	0.23 [−0.01; 0.48]	0.7%	1.5%
FIB	−0.14	0.2061	−0.14 [−0.54; 0.27]	0.2%	0.6%
FLUOLD	0.01	0.1047	0.01 [−0.20; 0.21]	1.0%	2.0%
GASTRIC	0.20	0.0772	0.20 [0.05; 0.35]	1.8%	3.0%
GESTA	0.25	0.0767	0.25 [0.10; 0.40]	1.8%	3.0%
HAND	.	.		0.0%	0.0%
MET	0.15	0.1402	0.15 [−0.13; 0.42]	0.5%	1.3%
MS	0.14	0.0808	0.14 [−0.02; 0.30]	1.6%	2.8%
OBGYN	0.14	0.0543	0.14 [0.04; 0.25]	3.6%	4.2%
PANC	0.06	0.0358	0.06 [−0.01; 0.13]	8.3%	5.5%
PARK	0.34	0.1404	0.34 [0.06; 0.61]	0.5%	1.3%
PCan	0.07	0.0479	0.07 [−0.03; 0.16]	4.6%	4.6%
PHYACT	0.15	0.2194	0.15 [−0.28; 0.58]	0.2%	0.6%
PREEC	0.53	0.1274	0.53 [0.28; 0.78]	0.7%	1.5%
PSY	0.26	0.0988	0.26 [0.07; 0.45]	1.1%	2.2%
SCHIZO	0.09	0.0681	0.09 [−0.04; 0.23]	2.3%	3.4%
SKIN	0.10	0.0864	0.10 [−0.07; 0.27]	1.4%	2.6%
STATIN	0.03	0.0180	0.03 [0.00; 0.07]	32.8%	6.6%
SUA	0.12	0.0484	0.12 [0.03; 0.21]	4.5%	4.6%
T2D	0.20	0.0524	0.20 [0.10; 0.30]	3.9%	4.3%
T2DCan	0.14	0.0889	0.14 [−0.03; 0.31]	1.3%	2.5%
TEA	0.22	0.1908	0.22 [−0.15; 0.60]	0.3%	0.7%
TELO	0.17	0.0980	0.17 [−0.03; 0.36]	1.1%	2.2%
VITD	0.46	0.1356	0.46 [0.20; 0.73]	0.6%	1.3%
**Fixed effect model**			0.11 [0.09; 0.13]	100.0%	—
**Random effects model**			0.16 [0.12; 0.19]	—	100.0%

Heterogeneity: $I^2 = 47\%$, $\tau^2 = 0.0042$, $p < 0.01$

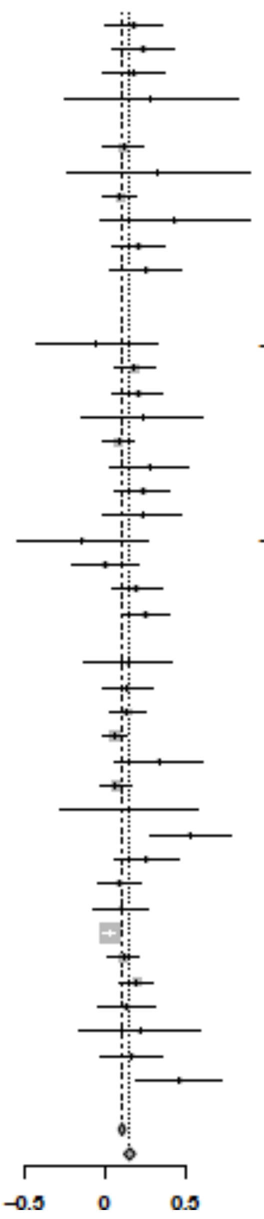

34) P-values < 10^{-6} and prediction intervals

Study	TE	seTE	95%-CI	Weight (fixed)	Weight (random)
ADHD	0.64	0.1229	0.64 [0.40; 0.88]	1.3%	2.1%
ADIP	0.31	0.1402	0.31 [0.04; 0.59]	1.0%	2.0%
ADOPSY	0.47	0.3227	0.47 [−0.17; 1.10]	0.2%	1.1%
AFIB	0.43	0.1426	0.43 [0.15; 0.71]	1.0%	2.0%
ALONE	0.62	0.3175	0.62 [−0.01; 1.24]	0.2%	1.1%
ALS	.	.		0.0%	0.0%
ANTIDEP	0.40	0.1150	0.40 [0.18; 0.63]	1.5%	2.2%
ANTIPSY	0.67	0.2869	0.67 [0.10; 1.23]	0.2%	1.3%
ANXOCD	0.73	0.1029	0.73 [0.53; 0.93]	1.9%	2.2%
ASPI	0.15	0.1489	0.15 [−0.14; 0.45]	0.9%	2.0%
ASPIcan	0.40	0.3481	0.40 [−0.28; 1.08]	0.2%	1.0%
AUTISM	0.73	0.1022	0.73 [0.53; 0.93]	1.9%	2.2%
BIRTH	0.29	0.1380	0.29 [0.02; 0.56]	1.1%	2.0%
BP	0.55	0.3621	0.55 [−0.16; 1.26]	0.2%	1.0%
CHOCO	.	.		0.0%	0.0%
CKDmort	0.35	0.1445	0.35 [0.07; 0.64]	1.0%	2.0%
COFF	0.43	0.2388	0.43 [−0.03; 0.90]	0.4%	1.5%
COLO	0.31	0.1607	0.31 [−0.01; 0.62]	0.8%	1.9%
COLO2	0.04	0.0269	0.04 [−0.02; 0.09]	27.9%	2.5%
COPD	0.41	0.1983	0.41 [0.02; 0.80]	0.5%	1.7%
CPROT	0.66	0.0816	0.66 [0.50; 0.82]	3.0%	2.3%
DEM	0.69	0.1097	0.69 [0.47; 0.90]	1.7%	2.2%
DEP	0.68	0.0770	0.68 [0.52; 0.83]	3.4%	2.3%
DEPmort	−0.06	0.1999	−0.06 [−0.45; 0.33]	0.5%	1.7%
DEPREG	0.65	0.1549	0.65 [0.34; 0.95]	0.8%	1.9%
EAT	0.29	0.1529	0.29 [−0.01; 0.59]	0.9%	2.0%
ENDO	0.33	0.1407	0.33 [0.05; 0.60]	1.0%	2.0%
ENVT	0.61	0.1700	0.61 [0.28; 0.95]	0.7%	1.9%
FIB	0.87	0.1257	0.87 [0.62; 1.12]	1.3%	2.1%
FISH	.	.		0.0%	0.0%
FLUOLD	0.54	0.2320	0.54 [0.08; 0.99]	0.4%	1.5%
GASTRIC	0.65	0.0798	0.65 [0.49; 0.80]	3.2%	2.3%
GESTA	0.37	0.1367	0.37 [0.10; 0.63]	1.1%	2.0%
HAND	0.40	0.2771	0.40 [−0.14; 0.94]	0.3%	1.3%
HIV	0.54	0.1525	0.54 [0.24; 0.84]	0.9%	2.0%
MENT	0.17	0.0501	0.17 [0.08; 0.27]	8.1%	2.4%
MET	0.51	0.2300	0.51 [0.06; 0.96]	0.4%	1.5%
MG	−0.12	0.0877	−0.12 [−0.30; 0.05]	2.6%	2.3%
MS	0.30	0.1847	0.30 [−0.06; 0.66]	0.6%	1.8%
OBGYN	0.64	0.0785	0.64 [0.49; 0.79]	3.3%	2.3%
PANC	0.41	0.1900	0.41 [0.03; 0.78]	0.6%	1.7%
PARK	0.38	0.1699	0.38 [0.05; 0.71]	0.7%	1.9%
PCan	0.22	0.1373	0.22 [−0.05; 0.49]	1.1%	2.0%
PHYACT	0.80	0.1876	0.80 [0.43; 1.17]	0.6%	1.8%
PREEC	0.45	0.1492	0.45 [0.16; 0.74]	0.9%	2.0%
PSY	0.62	0.0947	0.62 [0.43; 0.81]	2.3%	2.3%
RHEU	0.27	0.1316	0.27 [0.01; 0.52]	1.2%	2.1%
SCHIZO	0.79	0.0878	0.79 [0.62; 0.96]	2.6%	2.3%
SKIN	0.53	0.1169	0.53 [0.30; 0.76]	1.5%	2.1%
STATIN	0.18	0.1344	0.18 [−0.08; 0.45]	1.1%	2.1%
SUA	0.47	0.1284	0.47 [0.22; 0.73]	1.2%	2.1%
T2D	0.62	0.0744	0.62 [0.48; 0.77]	3.7%	2.3%
T2DCan	0.74	0.1703	0.74 [0.40; 1.07]	0.7%	1.9%
TEA	−0.08	0.0606	−0.08 [−0.20; 0.04]	5.5%	2.4%
VITD	0.13	0.2564	0.13 [−0.38; 0.63]	0.3%	1.4%
**Fixed effect model**			0.32 [0.29; 0.35]	100.0%	—
**Random effects model**			0.44 [0.35; 0.53]	—	100.0%

Heterogeneity: $I^2 = 88\%$, $\tau^2 = 0.0840$, $p < 0.01$

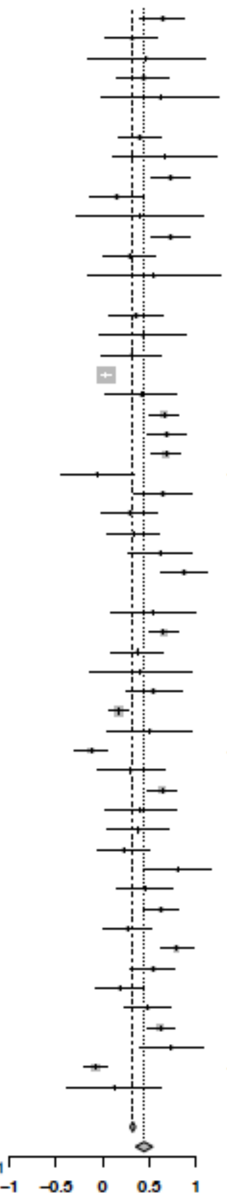

35) P-values < 10^{-6} and p-values < 0.001

Study	TE	seTE	95%–CI	Weight (fixed)	Weight (random)
ADHD	0.40	0.1013	0.40 [0.20; 0.60]	2.2%	2.4%
ADIP	0.34	0.1063	0.34 [0.13; 0.54]	2.0%	2.3%
ADOPSY	0.40	0.2263	0.40 [−0.04; 0.84]	0.4%	0.8%
AFIB	0.48	0.1280	0.48 [0.23; 0.73]	1.4%	1.9%
ALONE	0.29	0.2738	0.29 [−0.25; 0.82]	0.3%	0.6%
ALS	.	.		0.0%	0.0%
ANTIDEP	0.31	0.0741	0.31 [0.17; 0.46]	4.0%	3.1%
ANTIPSY	.	.		0.0%	0.0%
ANXOCD	0.44	0.0848	0.44 [0.27; 0.60]	3.1%	2.8%
ASPI	0.62	0.3346	0.62 [−0.04; 1.28]	0.2%	0.4%
ASPIcan	0.57	0.2499	0.57 [0.08; 1.06]	0.4%	0.7%
AUTISM	0.47	0.0982	0.47 [0.27; 0.66]	2.3%	2.5%
BIRTH	0.55	0.1018	0.55 [0.35; 0.75]	2.1%	2.4%
BP	0.40	0.2771	0.40 [−0.14; 0.94]	0.3%	0.6%
CHOCO	.	.		0.0%	0.0%
CKDmort	0.59	0.1259	0.59 [0.34; 0.83]	1.4%	1.9%
COFF	0.45	0.1723	0.45 [0.11; 0.79]	0.7%	1.3%
COLO	0.75	0.1147	0.75 [0.52; 0.97]	1.7%	2.1%
COLO2	0.53	0.1919	0.53 [0.16; 0.91]	0.6%	1.1%
COPD	0.35	0.2055	0.35 [−0.05; 0.75]	0.5%	1.0%
CPROT	0.38	0.0685	0.38 [0.24; 0.51]	4.7%	3.2%
DEM	0.48	0.0982	0.48 [0.28; 0.67]	2.3%	2.5%
DEP	0.45	0.0712	0.45 [0.31; 0.59]	4.4%	3.2%
DEPmort	0.66	0.1685	0.66 [0.33; 0.99]	0.8%	1.3%
DEPREG	0.27	0.1087	0.27 [0.05; 0.48]	1.9%	2.2%
EAT	0.50	0.1119	0.50 [0.28; 0.72]	1.8%	2.2%
ENDO	0.56	0.0961	0.56 [0.37; 0.75]	2.4%	2.5%
ENVT	0.56	0.1553	0.56 [0.26; 0.86]	0.9%	1.5%
FIB	0.25	0.1307	0.25 [−0.01; 0.51]	1.3%	1.8%
FISH	.	.		0.0%	0.0%
FLUOLD	0.35	0.1318	0.35 [0.09; 0.61]	1.3%	1.8%
GASTRIC	0.45	0.0730	0.45 [0.30; 0.59]	4.2%	3.1%
GESTA	0.45	0.1000	0.45 [0.25; 0.64]	2.2%	2.4%
HAND	0.57	0.3535	0.57 [−0.12; 1.26]	0.2%	0.4%
HIV	0.21	0.0796	0.21 [0.05; 0.37]	3.5%	2.9%
MENT	.	.		0.0%	0.0%
MET	0.47	0.1586	0.47 [0.15; 0.78]	0.9%	1.4%
MG	0.61	0.3395	0.61 [−0.06; 1.27]	0.2%	0.4%
MS	0.47	0.1586	0.47 [0.15; 0.78]	0.9%	1.4%
OBGYN	0.47	0.0725	0.47 [0.33; 0.61]	4.2%	3.1%
PANC	0.15	0.0745	0.15 [0.01; 0.30]	4.0%	3.1%
PARK	0.47	0.1204	0.47 [0.23; 0.70]	1.5%	2.0%
PCan	0.30	0.1684	0.30 [−0.03; 0.63]	0.8%	1.3%
PHYACT	0.43	0.1940	0.43 [0.05; 0.81]	0.6%	1.1%
PREEC	0.56	0.1154	0.56 [0.33; 0.79]	1.7%	2.1%
PSY	0.65	0.0790	0.65 [0.50; 0.81]	3.6%	3.0%
RHEU	0.54	0.1254	0.54 [0.30; 0.79]	1.4%	1.9%
SCHIZO	0.43	0.0892	0.43 [0.26; 0.61]	2.8%	2.7%
SKIN	0.54	0.0946	0.54 [0.36; 0.73]	2.5%	2.6%
STATIN	0.11	0.0521	0.11 [0.00; 0.21]	8.2%	3.7%
SUA	0.32	0.0832	0.32 [0.16; 0.49]	3.2%	2.8%
T2D	0.49	0.0664	0.49 [0.35; 0.62]	5.0%	3.3%
T2DCan	0.52	0.1633	0.52 [0.20; 0.84]	0.8%	1.4%
TEA	0.46	0.3052	0.46 [−0.14; 1.06]	0.2%	0.5%
TELO	0.50	0.1723	0.50 [0.16; 0.84]	0.7%	1.3%
VITD	0.37	0.1254	0.37 [0.12; 0.61]	1.4%	1.9%
**Fixed effect model**			0.41 [0.38; 0.44]	100.0%	—
**Random effects model**			0.43 [0.39; 0.48]	—	100.0%

Heterogeneity: $I^2 = 50\%$, $\tau^2 = 0.0114$, $p < 0.01$

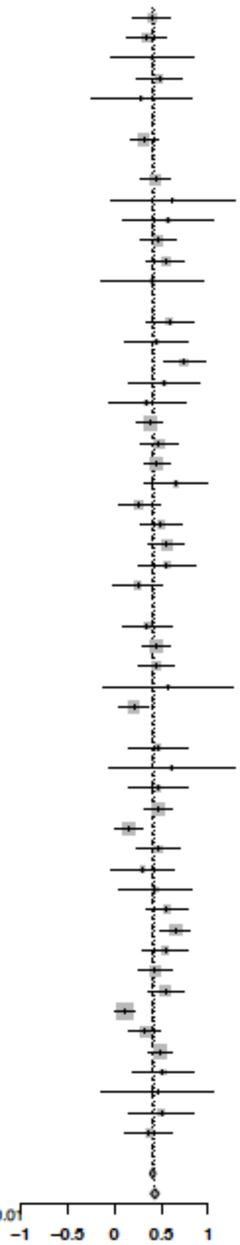

36) P-values < 10^{-6} and small study effects

Study	TE	seTE	95%-CI	Weight (fixed)	Weight (random)
ADHD	−0.05	0.1064	−0.05 [−0.26; 0.16]	1.2%	1.9%
ADIP	−0.05	0.1253	−0.05 [−0.30; 0.19]	0.9%	1.5%
ADOPSY	−0.07	0.3493	−0.07 [−0.75; 0.62]	0.1%	0.2%
AFIB	0.19	0.1478	0.19 [−0.10; 0.48]	0.6%	1.1%
ALONE	−0.67	0.2485	−0.67 [−1.15; −0.18]	0.2%	0.4%
ALS	.	.		0.0%	0.0%
ANTIDEP	−0.10	0.0695	−0.10 [−0.24; 0.03]	2.8%	3.5%
ANTIPSY	−0.33	0.2889	−0.33 [−0.90; 0.23]	0.2%	0.3%
ANXOCD	0.03	0.0558	0.03 [−0.08; 0.13]	4.3%	4.4%
ASPI	.	.		0.0%	0.0%
ASPIcan	0.06	0.2431	0.06 [−0.41; 0.54]	0.2%	0.4%
AUTISM	−0.20	0.1248	−0.20 [−0.44; 0.05]	0.9%	1.5%
BIRTH	−0.29	0.1132	−0.29 [−0.52; −0.07]	1.0%	1.7%
BP	0.18	0.1920	0.18 [−0.19; 0.56]	0.4%	0.7%
CHOCO	.	.		0.0%	0.0%
CKDmort	−0.27	0.1442	−0.27 [−0.56; 0.01]	0.6%	1.2%
COFF	−0.35	0.2106	−0.35 [−0.77; 0.06]	0.3%	0.6%
COLO	−0.42	0.1516	−0.42 [−0.72; −0.13]	0.6%	1.1%
COLO2	0.01	0.1015	0.01 [−0.19; 0.21]	1.3%	2.1%
COPD	−0.10	0.2715	−0.10 [−0.63; 0.44]	0.2%	0.4%
CPROT	−0.09	0.0962	−0.09 [−0.28; 0.10]	1.4%	2.2%
DEM	0.01	0.0883	0.01 [−0.17; 0.18]	1.7%	2.5%
DEP	0.06	0.0669	0.06 [−0.08; 0.19]	3.0%	3.6%
DEPmort	−0.12	0.2191	−0.12 [−0.55; 0.31]	0.3%	0.5%
DEPREG	−0.07	0.0900	−0.07 [−0.25; 0.11]	1.7%	2.5%
EAT	−0.07	0.1179	−0.07 [−0.30; 0.16]	1.0%	1.6%
ENDO	0.04	0.0806	0.04 [−0.11; 0.20]	2.1%	2.9%
ENVT	0.16	0.1893	0.16 [−0.21; 0.53]	0.4%	0.7%
FIB	−0.26	0.1964	−0.26 [−0.64; 0.13]	0.3%	0.7%
FISH	.	.		0.0%	0.0%
FLUOLD	−0.01	0.1486	−0.01 [−0.30; 0.28]	0.6%	1.1%
GASTRIC	0.09	0.1018	0.09 [−0.11; 0.29]	1.3%	2.0%
GESTA	0.03	0.0724	0.03 [−0.11; 0.17]	2.6%	3.3%
HAND	0.67	0.2889	0.67 [0.10; 1.23]	0.2%	0.3%
HIV	−0.01	0.1088	−0.01 [−0.22; 0.20]	1.1%	1.8%
MENT	−0.06	0.0509	−0.06 [−0.16; 0.04]	5.2%	4.8%
MET	−0.38	0.1917	−0.38 [−0.76; −0.01]	0.4%	0.7%
MS	−0.09	0.1413	−0.09 [−0.37; 0.18]	0.7%	1.2%
OBGYN	0.03	0.0651	0.03 [−0.10; 0.16]	3.2%	3.7%
PANC	−0.10	0.0881	−0.10 [−0.27; 0.08]	1.7%	2.5%
PARK	0.06	0.1306	0.06 [−0.20; 0.31]	0.8%	1.4%
PCan	0.03	0.0215	0.03 [−0.02; 0.07]	29.0%	7.6%
PHYACT	−0.50	0.2669	−0.50 [−1.02; 0.02]	0.2%	0.4%
PREEC	−0.16	0.1224	−0.16 [−0.40; 0.08]	0.9%	1.5%
PSY	0.00	0.0796	0.00 [−0.15; 0.16]	2.1%	2.9%
RHEU	−0.09	0.1356	−0.09 [−0.36; 0.17]	0.7%	1.3%
SCHIZO	0.02	0.0799	0.02 [−0.14; 0.18]	2.1%	2.9%
SKIN	−0.12	0.1000	−0.12 [−0.31; 0.08]	1.3%	2.1%
STATIN	−0.02	0.0359	−0.02 [−0.09; 0.05]	10.4%	6.2%
SUA	−0.01	0.1147	−0.01 [−0.23; 0.22]	1.0%	1.7%
T2D	−0.05	0.0669	−0.05 [−0.18; 0.09]	3.0%	3.6%
T2DCan	−0.21	0.1486	−0.21 [−0.50; 0.08]	0.6%	1.1%
TEA	−0.10	0.0982	−0.10 [−0.29; 0.09]	1.4%	2.2%
TELO	−0.09	0.1362	−0.09 [−0.36; 0.18]	0.7%	1.3%
VITD	0.19	0.0993	0.19 [0.00; 0.39]	1.4%	2.1%
Fixed effect model			−0.02 [−0.04; 0.01]	100.0%	—
Random effects model			−0.04 [−0.07; 0.00]	—	100.0%

Heterogeneity: $I^2 = 30\%$, $\tau^2 = 0.0032$, $p = 0.03$

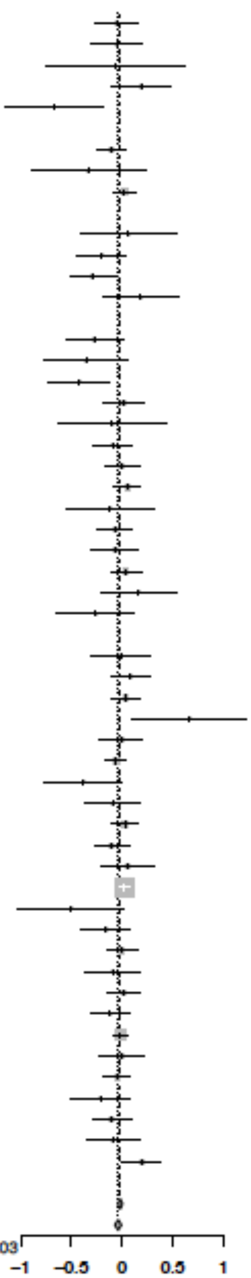

37) P-values < 0.001 and number of cases

Study	TE	seTE		95%-CI	Weight (fixed)	Weight (random)
ADHD	0.29	0.1449	0.29	[0.01; 0.58]	0.9%	1.8%
ADIP	0.33	0.1845	0.33	[−0.03; 0.69]	0.6%	1.2%
ADOPSY	.	.			0.0%	0.0%
AFIB	0.21	0.1882	0.21	[−0.16; 0.58]	0.5%	1.2%
ALONE	.	.			0.0%	0.0%
ALS	−0.15	0.3153	−0.15	[−0.77; 0.46]	0.2%	0.5%
ANTIDEP	−0.07	0.0912	−0.07	[−0.25; 0.11]	2.3%	3.0%
ANTIPSY	.	.			0.0%	0.0%
ANXOCD	0.12	0.0924	0.12	[−0.06; 0.30]	2.2%	2.9%
ASPI	.	.			0.0%	0.0%
AUTISM	−0.15	0.1015	−0.15	[−0.35; 0.05]	1.8%	2.7%
BIRTH	−0.04	0.1235	−0.04	[−0.28; 0.20]	1.2%	2.2%
BP	0.57	0.3535	0.57	[−0.12; 1.26]	0.2%	0.4%
CHOCO	.	.			0.0%	0.0%
CKDmort	0.67	0.1707	0.67	[0.33; 1.00]	0.6%	1.4%
COFF	.	.			0.0%	0.0%
COLO	0.06	0.1247	0.06	[−0.18; 0.30]	1.2%	2.1%
COLO2	0.21	0.1806	0.21	[−0.14; 0.57]	0.6%	1.3%
COPD	−0.14	0.1004	−0.14	[−0.33; 0.06]	1.9%	2.7%
CPROT	0.02	0.0854	0.02	[−0.15; 0.19]	2.6%	3.1%
DEM	0.14	0.1299	0.14	[−0.12; 0.39]	1.1%	2.0%
DEP	0.32	0.1271	0.32	[0.07; 0.57]	1.2%	2.1%
DEPmort	0.30	0.2239	0.30	[−0.14; 0.74]	0.4%	0.9%
DEPREG	0.08	0.1275	0.08	[−0.17; 0.33]	1.2%	2.1%
EAT	0.14	0.0944	0.14	[−0.04; 0.33]	2.1%	2.9%
ENDO	0.21	0.1282	0.21	[−0.04; 0.46]	1.2%	2.1%
ENVT	−0.32	0.1240	−0.32	[−0.57; −0.08]	1.2%	2.2%
FIB	0.12	0.2566	0.12	[−0.39; 0.62]	0.3%	0.7%
FISH	0.07	0.0801	0.07	[−0.09; 0.23]	3.0%	3.3%
FLUOLD	0.11	0.2350	0.11	[−0.35; 0.57]	0.3%	0.8%
GASTRIC	0.00	0.0569	0.00	[−0.11; 0.12]	5.8%	4.1%
GESTA	0.43	0.1275	0.43	[0.18; 0.68]	1.2%	2.1%
HAND	.	.			0.0%	0.0%
HIV	0.16	0.1384	0.16	[−0.11; 0.43]	1.0%	1.9%
MENT	0.04	0.0244	0.04	[0.00; 0.09]	31.8%	5.1%
MET	0.40	0.1840	0.40	[0.04; 0.76]	0.6%	1.2%
MG	0.09	0.1037	0.09	[−0.11; 0.29]	1.8%	2.6%
MS	0.27	0.2061	0.27	[−0.14; 0.67]	0.4%	1.0%
OBGYN	0.28	0.1034	0.28	[0.08; 0.49]	1.8%	2.6%
PANC	−0.02	0.0973	−0.02	[−0.21; 0.17]	2.0%	2.8%
PARK	0.18	0.1629	0.18	[−0.14; 0.50]	0.7%	1.5%
PCan	0.09	0.0512	0.09	[−0.01; 0.19]	7.2%	4.3%
PHYACT	0.65	0.2140	0.65	[0.23; 1.07]	0.4%	1.0%
PREEC	0.36	0.1513	0.36	[0.07; 0.66]	0.8%	1.7%
PSY	0.03	0.0978	0.03	[−0.17; 0.22]	2.0%	2.8%
RHEU	−0.19	0.1845	−0.19	[−0.56; 0.17]	0.6%	1.2%
SCHIZO	0.09	0.1021	0.09	[−0.11; 0.29]	1.8%	2.7%
SKIN	0.04	0.1254	0.04	[−0.21; 0.28]	1.2%	2.1%
STATIN	0.22	0.1211	0.22	[−0.01; 0.46]	1.3%	2.2%
SUA	0.16	0.1263	0.16	[−0.09; 0.41]	1.2%	2.1%
T2D	0.04	0.0651	0.04	[−0.09; 0.16]	4.5%	3.8%
T2DCan	−0.10	0.0948	−0.10	[−0.28; 0.09]	2.1%	2.9%
TEA	.	.			0.0%	0.0%
TELO	−0.25	0.1915	−0.25	[−0.63; 0.13]	0.5%	1.2%
VITD	0.15	0.1762	0.15	[−0.19; 0.50]	0.6%	1.3%
**Fixed effect model**			0.07	[0.04; 0.10]	100.0%	—
**Random effects model**			0.10	[0.05; 0.14]	—	100.0%

Heterogeneity: $I^2 = 51\%$, $\tau^2 = 0.0102$, $p < 0.01$

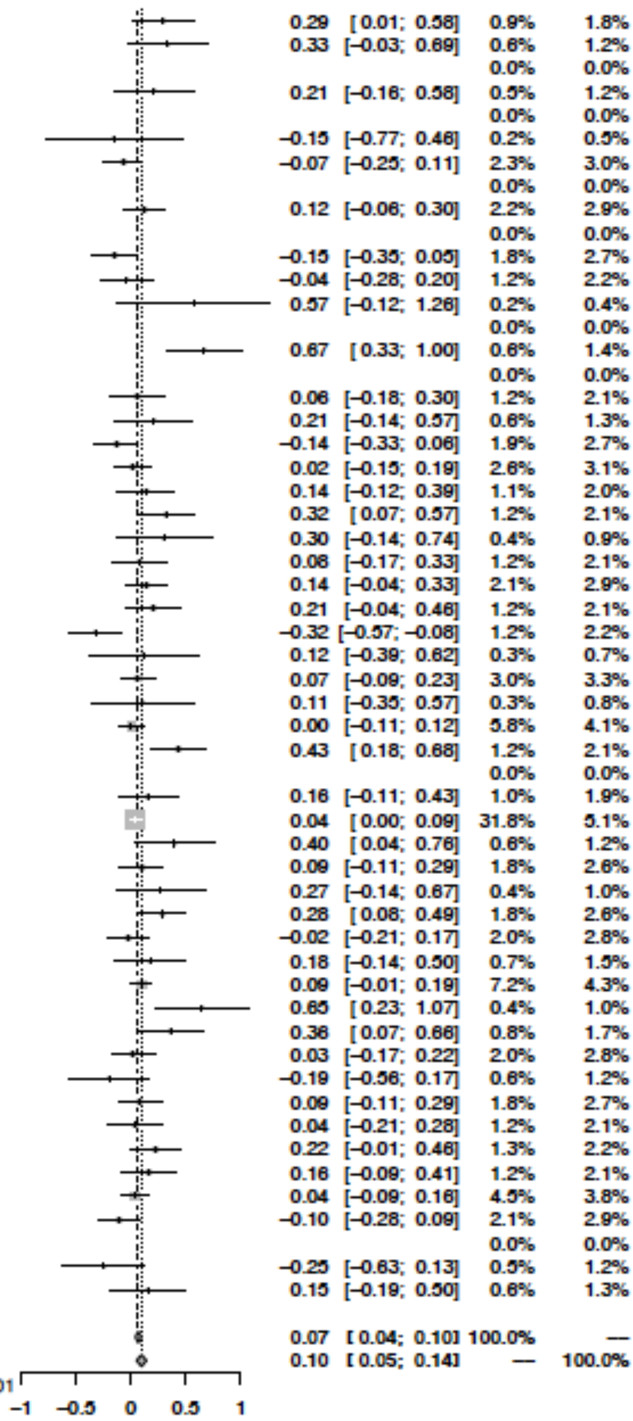

38) P-values < 0.001 and excess of significance bias

Study	TE	seTE		95%-CI	Weight (fixed)	Weight (random)
ADHD	0.18	0.1214	0.18	[−0.05; 0.42]	1.2%	2.0%
ADIP	−0.07	0.0398	−0.07	[−0.15; 0.01]	11.0%	5.0%
ADOPSY	0.18	0.3363	0.18	[−0.48; 0.84]	0.2%	0.4%
AFIB	−0.10	0.1528	−0.10	[−0.40; 0.20]	0.7%	1.5%
ALONE	.	.			0.0%	0.0%
ALS	−0.22	0.1930	−0.22	[−0.59; 0.16]	0.5%	1.0%
ANTIDEP	−0.05	0.1012	−0.05	[−0.25; 0.15]	1.7%	2.6%
ANTIPSY	.	.			0.0%	0.0%
ANXOCD	0.09	0.1092	0.09	[−0.13; 0.30]	1.5%	2.3%
ASPI	−0.18	0.2130	−0.18	[−0.59; 0.24]	0.4%	0.9%
ASPIcan	−0.24	0.2644	−0.24	[−0.75; 0.28]	0.2%	0.6%
AUTISM	0.04	0.1119	0.04	[−0.18; 0.26]	1.4%	2.3%
BIRTH	−0.08	0.1344	−0.08	[−0.34; 0.18]	1.0%	1.8%
BP	−0.36	0.2716	−0.36	[−0.90; 0.17]	0.2%	0.6%
CHOCO	.	.			0.0%	0.0%
CKDmort	−0.06	0.0561	−0.06	[−0.17; 0.05]	5.5%	4.3%
COFF	−0.06	0.1999	−0.06	[−0.45; 0.33]	0.4%	1.0%
COLO	−0.11	0.1773	−0.11	[−0.46; 0.23]	0.6%	1.2%
COLO2	0.25	0.1849	0.25	[−0.11; 0.61]	0.5%	1.1%
COPD	0.12	0.2825	0.12	[−0.43; 0.67]	0.2%	0.5%
CPROT	−0.00	0.0981	−0.00	[−0.20; 0.19]	1.8%	2.7%
DEM	0.01	0.1249	0.01	[−0.23; 0.26]	1.1%	2.0%
DEP	0.25	0.1181	0.25	[0.01; 0.48]	1.2%	2.1%
DEPmort	0.33	0.1995	0.33	[−0.06; 0.72]	0.4%	1.0%
EAT	−0.07	0.1641	−0.07	[−0.39; 0.25]	0.6%	1.3%
ENDO	0.09	0.1151	0.09	[−0.14; 0.31]	1.3%	2.2%
ENVT	0.26	0.2152	0.26	[−0.17; 0.68]	0.4%	0.8%
FIB	−0.17	0.0900	−0.17	[−0.35; 0.00]	2.1%	2.9%
FISH	−0.14	0.1154	−0.14	[−0.37; 0.08]	1.3%	2.2%
FLUOLD	−0.10	0.0903	−0.10	[−0.28; 0.07]	2.1%	2.9%
GASTRIC	−0.17	0.0659	−0.17	[−0.29; −0.04]	4.0%	3.8%
GESTA	−0.18	0.1187	−0.18	[−0.42; 0.05]	1.2%	2.1%
HAND	−0.50	0.3062	−0.50	[−1.10; 0.10]	0.2%	0.4%
HIV	0.10	0.1580	0.10	[−0.21; 0.41]	0.7%	1.4%
MENT	0.08	0.0328	0.08	[0.01; 0.14]	16.2%	5.3%
MET	0.05	0.2075	0.05	[−0.36; 0.45]	0.4%	0.9%
MG	−0.19	0.2359	−0.19	[−0.65; 0.27]	0.3%	0.7%
MS	−0.10	0.0970	−0.10	[−0.29; 0.09]	1.9%	2.7%
OBGYN	−0.06	0.0310	−0.06	[−0.12; 0.00]	18.1%	5.4%
PANC	−0.25	0.1424	−0.25	[−0.53; 0.03]	0.9%	1.6%
PARK	0.24	0.1656	0.24	[−0.09; 0.56]	0.6%	1.3%
PCan	−0.20	0.1244	−0.20	[−0.44; 0.05]	1.1%	2.0%
PHYACT	−0.52	0.1574	−0.52	[−0.83; −0.21]	0.7%	1.4%
PREEC	−0.15	0.1525	−0.15	[−0.44; 0.15]	0.7%	1.5%
PSY	0.04	0.1218	0.04	[−0.20; 0.28]	1.2%	2.0%
RHEU	0.18	0.1584	0.18	[−0.13; 0.49]	0.7%	1.4%
SCHIZO	0.39	0.1215	0.39	[0.15; 0.63]	1.2%	2.0%
SKIN	−0.21	0.1403	−0.21	[−0.48; 0.07]	0.9%	1.7%
STATIN	0.11	0.0729	0.11	[−0.03; 0.25]	3.3%	3.5%
SUA	0.05	0.1115	0.05	[−0.17; 0.26]	1.4%	2.3%
T2D	−0.08	0.0902	−0.08	[−0.26; 0.09]	2.1%	2.9%
T2DCan	0.04	0.1905	0.04	[−0.33; 0.42]	0.5%	1.0%
TELO	0.14	0.1066	0.14	[−0.07; 0.35]	1.5%	2.4%
VITD	0.06	0.1801	0.06	[−0.29; 0.42]	0.5%	1.1%
**Fixed effect model**			−0.02	[−0.05; 0.00]	100.0%	—
**Random effects model**			−0.02	[−0.06; 0.02]	—	100.0%

Heterogeneity: $I^2 = 44\%$, $\tau^2 = 0.0075$, $p < 0.01$

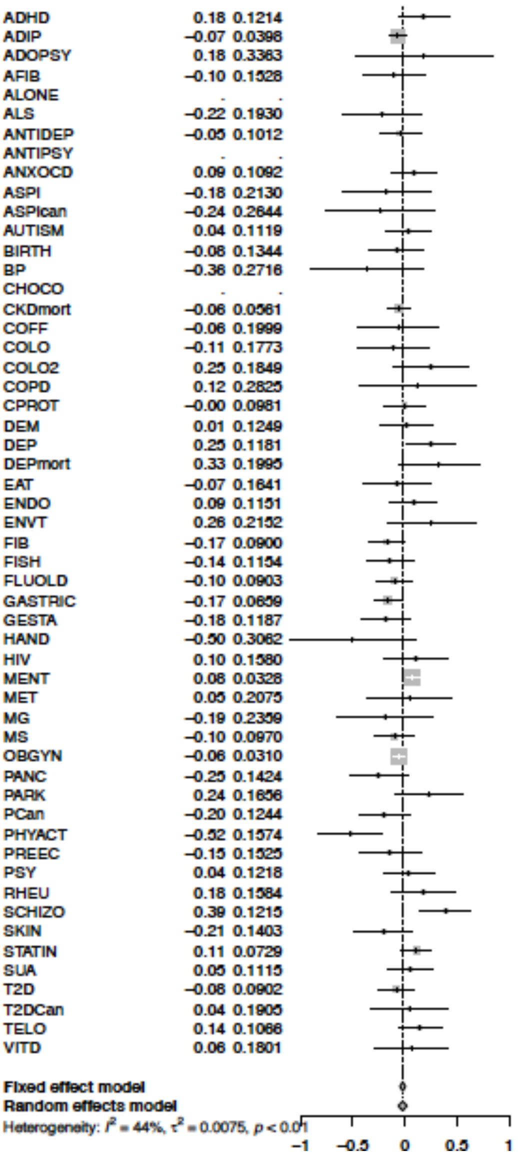

39) P-values < 0.001 and I^2 > 50%

Study	TE	seTE	95%-CI	Weight (fixed)	Weight (random)
ADHD	0.34	0.1331	0.34 [0.08; 0.60]	1.5%	2.0%
ADIP	−0.18	0.0997	−0.18 [−0.38; 0.01]	2.7%	2.7%
ADOPSY	0.14	0.2950	0.14 [−0.44; 0.72]	0.3%	0.6%
AFIB	−0.08	0.1291	−0.08 [−0.34; 0.17]	1.6%	2.0%
ALONE	0.12	0.1516	0.12 [−0.18; 0.41]	1.2%	1.6%
ALS	0.05	0.2956	0.05 [−0.53; 0.63]	0.3%	0.6%
ANTIDEP	0.01	0.1118	0.01 [−0.21; 0.22]	2.2%	2.4%
ANTIPSY	.	.		0.0%	0.0%
ANXOCD	0.11	0.1204	0.11 [−0.12; 0.35]	1.9%	2.2%
ASPI	−0.35	0.2014	−0.35 [−0.75; 0.04]	0.7%	1.1%
ASPIcan	−0.24	0.2644	−0.24 [−0.75; 0.28]	0.4%	0.7%
AUTISM	0.25	0.1325	0.25 [−0.01; 0.51]	1.5%	2.0%
BIRTH	0.03	0.1417	0.03 [−0.24; 0.31]	1.3%	1.8%
BP	0.00	0.3849	0.00 [−0.75; 0.75]	0.2%	0.4%
CHOCO	.	.		0.0%	0.0%
CKDmort	−0.00	0.1270	−0.00 [−0.25; 0.25]	1.7%	2.1%
COFF	−0.12	0.2353	−0.12 [−0.58; 0.34]	0.5%	0.8%
COLO	0.38	0.1668	0.38 [0.06; 0.71]	1.0%	1.4%
COLO2	0.13	0.1681	0.13 [−0.20; 0.46]	1.0%	1.4%
COPD	0.06	0.0609	0.06 [−0.06; 0.18]	7.3%	3.8%
CPROT	0.13	0.0961	0.13 [−0.06; 0.32]	2.9%	2.8%
DEM	0.15	0.1335	0.15 [−0.11; 0.42]	1.5%	1.9%
DEP	−0.07	0.0859	−0.07 [−0.24; 0.10]	3.7%	3.0%
DEPmort	0.11	0.2133	0.11 [−0.31; 0.52]	0.6%	1.0%
DEPREG	0.17	0.1683	0.17 [−0.16; 0.50]	1.0%	1.4%
EAT	−0.08	0.1422	−0.08 [−0.36; 0.20]	1.3%	1.8%
ENDO	−0.19	0.1257	−0.19 [−0.44; 0.05]	1.7%	2.1%
ENVT	0.26	0.1598	0.26 [−0.06; 0.57]	1.1%	1.5%
FIB	−0.14	0.2030	−0.14 [−0.54; 0.26]	0.7%	1.1%
FISH	0.20	0.1386	0.20 [−0.07; 0.47]	1.4%	1.9%
FLUOLD	0.06	0.1890	0.06 [−0.31; 0.43]	0.8%	1.2%
GASTRIC	0.33	0.0946	0.33 [0.14; 0.51]	3.0%	2.8%
GESTA	−0.06	0.1387	−0.06 [−0.33; 0.21]	1.4%	1.9%
HAND	.	.		0.0%	0.0%
HIV	−0.16	0.1224	−0.16 [−0.40; 0.08]	1.8%	2.2%
MENT	0.12	0.0631	0.12 [0.00; 0.25]	6.8%	3.7%
MET	−0.05	0.2047	−0.05 [−0.45; 0.35]	0.6%	1.1%
MG	−0.12	0.2413	−0.12 [−0.60; 0.35]	0.5%	0.8%
MS	−0.28	0.2029	−0.28 [−0.68; 0.11]	0.7%	1.1%
OBGYN	−0.11	0.0850	−0.11 [−0.27; 0.06]	3.7%	3.1%
PANC	0.16	0.1472	0.16 [−0.13; 0.45]	1.2%	1.7%
PARK	0.19	0.1658	0.19 [−0.14; 0.51]	1.0%	1.5%
PCan	0.09	0.0512	0.09 [−0.01; 0.19]	10.3%	4.1%
PHYACT	−0.62	0.1543	−0.62 [−0.93; −0.32]	1.1%	1.6%
PREEC	−0.23	0.1532	−0.23 [−0.53; 0.08]	1.2%	1.6%
PSY	−0.15	0.1084	−0.15 [−0.36; 0.06]	2.3%	2.5%
RHEU	−0.02	0.1027	−0.02 [−0.22; 0.18]	2.6%	2.6%
SCHIZO	0.18	0.0913	0.18 [0.00; 0.36]	3.2%	2.9%
SKIN	0.05	0.1180	0.05 [−0.18; 0.28]	1.9%	2.2%
STATIN	0.11	0.1227	0.11 [−0.13; 0.35]	1.8%	2.1%
SUA	0.02	0.0977	0.02 [−0.17; 0.21]	2.8%	2.7%
T2D	0.03	0.0767	0.03 [−0.12; 0.18]	4.6%	3.3%
T2DCan	0.13	0.1921	0.13 [−0.24; 0.51]	0.7%	1.2%
TEA	−0.17	0.1423	−0.17 [−0.45; 0.11]	1.3%	1.8%
TELO	0.33	0.1960	0.33 [−0.05; 0.72]	0.7%	1.1%
VITD	0.10	0.1824	0.10 [−0.25; 0.46]	0.8%	1.3%
Fixed effect model			0.04 [0.01; 0.07]	100.0%	—
Random effects model			0.03 [−0.02; 0.08]	—	100.0%

Heterogeneity: $I^2 = 43\%$, $\tau^2 = 0.0111$, $p < 0.01$

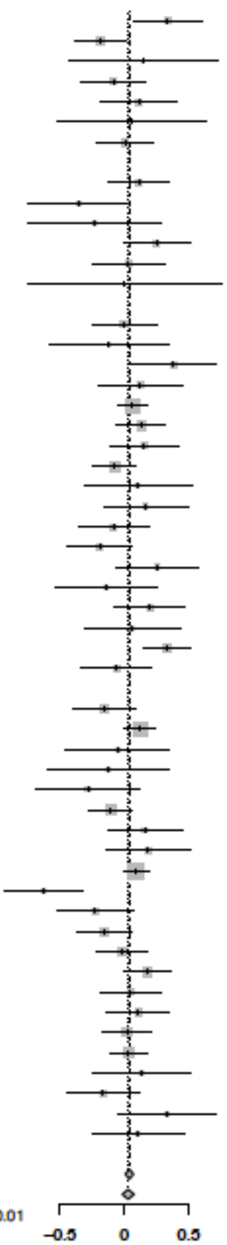

40) P-values < 0.001 and largest studies

Study	TE	seTE	95%-CI	Weight (fixed)	Weight (random)
ADHD	0.43	0.1411	0.43 [0.15; 0.70]	2.4%	2.6%
ADIP	0.05	0.1584	0.05 [−0.26; 0.36]	1.9%	2.2%
AFIB	0.48	0.1837	0.48 [0.12; 0.84]	1.4%	1.7%
ALONE	.	.		0.0%	0.0%
ALS	0.05	0.2956	0.05 [−0.53; 0.63]	0.5%	0.7%
ANTIDEP	0.40	0.1089	0.40 [0.19; 0.61]	4.0%	3.7%
ANTIPSY	.	.		0.0%	0.0%
ANXOCD	0.37	0.1007	0.37 [0.17; 0.57]	4.7%	4.0%
ASPIcan	0.82	0.1663	0.82 [0.50; 1.15]	1.7%	2.0%
AUTISM	0.24	0.1358	0.24 [−0.03; 0.50]	2.6%	2.7%
BIRTH	0.31	0.1385	0.31 [0.04; 0.58]	2.5%	2.6%
BP	.	.		0.0%	0.0%
CHOCO	.	.		0.0%	0.0%
COFF	0.38	0.2281	0.38 [−0.07; 0.83]	0.9%	1.2%
CPROT	0.50	0.0997	0.50 [0.31; 0.70]	4.8%	4.1%
DEM	0.36	0.1207	0.36 [0.12; 0.59]	3.3%	3.2%
DEPmort	0.24	0.2342	0.24 [−0.22; 0.70]	0.9%	1.1%
DEPREG	0.09	0.1440	0.09 [−0.20; 0.37]	2.3%	2.5%
EAT	0.39	0.1517	0.39 [0.10; 0.69]	2.1%	2.3%
ENDO	0.46	0.1200	0.46 [0.22; 0.69]	3.3%	3.2%
ENVT	0.01	0.1873	0.01 [−0.36; 0.38]	1.4%	1.6%
FIB	0.11	0.2233	0.11 [−0.33; 0.55]	1.0%	1.2%
FLUOLD	−0.08	0.1758	−0.08 [−0.43; 0.26]	1.6%	1.8%
GASTRIC	0.22	0.1082	0.22 [0.01; 0.44]	4.1%	3.7%
GESTA	0.33	0.1283	0.33 [0.08; 0.58]	2.9%	3.0%
HAND	.	.		0.0%	0.0%
MET	0.22	0.1956	0.22 [−0.16; 0.61]	1.3%	1.5%
MS	0.41	0.1507	0.41 [0.11; 0.70]	2.1%	2.3%
OBGYN	0.29	0.1051	0.29 [0.08; 0.50]	4.3%	3.8%
PANC	0.38	0.1292	0.38 [0.13; 0.63]	2.9%	2.9%
PARK	0.53	0.1431	0.53 [0.25; 0.82]	2.3%	2.5%
PCan	0.12	0.1203	0.12 [−0.11; 0.36]	3.3%	3.2%
PHYACT	0.03	0.2780	0.03 [−0.52; 0.57]	0.6%	0.8%
PREEC	0.39	0.1529	0.39 [0.09; 0.69]	2.0%	2.3%
PSY	0.30	0.1103	0.30 [0.08; 0.51]	3.9%	3.6%
SCHIZO	0.35	0.1141	0.35 [0.12; 0.57]	3.7%	3.5%
SKIN	0.14	0.1239	0.14 [−0.10; 0.39]	3.1%	3.1%
STATIN	0.26	0.1027	0.26 [0.06; 0.46]	4.5%	4.0%
SUA	0.09	0.1198	0.09 [−0.15; 0.32]	3.3%	3.2%
T2D	0.26	0.0912	0.26 [0.08; 0.44]	5.8%	4.5%
T2DCan	0.14	0.1741	0.14 [−0.20; 0.48]	1.6%	1.9%
TEA	0.04	0.2064	0.04 [−0.37; 0.44]	1.1%	1.4%
TELO	0.49	0.1523	0.49 [0.19; 0.79]	2.1%	2.3%
VITD	0.55	0.1786	0.55 [0.20; 0.90]	1.5%	1.8%
**Fixed effect model**			0.30 [0.26; 0.35]	100.0%	—
**Random effects model**			0.30 [0.25; 0.35]	—	100.0%

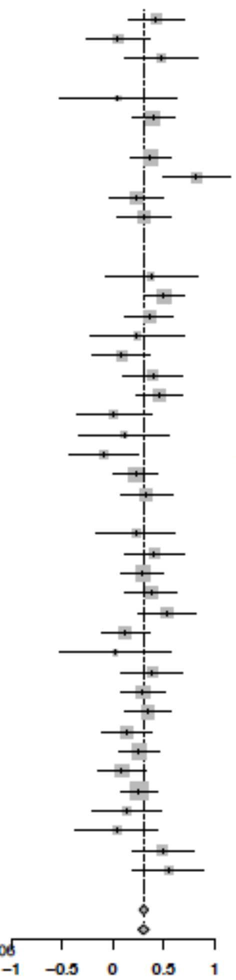

41) P-values < 0.001 and prediction intervals

Study	TE	seTE	95%-CI	Weight (fixed)	Weight (random)
ADHD	0.43	0.1160	0.43 [0.21; 0.66]	1.3%	1.8%
ADIP	0.07	0.0772	0.07 [−0.09; 0.22]	3.0%	2.9%
ADOPSY	0.33	0.2268	0.33 [−0.11; 0.78]	0.3%	0.6%
AFIB	0.17	0.1035	0.17 [−0.03; 0.37]	1.7%	2.1%
ALONE	0.55	0.3621	0.55 [−0.16; 1.26]	0.1%	0.3%
ALS	0.14	0.2950	0.14 [−0.44; 0.72]	0.2%	0.4%
ANTIDEP	0.24	0.0991	0.24 [0.04; 0.43]	1.8%	2.2%
ANTIPSY	.	.		0.0%	0.0%
ANXOCD	0.34	0.1017	0.34 [0.14; 0.54]	1.7%	2.2%
ASPI	−0.03	0.2203	−0.03 [−0.46; 0.40]	0.4%	0.7%
ASPIcan	0.14	0.2783	0.14 [−0.40; 0.69]	0.2%	0.4%
AUTISM	0.52	0.1051	0.52 [0.31; 0.72]	1.6%	2.1%
BIRTH	0.20	0.1237	0.20 [−0.04; 0.44]	1.2%	1.7%
BP	0.29	0.2738	0.29 [−0.25; 0.82]	0.2%	0.4%
CHOCO	.	.		0.0%	0.0%
CKDmort	0.23	0.0922	0.23 [0.05; 0.41]	2.1%	2.4%
COFF	0.23	0.1908	0.23 [−0.14; 0.61]	0.5%	0.8%
COLO	0.40	0.1673	0.40 [0.07; 0.73]	0.6%	1.1%
COLO2	0.10	0.0563	0.10 [−0.01; 0.21]	5.6%	3.8%
COPD	0.10	0.0871	0.10 [−0.07; 0.27]	2.4%	2.6%
CPROT	0.25	0.0559	0.25 [0.14; 0.36]	5.7%	3.8%
DEM	0.51	0.0986	0.51 [0.32; 0.70]	1.8%	2.3%
DEP	0.28	0.0595	0.28 [0.17; 0.40]	5.0%	3.7%
DEPmort	0.26	0.1443	0.26 [−0.02; 0.54]	0.9%	1.3%
DEPREG	0.37	0.1343	0.37 [0.11; 0.63]	1.0%	1.5%
EAT	0.31	0.1004	0.31 [0.11; 0.50]	1.8%	2.2%
ENDO	0.31	0.1105	0.31 [0.09; 0.53]	1.5%	1.9%
ENVT	0.40	0.1487	0.40 [0.10; 0.69]	0.8%	1.3%
FIB	0.20	0.1129	0.20 [−0.02; 0.42]	1.4%	1.9%
FISH	0.21	0.3282	0.21 [−0.43; 0.85]	0.2%	0.3%
FLUOLD	0.33	0.1604	0.33 [0.02; 0.65]	0.7%	1.1%
GASTRIC	0.35	0.0711	0.35 [0.21; 0.48]	3.5%	3.2%
GESTA	0.13	0.1250	0.13 [−0.11; 0.38]	1.1%	1.6%
HAND	0.18	0.1920	0.18 [−0.19; 0.56]	0.5%	0.8%
HIV	0.08	0.0417	0.08 [0.00; 0.16]	10.3%	4.5%
MENT	0.17	0.0501	0.17 [0.08; 0.27]	7.1%	4.1%
MET	0.18	0.1205	0.18 [−0.05; 0.42]	1.2%	1.7%
MG	−0.17	0.1273	−0.17 [−0.42; 0.08]	1.1%	1.6%
MS	0.40	0.2049	0.40 [0.00; 0.80]	0.4%	0.7%
OBGYN	0.33	0.0717	0.33 [0.19; 0.47]	3.5%	3.2%
PANC	0.35	0.1145	0.35 [0.13; 0.58]	1.4%	1.9%
PARK	0.28	0.1385	0.28 [0.01; 0.55]	0.9%	1.4%
PCan	0.27	0.1799	0.27 [−0.09; 0.62]	0.6%	0.9%
PHYACT	0.29	0.1707	0.29 [−0.05; 0.62]	0.6%	1.0%
PREEC	0.34	0.1054	0.34 [0.13; 0.54]	1.6%	2.1%
PSY	0.39	0.0945	0.39 [0.20; 0.57]	2.0%	2.4%
RHEU	0.11	0.0641	0.11 [−0.02; 0.23]	4.3%	3.5%
SCHIZO	0.35	0.0842	0.35 [0.18; 0.51]	2.5%	2.7%
SKIN	0.24	0.1004	0.24 [0.04; 0.43]	1.8%	2.2%
STATIN	0.27	0.0992	0.27 [0.08; 0.47]	1.8%	2.2%
SUA	0.17	0.1125	0.17 [−0.05; 0.39]	1.4%	1.9%
T2D	0.31	0.0600	0.31 [0.19; 0.42]	5.0%	3.7%
T2DCan	0.34	0.1511	0.34 [0.04; 0.64]	0.8%	1.2%
TEA	0.22	0.2752	0.22 [−0.32; 0.76]	0.2%	0.4%
VITD	0.34	0.1599	0.34 [0.03; 0.66]	0.7%	1.1%
**Fixed effect model**			0.23 [0.20; 0.26]	100.0%	—
**Random effects model**			0.25 [0.21; 0.29]	—	100.0%

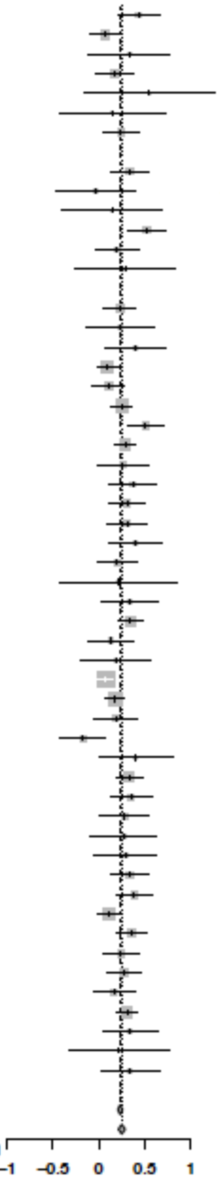

Heterogeneity: $I^2 = 39\%$, $\tau^2 = 0.0063$, $p < 0.01$

42) P-values < 0.001 and small study effects

Study	TE	seTE		95%-CI	Weight (fixed)	Weight (random)
ADHD	−0.12	0.1396	−0.12	[−0.39; 0.16]	1.6%	1.8%
ADIP	0.05	0.1557	0.05	[−0.26; 0.35]	1.3%	1.5%
ADOPSY	0.33	0.2268	0.33	[−0.11; 0.78]	0.6%	0.8%
AFIB	0.15	0.1804	0.15	[−0.21; 0.50]	0.9%	1.2%
ALONE	−0.43	0.3791	−0.43	[−1.17; 0.31]	0.2%	0.3%
ALS	0.05	0.2956	0.05	[−0.53; 0.63]	0.4%	0.5%
ANTIDEP	−0.05	0.1013	−0.05	[−0.25; 0.15]	3.0%	3.0%
ANTIPSY	.	.			0.0%	0.0%
ANXOCD	0.08	0.1139	0.08	[−0.14; 0.30]	2.4%	2.5%
ASPI	.	.			0.0%	0.0%
ASPIcan	0.12	0.2855	0.12	[−0.44; 0.68]	0.4%	0.5%
AUTISM	−0.20	0.1318	−0.20	[−0.46; 0.06]	1.8%	2.0%
BIRTH	−0.26	0.1015	−0.26	[−0.46; −0.06]	3.0%	3.0%
BP	−0.29	0.2121	−0.29	[−0.70; 0.13]	0.7%	0.9%
CHOCO	.	.			0.0%	0.0%
CKDmort	−0.24	0.1363	−0.24	[−0.51; 0.02]	1.7%	1.9%
COFF	−0.17	0.2082	−0.17	[−0.58; 0.24]	0.7%	0.9%
COLO	−0.32	0.1429	−0.32	[−0.60; −0.04]	1.5%	1.7%
COLO2	0.07	0.1492	0.07	[−0.22; 0.37]	1.4%	1.6%
COPD	−0.28	0.1592	−0.28	[−0.59; 0.03]	1.2%	1.4%
CPROT	−0.09	0.0952	−0.09	[−0.27; 0.10]	3.4%	3.3%
DEM	0.01	0.1249	0.01	[−0.23; 0.26]	2.0%	2.2%
DEP	−0.03	0.0945	−0.03	[−0.21; 0.16]	3.4%	3.3%
DEPmort	−0.12	0.2353	−0.12	[−0.58; 0.34]	0.6%	0.7%
DEPREG	−0.09	0.1479	−0.09	[−0.38; 0.20]	1.4%	1.6%
EAT	−0.11	0.1390	−0.11	[−0.38; 0.17]	1.6%	1.8%
ENDO	−0.05	0.1178	−0.05	[−0.28; 0.18]	2.2%	2.4%
ENVT	0.07	0.2236	0.07	[−0.37; 0.50]	0.6%	0.8%
FIB	−0.33	0.1076	−0.33	[−0.54; −0.12]	2.7%	2.7%
FISH	0.20	0.2840	0.20	[−0.36; 0.76]	0.4%	0.5%
FLUOLD	−0.04	0.2197	−0.04	[−0.47; 0.39]	0.6%	0.8%
GASTRIC	0.07	0.0898	0.07	[−0.10; 0.25]	3.8%	3.6%
GESTA	−0.17	0.1087	−0.17	[−0.38; 0.04]	2.6%	2.7%
HAND	0.33	0.2869	0.33	[−0.23; 0.90]	0.4%	0.5%
HIV	0.13	0.1718	0.13	[−0.21; 0.47]	1.0%	1.3%
MENT	−0.06	0.0509	−0.06	[−0.16; 0.04]	11.9%	6.7%
MET	−0.36	0.1769	−0.36	[−0.71; −0.02]	1.0%	1.2%
MS	−0.05	0.1824	−0.05	[−0.41; 0.31]	0.9%	1.1%
OBGYN	−0.06	0.0847	−0.06	[−0.23; 0.11]	4.3%	3.9%
PANC	0.02	0.1530	0.02	[−0.28; 0.32]	1.3%	1.5%
PARK	0.26	0.1677	0.26	[−0.07; 0.58]	1.1%	1.3%
PCan	0.15	0.0724	0.15	[0.00; 0.29]	5.9%	4.7%
PHYACT	−0.12	0.2753	−0.12	[−0.66; 0.41]	0.4%	0.5%
PREEC	−0.08	0.1431	−0.08	[−0.36; 0.20]	1.5%	1.7%
PSY	−0.04	0.1005	−0.04	[−0.23; 0.16]	3.0%	3.0%
RHEU	0.01	0.1677	0.01	[−0.32; 0.34]	1.1%	1.3%
SCHIZO	0.07	0.1201	0.07	[−0.17; 0.30]	2.1%	2.3%
SKIN	−0.27	0.1036	−0.27	[−0.48; −0.07]	2.9%	2.9%
STATIN	−0.02	0.1044	−0.02	[−0.22; 0.19]	2.8%	2.9%
SUA	−0.06	0.1373	−0.06	[−0.33; 0.21]	1.6%	1.9%
T2D	0.05	0.0906	0.05	[−0.13; 0.23]	3.7%	3.5%
T2DCan	−0.20	0.1256	−0.20	[−0.44; 0.05]	1.9%	2.1%
TEA	−0.17	0.1423	−0.17	[−0.45; 0.11]	1.5%	1.7%
TELO	−0.03	0.1746	−0.03	[−0.38; 0.31]	1.0%	1.2%
VITD	0.20	0.2092	0.20	[−0.21; 0.61]	0.7%	0.9%
**Fixed effect model**			−0.05	[−0.09; −0.02]	100.0%	—
**Random effects model**			−0.06	[−0.10; −0.02]	—	100.0%

Heterogeneity: $I^2 = 18\%$, $\tau^2 = 0.0036$, $p = 0.13$

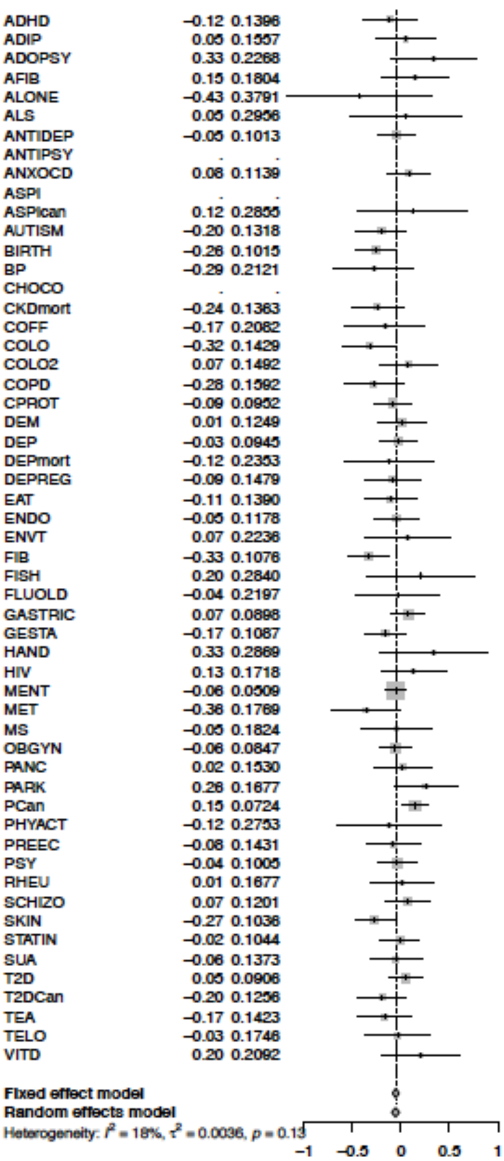

43) Small study effects and excess of significance bias

Study	TE	seTE		95%-CI	Weight (fixed)	Weight (random)
ADHD	0.15	0.1528	0.15	[−0.15; 0.45]	1.2%	2.2%
ADIP	−0.08	0.0477	−0.08	[−0.17; 0.01]	12.0%	3.2%
ADOPSY	−0.11	0.2754	−0.11	[−0.65; 0.43]	0.4%	1.2%
AFIB	0.53	0.1534	0.53	[0.23; 0.83]	1.2%	2.2%
ALONE	.	.			0.0%	0.0%
ALS	−0.17	0.1273	−0.17	[−0.42; 0.08]	1.7%	2.4%
ANTIDEP	0.45	0.1460	0.45	[0.17; 0.74]	1.3%	2.2%
ANTIPSY	.	.			0.0%	0.0%
ANXOCD	0.26	0.1446	0.26	[−0.02; 0.54]	1.3%	2.3%
ASPI	.	.			0.0%	0.0%
ASPIcan	−0.37	0.2571	−0.37	[−0.88; 0.13]	0.4%	1.3%
AUTISM	0.44	0.2150	0.44	[0.02; 0.87]	0.6%	1.6%
BIRTH	0.29	0.1590	0.29	[−0.02; 0.60]	1.1%	2.1%
BP	.	.			0.0%	0.0%
CHOCO	.	.			0.0%	0.0%
CKDmort	−0.06	0.0525	−0.06	[−0.16; 0.05]	9.9%	3.2%
COFF	0.46	0.2611	0.46	[−0.05; 0.97]	0.4%	1.3%
COLO	−0.24	0.1570	−0.24	[−0.55; 0.07]	1.1%	2.1%
COLO2	0.40	0.2263	0.40	[−0.04; 0.84]	0.5%	1.5%
COPD	0.61	0.2495	0.61	[0.12; 1.10]	0.4%	1.4%
CPROT	0.47	0.0909	0.47	[0.29; 0.65]	3.3%	2.8%
DEM	0.18	0.1418	0.18	[−0.10; 0.45]	1.4%	2.3%
DEP	0.09	0.1233	0.09	[−0.15; 0.33]	1.8%	2.5%
DEPmort	0.24	0.1901	0.24	[−0.13; 0.61]	0.8%	1.8%
EAT	0.34	0.1845	0.34	[−0.02; 0.70]	0.8%	1.9%
ENDO	−0.00	0.1433	−0.00	[−0.28; 0.28]	1.3%	2.3%
ENVT	−0.11	0.0887	−0.11	[−0.29; 0.06]	3.5%	2.9%
FIB	0.15	0.2326	0.15	[−0.30; 0.61]	0.5%	1.5%
FISH	.	.			0.0%	0.0%
FLUOLD	0.44	0.3037	0.44	[−0.16; 1.04]	0.3%	1.0%
GASTRIC	0.01	0.0678	0.01	[−0.13; 0.14]	6.0%	3.1%
GESTA	−0.25	0.0551	−0.25	[−0.35; −0.14]	9.0%	3.2%
HAND	.	.			0.0%	0.0%
HIV	0.17	0.1406	0.17	[−0.11; 0.44]	1.4%	2.3%
MENT	−0.00	0.0679	−0.00	[−0.14; 0.13]	5.9%	3.1%
MET	0.43	0.1822	0.43	[0.07; 0.79]	0.8%	1.9%
MS	−0.09	0.0851	−0.09	[−0.26; 0.07]	3.8%	2.9%
OBGYN	0.09	0.1173	0.09	[−0.14; 0.32]	2.0%	2.6%
PANC	0.05	0.1095	0.05	[−0.17; 0.26]	2.3%	2.6%
PARK	0.57	0.1408	0.57	[0.30; 0.85]	1.4%	2.3%
PCan	−0.23	0.0703	−0.23	[−0.37; −0.09]	5.5%	3.1%
PHYACT	0.25	0.2915	0.25	[−0.32; 0.82]	0.3%	1.1%
PREEC	0.00	0.1550	0.00	[−0.30; 0.30]	1.1%	2.2%
PSY	0.32	0.1407	0.32	[0.05; 0.60]	1.4%	2.3%
RHEU	0.34	0.1931	0.34	[−0.04; 0.72]	0.7%	1.8%
SCHIZO	0.39	0.1278	0.39	[0.14; 0.64]	1.7%	2.4%
SKIN	0.58	0.1422	0.58	[0.30; 0.86]	1.4%	2.3%
STATIN	0.01	0.1208	0.01	[−0.23; 0.25]	1.9%	2.5%
SUA	0.12	0.1170	0.12	[−0.11; 0.35]	2.0%	2.6%
T2D	0.18	0.0973	0.18	[−0.01; 0.37]	2.9%	2.8%
T2DCan	0.23	0.2633	0.23	[−0.29; 0.75]	0.4%	1.3%
TELO	0.19	0.2702	0.19	[−0.34; 0.72]	0.4%	1.2%
VITD	0.17	0.2450	0.17	[−0.31; 0.65]	0.5%	1.4%
**Fixed effect model**			0.04	[0.01; 0.07]	100.0%	—
**Random effects model**			0.14	[0.07; 0.22]	—	100.0%

Heterogeneity: $I^2 = 76\%$, $\tau^2 = 0.0406$, $p < 0.01$

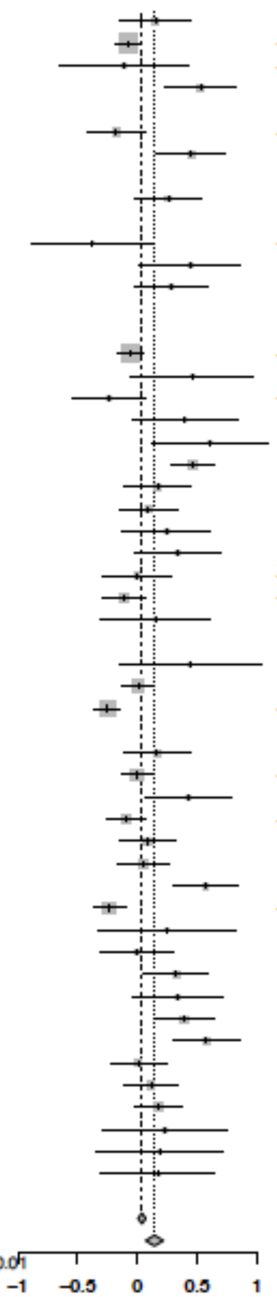

ADHD: Risk factor for attention deficit hyperactivity disorder ; ADIP: Adiposity and cancer outcomes; ADOPSY: Risk and protective factors for mental disorders with onset in childhood/adolescence; AFIB: Environmental factors and serum biomarkers for atrial fibrillation; ALONE: Factors associated to loneliness; ALS: Risk factor for amyotrophic lateral sclerosis; ANTIDEP: Antidepressant and adverse events; ANTIPSY: Antipsychotics and life-threatening events; ANXOCD: Risk and protective factors for anxiety and obsessive compulsive disorders; ASPI: Low-dose aspirin and health outcomes; ASPIcan: Aspirin and cancer outcomes; AUTISM: Environmental risk factors and biomarkers for autism spectrum disorder; BIRTH: Birth weight and later life events; BP: Environmental risk factors for bipolar disorder; CHOCO: Chocolate and health outcomes; CKDmort: Chronic kidney disease and mortality; COFF: Coffee and cancer risk; COLO: Risk factors for colorectal cancer metastasis and recurrence; COLO2: Non-genetic biomarkers and colorectal cancer risk; COPD: Risk factors for chronic obstructive pulmonary disease; CPROT: C-reactive protein and health outcomes; DEM: Environmental risk factors for dementia; DEP: Risk factors for depression; DEPmort: Depression and mortality; DEPPREG: Antidepressants during pregnancy and neonatal outcomes; EAT: Risk factors for eating

disorders; ENDO: Risk factors for endometrial cancer; ENVT: Environmental risk factors for obesity; FIB: Dietary Fiber and health outcomes; FISH: Fish and _-3 Fatty Acids consumptions and cancer outcomes; FLUOLD: Influenza vaccine in elderly and health outcomes; GASTRIC: Prognostic biomarkers for gastric cancer; GESTA: Risk factors for gestational diabetes; HAND: Handgrip strength and health outcomes; HIV: Human immunodeficiency virus infections and health outcomes; MENT: Peripheral biomarkers and major mental disorders; MET: Metformin and cancer outcomes; MG: Magnesium and health outcomes; MS: Environmental risk factors for multiple sclerosis; OBGYN: Obesity and gynecology/obstetric outcomes; PANC: Prognostic biomarkers for pancreatic ductal adenocarcinoma; PARK: Environmental risk factors and Parkinson's; PCan: Risk and protective factors for prostate cancer; PHYACT: Physical activity and cancer outcomes; PHYAF: Physical activity and atrial fibrillation outcomes; PREEC: Non-genetic risk factors for pre-eclampsia; PSY: Risk and protective factors for psychosis; RHEU: Environmental risk factors for rheumatic diseases; SCHIZO: Risk factors and peripheral biomarkers for schizophrenia spectrum disorders; SKIN: Non-genetic risk factors for skin cancer; STATIN: Statins and multiple non-cardiovascular outcomes; SUA: Serum uric acid and health outcomes; T2D: Risk factors for type 2 diabetes mellitus; T2DCan: Type 2 diabetes mellitus and cancer; TEA: Tea consumption and cancer; TELO: Telomere length and health outcomes; VITD: Vitamin D and health outcome
