# Supplementary material for: Validity of observational evidence on putative risk and protective factors: appraisal of 3744 meta-analyses on 57 topics
Source: BMC Med. 2021 Jul 6;19:157. doi: 10.1186/s12916-021-02020-6 (PMC8259334; doi:10.1186/s12916-021-02020-6)
Supplement: Supplementary file 7 — Additional file 7: Table 2. Comparisons of observational studies and RCTs. [file 12916_2021_2020_MOESM7_ESM.pdf]

Additional file 7: Table 2 Comparisons of observational studies and RCTs

Umbrella review	Outcome	Exposure	Comparison	OBS ES [95% CI]	RCT ES [95% CI]	OBS Pvalue	RCT Pvalue	P-value Het.	ROR [95% CI]	Direction	Concordance	OBS credibility assessment
**Yazhou 2018**	30-day mortality	Statins	30-day mortality	0.62 [0.50; 0.77]	0.93 [0.46; 1.90]	0.000	0.841	0.284	0.67 [0.26; 1.68]	protective	SIGN obs only	Suggestive
Statins and multiple non-cardiovascular outcomes	All-cause mortality	Statins	All-cause mortality	0.73 [0.64; 0.83]	0.98 [0.60; 1.59]	0.000	0.935	0.252	0.74 [0.40; 1.38]	protective	SIGN obs only	Suggestive
	Cancer	Statins	Cancer	0.85 [0.78; 0.92]	1.02 [0.97; 1.08]	0.000	0.470	0.000	0.83 [0.73; 0.95]	opposite	SIGN obs only	Suggestive
	Colorectal cancer	Statins	Colorectal cancer	0.91 [0.83; 1.00]	0.96 [0.85; 1.08]	0.047	0.504	0.490	0.95 [0.77; 1.17]	protective	SIGN obs only	Weak
	Hip fracture	Statins	Hip fracture	0.70 [0.51; 0.96]	1.23 [0.51; 2.97]	0.027	0.645	0.238	0.57 [0.17; 1.88]	opposite	SIGN obs only	Weak
	Gastric cancer	Statins	Gastric cancer	0.61 [0.39; 0.96]	0.83 [0.66; 1.05]	0.031	0.116	0.234	0.73 [0.37; 1.45]	protective	SIGN obs only	Weak
	Hematological cancer	Statins	Hematological cancer	0.88 [0.82; 0.95]	0.92 [0.77; 1.09]	0.001	0.347	0.644	0.96 [0.75; 1.22]	protective	SIGN obs only	Weak
	In-hospital mortality	Statins	In-hospital mortality	0.48 [0.24; 0.97]	0.98 [0.73; 1.33]	0.039	0.895	0.066	0.49 [0.18; 1.33]	protective	SIGN obs only	Weak
	Infection	Statins	Infection	0.57 [0.43; 0.75]	1.00 [0.96; 1.04]	0.000	1.000	0.000	0.57 [0.41; 0.78]	opposite	SIGN obs only	Suggestive
	Infection related mortality	Statins	Infection related mortality	0.78 [0.64; 0.95]	0.84 [0.67; 1.04]	0.014	0.120	0.623	0.93 [0.61; 1.41]	protective	SIGN obs only	Weak
	Liver cancer	Statins	Liver cancer	0.54 [0.34; 0.87]	0.95 [0.62; 1.45]	0.010	0.813	0.080	0.57 [0.23; 1.39]	protective	SIGN obs only	Weak
	Myopathy	Statins	Myopathy	2.63 [1.50; 4.61]	1.19 [0.88; 1.62]	0.001	0.264	0.015	2.21 [0.93; 5.26]	risk	SIGN obs only	Suggestive
	Pancreatitis	Statins	Pancreatitis	1.41 [1.15; 1.74]	0.77 [0.61; 0.97]	0.001	0.027	0.000	1.83 [1.18; 2.84]	opposite	SIGN rct & obs	Weak
	Prostate cancer	Statins	Prostate cancer	0.93 [0.87; 1.00]	1.06 [0.93; 1.20]	0.041	0.370	0.077	0.88 [0.72; 1.07]	opposite	SIGN obs only	Weak
	Sustained virological response	Statins	Sustained virological response	1.39 [1.22; 1.59]	1.24 [1.06; 1.46]	0.000	0.008	0.281	1.12 [0.84; 1.50]	protective	SIGN rct & obs	Suggestive
	ARDS mortality	Statins	ARDS mortality	1.05 [0.78; 1.40]	0.99 [0.78; 1.26]	0.744	0.935	0.760	1.06 [0.62; 1.81]	opposite	NONSIGN rct & obs	Non-significant
	Bladder cancer	Statins	Bladder cancer	1.11 [0.91; 1.35]	0.83 [0.63; 1.10]	0.300	0.190	0.095	1.34 [0.83; 2.15]	opposite	NONSIGN rct & obs	Non-significant
	Breast cancer	Statins	Breast cancer	1.01 [0.97; 1.06]	1.04 [0.78; 1.39]	0.660	0.790	0.844	0.97 [0.70; 1.36]	risk	NONSIGN rct & obs	Non-significant
	Gynecologic cancer	Statins	Gynecologic cancer	1.03 [0.94; 1.12]	0.97 [0.62; 1.50]	0.508	0.892	0.794	1.06 [0.63; 1.80]	opposite	NONSIGN rct & obs	Non-significant
	Kidney cancer	Statins	Kidney cancer	1.07 [0.96; 1.20]	1.01 [0.57; 1.79]	0.235	0.973	0.846	1.06 [0.53; 2.10]	risk	NONSIGN rct & obs	Non-significant

Umbrella review	Outcome	Exposure	Comparison	OBS ES [95% CI]	RCT ES [95% CI]	OBS Pvalue	RCT Pvalue	P-value Het.	ROR [95% CI]	Direction	Concordance	OBS credibility assessment
	Pancreatic cancer	Statins	Pancreatic cancer	1.05 [0.93; 1.19]	0.99 [0.45; 2.21]	0.438	0.980	0.886	1.06 [0.42; 2.66]	opposite	NONSIGN rct & obs	Non-significant
	Skin cancer	Statins	Skin cancer	0.88 [0.74; 1.05]	1.02 [0.82; 1.28]	0.152	0.862	0.307	0.86 [0.58; 1.28]	opposite	NONSIGN rct & obs	Non-significant
	Lung cancer	Statins	Lung cancer	0.86 [0.74; 1.00]	0.95 [0.85; 1.06]	0.050	0.362	0.296	0.91 [0.70; 1.18]	protective	SIGN obs only	Non-significant
	Femoral bone mineral density	Statins	Femoral bone mineral density	1.34 [0.83; 2.14]	1.99 [1.08; 3.68]	0.228	0.028	0.314	0.67 [0.23; 1.99]	protective	SIGN rct only	Non-significant
	Hip bone mineral density	Statins	Hip bone mineral density	1.49 [1.27; 1.75]	1.31 [0.19; 9.27]	0.000	0.785	0.899	1.14 [0.14; 9.43]	protective	SIGN obs only	Weak
	Spine bone mineral density	Statins	Spine bone mineral density	1.29 [1.00; 1.63]	1.11 [0.85; 1.49]	0.042	0.448	0.446	1.16 [0.68; 1.95]	protective	SIGN obs only	Weak
	Fracture	Statins	Fracture	0.97 [0.83; 1.12]	1.03 [0.91; 1.16]	0.690	0.633	0.542	0.94 [0.72; 1.24]	opposite	NONSIGN rct & obs	Non-significant
	Acute kidney injury	Statins	Acute kidney injury	1.13 [0.81; 1.57]	0.50 [0.31; 0.82]	0.469	0.005	0.007	2.26 [1.00; 5.12]	opposite	SIGN rct only	Non-significant
	Diabetes	Statins	Diabetes	1.44 [1.24; 1.67]	1.12 [1.04; 1.21]	0.000	0.003	0.003	1.29 [1.03; 1.61]	risk	SIGN rct & obs	Suggestive
	Cataract	Statins	Cataract	1.13 [1.01; 1.25]	0.89 [0.72; 1.10]	0.025	0.281	0.049	1.27 [0.92; 1.75]	opposite	SIGN obs only	Weak
	Ventilator free days	Statins	Ventilator free days	1.01 [0.96; 1.06]	1.04 [0.94; 1.14]	0.811	0.484	0.607	0.97 [0.84; 1.12]	protective	NONSIGN rct & obs	Non-significant
	Contrast-induced nephropathy	Statins	Contrast-induced nephropathy	0.61 [0.37; 1.00]	0.57 [0.47; 0.69]	0.051	0.000	0.803	1.07 [0.54; 2.13]	protective	SIGN rct only	Non-significant
**Theodoratou 2014**	Birth length	Vitamin D	Birth length	1.05 [0.93; 1.19]	1.15 [0.94; 1.40]	0.413	0.167	0.459	0.92 [0.67; 1.26]	protective	NONSIGN rct & obs	Non-significant
Vitamin D and health outcomes	CVD	VItamin D	CVD	0.66 [0.57; 0.77]	0.95 [0.86; 1.05]	0.000	0.314	0.000	0.69 [0.54; 0.89]	protective	SIGN obs only	Highly suggestive
	Small for gestational age	Vitamin D	Small for gestational age	0.54 [0.44; 0.67]	0.67 [0.40; 1.11]	0.000	0.124	0.444	0.81 [0.39; 1.66]	protective	SIGN obs only	Weak
	Birth weight; Low birth weight	Vitamin D	Birth weight; Low birth weight	1.12 [1.07; 1.17]	0.40 [0.23; 0.71]	0.000	0.001	0.000	2.80 [1.52; 5.15]	protective	SIGN rct & obs	Weak
	Head circumference at birth	Vitamin D	Head circumference at birth	1.02 [0.90; 1.15]	1.26 [1.03; 1.53]	0.735	0.021	0.075	0.81 [0.59; 1.11]	protective	SIGN rct only	Non-significant
	Mortality	Vitamin D	Mortality	0.86 [0.81; 0.92]	1.40 [0.38; 5.15]	0.000	0.613	0.464	0.61 [0.16; 2.41]	opposite	SIGN obs only	Suggestive
	All-cause mortality	VItamin D	All-cause mortality	0.72 [0.66; 0.78]	0.91 [0.82; 1.02]	0.000	0.090	0.001	0.79 [0.65; 0.96]	protective	SIGN obs only	Highly suggestive
	Fracture	Vitamin D	Fracture	0.31 [0.23; 0.42]	1.01 [0.93; 1.09]	0.000	0.806	0.000	0.31 [0.21; 0.45]	opposite	SIGN obs only	Weak

Umbrella review	Outcome	Exposure	Comparison	OBS ES [95% CI]	RCT ES [95% CI]	OBS Pvalue	RCT Pvalue	P-value Het.	ROR [95% CI]	Direction	Concordance	OBS credibility assessment
	Hip fracture	Vitamin D	Hip fracture	0.77 [0.72; 0.79]	1.15 [0.99; 1.33]	0.000	0.063	0.000	0.67 [0.55; 0.82]	opposite	SIGN obs only	Weak
**Kim 2020**	All-cause mortality	CKD	All-cause mortality	0.82 [0.76; 0.88]	0.99 [0.67; 1.46]	0.000	0.960	0.351	0.83 [0.52; 1.32]	protective	SIGN obs only	Suggestive
Chronic kidney disease and mortality	All-cause mortality	CKD	All-cause mortality	1.81 [1.47; 2.24]	0.92 [0.54; 1.46]	0.000	0.742	0.014	1.97 [0.97; 3.99]	opposite	SIGN obs only	Suggestive
	All-cause mortality	CKD	All-cause mortality	0.79 [0.71; 0.89]	0.99 [0.78; 1.27]	0.000	0.936	0.100	0.80 [0.56; 1.14]	protective	SIGN obs only	Weak
	All-cause mortality	CKD	All-cause mortality	0.90 [0.71; 1.15]	0.58 [0.36; 0.91]	0.392	0.021	0.099	1.55 [0.77; 3.14]	protective	SIGN rct only	Non-significant
	All-cause mortality	CKD	All-cause mortality	0.50 [0.42; 0.59]	0.76 [0.49; 1.17]	0.000	0.216	0.079	0.66 [0.36; 1.20]	protective	SIGN obs only	Suggestive
	All-cause mortality	CKD	All-cause mortality	0.67 [0.49; 0.93]	0.90 [0.80; 1.02]	0.014	0.089	0.091	0.74 [0.48; 1.16]	protective	SIGN obs only	Weak
	All-cause mortality	CKD	All-cause mortality	0.65 [0.57; 0.75]	1.13 [0.63; 2.03]	0.000	0.682	0.071	0.58 [0.28; 1.18]	opposite	SIGN obs only	Suggestive
	All-cause mortality	CKD	All-cause mortality	0.53 [0.32; 0.87]	1.55 [0.52; 4.62]	0.013	0.432	0.080	0.34 [0.07; 1.68]	opposite	SIGN obs only	Weak
	All-cause mortality	CKD	All-cause mortality	0.72 [0.64; 0.81]	0.86 [0.75; 0.99]	0.000	0.033	0.056	0.84 [0.65; 1.08]	protective	SIGN rct & obs	Weak
	All-cause mortality	CKD	All-cause mortality	0.61 [0.43; 0.86]	0.82 [0.53; 1.27]	0.005	0.373	0.298	0.74 [0.34; 1.63]	protective	SIGN obs only	Weak
**Veronese 2020**	Stroke	Low dose aspirin	Stroke	1.54 [0.91; 2.60]	0.81 [0.66; 0.99]	0.110	0.036	0.025	1.90 [0.92; 3.93]	opposite	SIGN rct only	Non-significant
Low-dose aspirin and health outcomes	Death	Low dose aspirin	Death	0.81 [0.60; 1.09]	0.95 [0.82; 1.12]	0.160	0.530	0.338	0.85 [0.54; 1.33]	protective	NONSIGN rct & obs	Non-significant
	AMI	Low dose aspirin	AMI	1.21 [0.63; 2.32]	0.68 [0.43; 1.07]	0.576	0.097	0.160	1.77 [0.58; 5.39]	opposite	NONSIGN rct & obs	Non-significant
	CVD death	Low dose aspirin	CVD death	0.93 [0.42; 2.05]	0.97 [0.71; 1.34]	0.859	0.871	0.917	0.96 [0.32; 2.90]	protective	NONSIGN rct & obs	Non-significant
	Death	Low dose aspirin	Death	1.58 [0.48; 5.18]	1.16 [0.41; 3.27]	0.447	0.776	0.700	1.36 [0.15; 12.55]	risk	NONSIGN rct & obs	Non-significant
	Re-operation for bleeding	Low dose aspirin	Re-operation for bleeding	1.23 [0.61; 2.48]	1.80 [1.03; 3.16]	0.558	0.039	0.404	0.68 [0.19; 2.40]	risk	SIGN rct only	Non-significant
	AMI	Low dose aspirin	AMI	1.27 [0.49; 3.27]	0.73 [0.37; 1.44]	0.625	0.365	0.354	1.74 [0.34; 8.88]	opposite	NONSIGN rct & obs	Non-significant
	Intra-cranial bleeding	Low dose aspirin	Intra-cranial bleeding	2.66 [0.56; 7.58]	1.29 [1.02; 1.63]	0.140	0.035	0.281	2.07 [0.45; 9.59]	risk	SIGN rct only	Non-significant
	Ischemic stroke	Low dose aspirin	Ischemic stroke	0.95 [0.56; 1.62]	0.93 [0.80; 1.08]	0.857	0.351	0.940	1.02 [0.52; 2.02]	protective	NONSIGN rct & obs	Non-significant

Umbrella review	Outcome	Exposure	Comparison	OBS ES [95% CI]	RCT ES [95% CI]	OBS Pvalue	RCT Pvalue	P-value Het.	ROR [95% CI]	Direction	Concordance	OBS credibility assessment
	Gastric cancer	Low dose aspirin	Gastric cancer	0.67 [0.31; 1.44]	0.74 [0.49; 1.14]	0.301	0.177	0.803	0.89 [0.27; 2.96]	protective	NONSIGN rct & obs	Non-significant
**Zhang 2020** Non-genetic biomarkers and colorectal cancer risk	Colorectal cancer	Vitamin E	Colorectal cancer	0.24 [0.05; 1.09]	0.89 [0.76; 1.05]	0.065	0.158	0.092	0.27 [0.05; 1.45]	protective	NONSIGN rct & obs	Non-significant
