# Supplementary material for: Validity of observational evidence on putative risk and protective factors: appraisal of 3744 meta-analyses on 57 topics
Source: BMC Med. 2021 Jul 6;19:157. doi: 10.1186/s12916-021-02020-6 (PMC8259334; doi:10.1186/s12916-021-02020-6)
Supplement: Supplementary file 8 — Additional file 8: Table 3. Comparisons of observational studies and MR studies. [file 12916_2021_2020_MOESM8_ESM.pdf]

Additional file 8: Table 3 Comparisons of observational studies and MR studies

Umbrella review	Outcome	Exposure	Comparison	OBS ES [95% CI]	MR ES [95% CI]	OBS Pvalue	MR Pvalue	P-value Het.	ROR [95% CI]	Direction	Concordance	OBS credibility assessment	Genetic
**Li 2018**	Atrial fibrillation	SUA	Hyper vs normal	1.49 [1.24; 1.79]	1.03 [0.93; 1.15]	0.000	0.585	0.001	1.45 [1.08; 1.93]	risk	SIGN obs only	Suggestive	Genetic risk score of 8 SUA related SNPs
Serum uric acid and health outcomes	Coronary heart diseases	SUA	Hyper vs normal	1.13 [1.05; 1.21]	1.05 [0.92; 1.18]	0.001	0.442	0.315	1.08 [0.89; 1.31]	risk	SIGN obs only	Suggestive	Genetic risk score of 31 SUA related SNPs
	Heart failure	SUA	Hyper vs normal	1.65 [1.41; 1.94]	1.07 [0.88; 1.30]	0.000	0.497	0.001	1.54 [1.08; 2.20]	risk	SIGN obs only	Highly suggestive	Genetic risk score of 14 SUA related SNPs
	Hypertension	SUA	Hyper versus normal	1.48 [1.33; 1.65]	0.98 [0.90; 1.06]	0.000	0.628	0.000	1.51 [1.25; 1.83]	opposite	SIGN obs only	Highly suggestive	Genetic risk score of 8 SUA related SNPs
	Cancer	SUA	Highest versus lowest	1.04 [0.99; 1.08]	0.95 [0.83; 1.08]	0.077	0.445	0.201	1.09 [0.92; 1.30]	opposite	NONSIGN mr & obs	Non-significant	Genetic risk score of 8 SUA related SNPs
	Chronic kidney diseases	SUA	1 mg/dl SUA increase	1.22 [1.16; 1.28]	1.20 [0.96; 1.50]	0.000	0.109	0.887	1.02 [0.77; 1.33]	risk	SIGN obs only	Highly suggestive	Genetic risk score of 8 SUA related SNPs
	Type 2 diabetes mellitus	SUA	1 mg/dl SUA increase	1.17 [1.09; 1.25]	0.95 [0.86; 1.05]	0.000	0.314	0.001	1.23 [1.04; 1.46]	opposite	SIGN obs only	Suggestive	Genetic risk score of 14 SUA related SNPs
	Diabetic macrovascular complications	SUA	Continuous or categorical SUA level	1.03 [1.00; 1.06]	1.18 [1.06; 1.33]	0.047	0.004	0.023	0.87 [0.76; 1.01]	risk	SIGN mr & obs	Weak	Genetic risk score of 3 SUA related SNPs
	Parkinson's disease	SUA	Hyper vs normal	0.65 [0.43; 0.97]	1.55 [1.10; 2.18]	0.038	0.012	0.001	0.42 [0.20; 0.89]	opposite	SIGN mr & obs	Weak	Genetic risk score of 8 SUA related SNPs
	Stroke/ ischemic stroke	SUA	Highest versus lowest	1.22 [1.02; 1.46]	0.99 [0.88; 1.12]	0.030	0.870	0.058	1.23 [0.91; 1.66]	opposite	SIGN obs only	Weak	Genetic risk score of 14 SUA related SNPs
**Kohler 2018**	Depression	Coffee consumption		0.71 [0.52; 0.99]	0.89 [0.66; 1.21]	0.037	0.451	0.317	0.80 [0.43; 1.49]	protective	SIGN obs only	Weak	Genome-wide significant SNPs#
Risk factors for depression	Depression	Coffee consumption		0.71 [0.52; 0.99]	0.95 [0.47; 1.91]	0.037	0.886	0.459	0.75 [0.27; 2.08]	protective	SIGN obs only		3 SNPs functionally relevant to coffee metabolism (rs4410790, rs2472297 and rs2470893)

Umbrella review	Outcome	Exposure	Comparison	OBS ES [95% CI]	MR ES [95% CI]	OBS Pvalue	MR Pvalue	P-value Het.	ROR [95% CI]	Direction	Concordance	OBS credibility assessment	Genetic
	Depression	Obesity		1.35 [1.21; 1.50]	0.84 [0.74; 1.14]	0.000	0.101	0.000	1.62 [1.17; 2.23]	risk	SIGN obs only	Suggestive	FTO rs3751812
	Depression	Obesity		1.35 [1.21; 1.50]	0.98 [0.90; 1.07]	0.000	0.663	0.000	1.38 [1.13; 1.68]	risk	SIGN obs only		wGRS from 32 SNPS associated to BMI in GWAS studies
	Depression	Smoking		1.68 [1.55; 1.82]	1.01 [0.96; 1.06]	0.000	0.694	0.000	1.66 [1.46; 1.89]	opposite*	SIGN obs only	Weak	CHRNA5 rs16969968 and CHRNA3rs1051730
	Depression	Smoking		1.68 [1.55; 1.82]	1.02 [0.96; 1.08]	0.000	0.510	0.000	1.65 [1.43; 1.89]	risk	SIGN obs only		CHRNA5 rs16969968 and CHRNA3rs1051730
	Depression	Smoking		1.68 [1.55; 1.82]	1.01 [0.95; 1.07]	0.000	0.743	0.000	1.66 [1.45; 1.91]	risk	SIGN obs only		CHRNA5 rs16969968 and CHRNA3rs1051730
	Depression	Smoking		1.68 [1.55; 1.82]	1.02 [0.88; 1.18]	0.000	0.791	0.000	1.65 [1.31; 2.07]	risk	SIGN obs only		CHRNA3 gene cluster rs1051730
	Depression	Smoking		1.68 [1.55; 1.82]	0.85 [0.66; 1.10]	0.000	0.212	0.000	1.98 [1.41; 2.76]	opposite	SIGN obs only		CHRNA3 gene cluster rs1051730
**Belbasis 2020**	Schizophrenia spectrum disorders	Cannabis use	Ever vs never; users vs non-users	3.90 [2.84; 5.35]	1.01 [0.93; 1.10]	0.000	0.816	0.000	3.86 [2.59; 5.76]	risk	SIGN obs only	Highly suggestive	GRS
Risk factors and peripheral biomarkers for schizophrenia spectrum disorders	Schizophrenia spectrum disorders	Cannabis use	Ever vs never; users vs non-users	3.90 [2.84; 5.35]	1.41 [1.09; 1.83]	0.000	0.009	0.000	2.77 [1.56; 4.92]	risk	SIGN mr & obs		GRS
	Schizophrenia spectrum disorders	Serum vitamin D	High level vs low levels; Per 10% increase	0.12 [0.04; 0.36]	0.99 [0.97; 1.02]	0.000	0.433	0.000	0.12 [0.04; 0.37]	protective	SIGN obs only	Weak	GRS
	Schizophrenia spectrum disorders	Serum CRP	High vs low levels; Highest vs lowest quartile	2.96 [1.74; 5.01]	1.40 [0.46; 4.25]	0.000	0.553	0.233	2.11 [0.41; 10.90]	protective	SIGN obs only	Suggestive	GRS
**Bellou 2018**	T2D	Alcohol consumption	Moderate vs never; Per	0.74 [0.67; 0.82]	1.02 [0.95; 1.09]	0.000	0.572	0.000	0.73 [0.61; 0.86]	opposite	SIGN obs only	Highly suggestive	Single variant (rs1229984)

Umbrella review	Outcome	Exposure	Comparison	OBS ES [95% CI]	MR ES [95% CI]	OBS Pvalue	MR Pvalue	P-value Het.	ROR [95% CI]	Direction	Concordance	OBS credibility assessment	Genetic
			units/week increase										
Risk factors for type 2 diabetes mellitus	Type 2 diabetes mellitus	Birth weight	Per 1 kg increase; Per 1 SD increase	0.80 [0.72; 0.88]	2.94 [1.70; 5.16]	0.000	0.000	0.000	0.27 [0.14; 0.52]	opposite	SIGN mr & obs	Suggestive	PRS 5 SNP
	Type 2 diabetes mellitus	BMI	Per 1 SD increase; Per 10 kg/m^2 increase	1.59 [1.40; 1.80]	19.40 [6.40; 59.10]	0.000	0.000	0.000	0.08 [0.02; 0.28]	risk	SIGN mr & obs	Highly suggestive	PRS 3 SNP
	Type 2 diabetes mellitus	BMI	Per 1 kg/m^2 increase	1.59 [1.40; 1.80]	1.39 [1.14; 1.68]	0.000	0.001	0.254	1.14 [0.83; 1.57]	risk	SIGN mr & obs		PRS 96 SNP
	Type 2 diabetes mellitus	BMI	Per 1 kg/m^2 increase	1.59 [1.40; 1.80]	1.35 [1.12; 1.62]	0.000	0.001	0.151	1.18 [0.86; 1.61]	risk	SIGN mr & obs		Single variant (rs9939609)
	Type 2 diabetes mellitus	BMI	Per 1 kg/m^2 increase	1.59 [1.40; 1.80]	1.27 [1.18; 1.36]	0.000	0.000	0.002	1.25 [1.03; 1.52]	risk	SIGN mr & obs		PRS 14 SNP
	T2D	Coffee consumption	Highest versus lowest; Per 1cup/day	0.70 [0.65; 0.75]	1.00 [0.99; 1.01]	0.000	1.000	0.000	0.70 [0.65; 0.76]	opposite	SIGN obs only	Highly suggestive	PRS 13 SNP
	Type 2 diabetes mellitus	Systolic blood pressure	Per 20 mmHg increase; Per 1 mmHg increase	1.75 [1.56; 1.97]	0.97 [0.95; 1.00]	0.000	0.020	0.000	1.80 [1.56; 2.08]	opposite	SIGN mr & obs	Highly suggestive	PRS 13 SNP
	Type 2 diabetes mellitus	Systolic blood pressure	Per 20 mmHg increase; Per 1 mmHg increase	1.75 [1.56; 1.97]	1.02 [1.01; 1.03]	0.000	0.000	0.000	1.72 [1.51; 1.95]	risk	SIGN mr & obs		PRS 6 SNP
	Type 2 diabetes mellitus	Serum adiponectin	Per 1 log µg/ml increase; Per 1 SD decrease	0.72 [0.67; 0.78]	0.86 [0.75; 0.99]	0.000	0.033	0.028	0.84 [0.68; 1.04]	opposite	SIGN mr & obs	Highly suggestive	PRS 3 SNP
	Type 2 diabetes mellitus	Serum adiponectin	Per 1 log µg/ml increase;	0.72 [0.67; 0.78]	0.94 [0.75; 1.19]	0.000	0.599	0.032	0.77 [0.56; 1.04]	protective	SIGN obs only		PRS 3 SNP

Umbrella review	Outcome	Exposure	Comparison	OBS ES [95% CI]	MR ES [95% CI]	OBS Pvalue	MR Pvalue	P-value Het.	ROR [95% CI]	Direction	Concordance	OBS credibility assessment	Genetic
			Per 1 SD decrease										
	Type 2 diabetes mellitus	Serum adiponectin	Per 1 log µg/ml increase; Per 1 SD decrease	0.72 [0.67; 0.78]	0.99 [0.95; 1.04]	0.000	0.663	0.000	0.73 [0.64; 0.82]	protective	SIGN obs only		PRS 3 SNP
	Type 2 diabetes mellitus	Serum CRP	Per 1 log pm/ml increase; Per 10-s% increase	1.26 [1.16; 1.37]	1.11 [0.94; 1.32]	0.000	0.228	0.189	1.14 [0.88; 1.46]	risk	SIGN obs only	Highly suggestive	PRS 4 SNP
	Type 2 diabetes mellitus	Serum CRP	Per 1 log pm/ml increase; Per 10-s% increase	1.26 [1.16; 1.37]	1.09 [0.95; 1.24]	0.000	0.205	0.070	1.16 [0.93; 1.44]	risk	SIGN obs only		PRS 18 SNP
	Type 2 diabetes mellitus	SUA	Per 1 mg/dl increase	1.17 [1.09; 1.25]	0.99 [0.92; 1.06]	0.000	0.781	0.001	1.18 [1.03; 1.36]	opposite	SIGN obs only	Suggestive	PRS 8 SNP
	Type 2 diabetes mellitus	SUA	Per 1 mg/dl increase	1.17 [1.09; 1.25]	0.83 [0.57; 1.23]	0.000	0.342	0.085	1.41 [0.90; 2.22]	opposite	SIGN obs only		PRS 8 SNP
	Type 2 diabetes mellitus	SUA	Per 1 mg/dl increase	1.17 [1.09; 1.25]	0.99 [0.94; 1.04]	0.000	0.697	0.000	1.18 [1.05; 1.33]	opposite	SIGN obs only		PRS 24 SNP
	Type 2 diabetes mellitus	Serum vitamin D	Highest versus lowest	0.62 [0.54; 0.70]	1.01 [0.86; 1.20]	0.000	0.907	0.000	0.61 [0.46; 0.83]	opposite	SIGN obs only	Convincing	PRS 5 SNP
	T2D	Waist circumferences	Per 1SD increase; Per 1 unit increase	1.66 [1.47; 1.88]	1.05 [1.01; 1.10]	0.000	0.025	0.000	1.58 [1.34; 1.87]	risk	SIGN mr & obs	Highly suggestive	PRS 5 SNP
**Markozannes 2020**	Venous thromboembolism	C reactive protein	per 1 SD lnCRP	1.14 [1.08; 1.19]	0.80 [0.56; 1.12]	0.000	0.207	0.047	1.42 [0.96; 2.12]	opposite	SIGN obs only	Convincing	rs3091244, rs1130864, rs1205, rs3093077
C-reactive protein and health outcomes	All-cause mortality	C reactive protein	High vs low	1.75 [1.55; 1.98]	1.08 [0.86; 1.34]	0.000	0.496	0.000	1.62 [1.15; 2.29]	risk	SIGN obs only	Highly suggestive	rs3091244, rs1130864, rs1205, rs3093077
	Coronary Heart Disease	C reactive protein	per 1 SD lnCRP	1.38 [1.27; 1.49]	1.00 [0.90; 1.13]	0.000	1.000	0.000	1.38 [1.14; 1.67]	risk	SIGN obs only	Highly suggestive	rs3093077, rs1205, rs1130864, rs1800947
	Coronary Heart Disease	C reactive protein	per 1 SD lnCRP	1.38 [1.27; 1.49]	1.00 [0.97; 1.02]	0.000	1.000	0.000	1.38 [1.24; 1.53]	risk	SIGN obs only		rs7553007, rs1130864, rs1205

Umbrella review	Outcome	Exposure	Comparison	OBS ES [95% CI]	MR ES [95% CI]	OBS Pvalue	MR Pvalue	P-value Het.	ROR [95% CI]	Direction	Concordance	OBS credibility assessment	Genetic
	Coronary Heart Disease	C reactive protein	per 1 SD lnCRP	1.38 [1.27; 1.49]	1.03 [0.99; 1.07]	0.000	0.136	0.000	1.34 [1.19; 1.51]	risk	SIGN obs only		rs1205
	Coronary Heart Disease	C reactive protein	per 1 SD lnCRP	1.38 [1.27; 1.49]	0.98 [0.94; 1.01]	0.000	0.270	0.000	1.41 [1.25; 1.58]	opposite	SIGN obs only		rs7553007
	Coronary Heart Disease	C reactive protein	per 1 SD lnCRP	1.38 [1.27; 1.49]	1.04 [0.61; 1.80]	0.000	0.887	0.311	1.33 [0.71; 2.47]	risk	SIGN obs only		rs6700896, rs4537545, rs7553007, rs1183910
	Type 2 diabetes	C reactive protein	High vs low	1.26 [1.16; 1.37]	1.11 [1.06; 1.17]	0.000	0.000	0.013	1.13 [0.99; 1.29]	risk	SIGN mr & obs	Highly suggestive	various SNPs
	Type 2 diabetes	C reactive protein	High vs low	1.26 [1.16; 1.37]	1.08 [0.78; 1.48]	0.000	0.638	0.361	1.17 [0.78; 1.75]	risk	SIGN obs only		various SNPs
	Type 2 diabetes	C reactive protein	High vs low	1.26 [1.16; 1.37]	1.21 [0.80; 1.82]	0.000	0.363	0.850	1.04 [0.64; 1.71]	risk	SIGN obs only		various SNPs
	Type 2 diabetes	C reactive protein	High vs low	1.26 [1.16; 1.37]	1.15 [0.93; 1.42]	0.000	0.195	0.431	1.10 [0.82; 1.47]	risk	SIGN obs only		rs1130864, rs1205, rs1800947, rs3093077
	Type 2 diabetes	C reactive protein	High vs low	1.26 [1.16; 1.37]	1.11 [0.94; 1.32]	0.000	0.228	0.189	1.14 [0.88; 1.46]	risk	SIGN obs only		various SNPs
	Type 2 diabetes	C reactive protein	High vs low	1.26 [1.16; 1.37]	1.09 [0.95; 1.24]	0.000	0.205	0.070	1.16 [0.93; 1.44]	risk	SIGN obs only		17 SNPs
	Type 2 diabetes	C reactive protein	High vs low	1.26 [1.16; 1.37]	0.95 [0.82; 1.10]	0.000	0.494	0.001	1.33 [1.05; 1.67]	opposite	SIGN obs only		Genetic risk score of 8 SUA related SNPs
	Hypertension	C reactive protein	High vs low	1.26 [1.13; 1.39]	1.03 [0.61; 1.73]	0.000	0.911	0.457	1.22 [0.65; 2.28]	risk	SIGN obs only	Suggestive	Genetic risk score of 31 SUA related SNPs
	Ischemic stroke	C reactive protein	per 1mg/L CRP	1.15 [1.09; 1.22]	1.19 [0.93; 1.53]	0.000	0.171	0.793	0.97 [0.71; 1.31]	risk	SIGN obs only	Suggestive	Genetic risk score of 14 SUA related SNPs
	Ischemic stroke	C reactive protein	per 1mg/L CRP	1.15 [1.09; 1.22]	1.06 [0.87; 1.29]	0.000	0.562	0.436	1.08 [0.84; 1.40]	risk	SIGN obs only		Genetic risk score of 8 SUA related SNPs
	Atrial fibrillation (recurrence)	C reactive protein	High vs low	4.05 [2.51; 6.54]	0.76 [0.62; 0.93]	0.000	0.008	0.000	5.33 [2.70; 10.54]	opposite	SIGN mr & obs	Weak	Genetic risk score of 8 SUA related SNPs
	Alzheimer's disease	C reactive protein	High vs low	1.21 [1.03; 1.42]	0.51 [0.30; 0.88]	0.020	0.015	0.003	2.37 [1.17; 4.78]	opposite	SIGN mr & obs	Weak	Genetic risk score of 8 SUA related SNPs
	Alzheimer's disease	C reactive protein	High vs low	1.21 [1.03; 1.42]	1.05 [0.88; 1.26]	0.020	0.596	0.250	1.15 [0.82; 1.62]	risk	SIGN obs only		Genetic risk score of 14 SUA related SNPs

Umbrella review	Outcome	Exposure	Comparison	OBS ES [95% CI]	MR ES [95% CI]	OBS Pvalue	MR Pvalue	P-value Het.	ROR [95% CI]	Direction	Concordance	OBS credibility assessment	Genetic
	Alzheimer's disease	C reactive protein	High vs low	1.21 [1.03; 1.42]	1.04 [0.94; 1.17]	0.020	0.482	0.127	1.16 [0.89; 1.52]	risk	SIGN obs only		Genetic risk score of 3 SUA related SNPs
	Alzheimer's disease	C reactive protein	High vs low	1.21 [1.03; 1.42]	0.36 [0.33; 0.40]	0.020	0.000	0.000	3.36 [2.60; 4.34]	risk	SIGN mr & obs		Genetic risk score of 8 SUA related SNPs
	Alzheimer's disease	C reactive protein	High vs low	1.21 [1.03; 1.42]	1.26 [0.89; 1.78]	0.020	0.191	0.835	0.96 [0.58; 1.59]	risk	SIGN obs only		Genetic risk score of 14 SUA related SNPs
	Alzheimer's disease	C reactive protein	High vs low	1.21 [1.03; 1.42]	1.26 [0.99; 1.61]	0.020	0.062	0.785	0.96 [0.64; 1.44]	risk	SIGN obs only		Genome-wide significant SNPs#
	Breast cancer	C reactive protein	High vs low	1.26 [1.06; 1.49]	1.05 [0.77; 1.43]	0.008	0.757	0.312	1.20 [0.74; 1.94]	risk	SIGN obs only	Weak	3 SNPs functionally relevant to coffee metabolism (rs4410790, rs2472297 and rs2470893)
	Colon cancer	C reactive protein	per unit lnCRP	1.12 [1.05; 1.21]	1.06 [0.98; 1.15]	0.002	0.153	0.313	1.06 [0.91; 1.23]	risk	SIGN obs only	Weak	FTO rs3751812
	Colorectal cancer	C reactive protein	per unit lnCRP	1.12 [1.05; 1.21]	1.04 [0.97; 1.12]	0.002	0.285	0.150	1.08 [0.93; 1.24]	risk	SIGN obs only		wGRS from 32 SNPS associated to BMI in GWAS studies
	Colorectal cancer	C reactive protein	per unit lnCRP	1.12 [1.05; 1.21]	1.10 [0.74; 1.64]	0.002	0.639	0.930	1.02 [0.64; 1.63]	risk	SIGN obs only		CHRNA5 rs16969968 and CHRNA3rs1051730
	Colorectal cancer	C reactive protein	per unit lnCRP	1.12 [1.05; 1.21]	1.74 [1.06; 2.85]	0.002	0.028	0.084	0.64 [0.37; 1.13]	risk	SIGN mr & obs		CHRNA5 rs16969968 and CHRNA3rs1051730
	Non-fatal Myocardial Infarction	C reactive protein	High vs low	1.37 [0.62; 3.00]	1.01 [0.74; 1.38]	0.434	0.950	0.481	1.36 [0.45; 4.07]	risk	NONSIGN mr & obs	Non-significant	CHRNA5 rs16969968 and CHRNA3rs1051730
	Prostate cancer	C reactive protein	per unit lnCRP	1.07 [0.98; 1.17]	1.02 [0.62; 1.69]	0.134	0.938	0.854	1.05 [0.58; 1.89]	risk	NONSIGN mr & obs	Non-significant	CHRNA3 gene cluster rs1051730
	Rectal cancer	C reactive protein	per unit lnCRP	1.03 [0.90; 1.17]	1.01 [0.91; 1.12]	0.659	0.851	0.818	1.02 [0.81; 1.29]	risk	NONSIGN mr & obs		CHRNA3 gene cluster rs1051730
**Zhang 2020**	Colorectal cancer	Vitamin E	Mean (μmol/L)	0.24 [0.05; 1.09]	0.94 [0.76; 1.17]	0.065	0.574	0.081	0.25 [0.04; 1.44]	protective	NONSIGN mr & obs	Non-significant	GRS
Non-genetic biomarkers and colorectal cancer risk	Colorectal cancer	25-hydroxyvitamin D	Highest with lowest categories	0.67 [0.54; 0.83]	0.91 [0.69; 1.19]	0.000	0.498	0.084	0.74 [0.45; 1.20]	protective	SIGN obs only	Suggestive	GRS

Umbrella review	Outcome	Exposure	Comparison	OBS ES [95% CI]	MR ES [95% CI]	OBS Pvalue	MR Pvalue	P-value Het.	ROR [95% CI]	Direction	Concordance	OBS credibility assessment	Genetic
	Colorectal cancer	Selenium	Highest vs. lowest category	0.86 [0.62; 1.18]	0.85 [0.75; 0.96]	0.358	0.010	0.947	1.01 [0.65; 1.58]	protective	SIGN mr only	Non-significant	GRS
	Colorectal cancer	Vitamin B12	Mean (pmol/L)	0.88 [0.78; 0.99]	1.21 [1.04; 1.42]	0.044	0.016	0.002	0.73 [0.55; 0.96]	opposite	SIGN mr & obs	Weak	GRS
	Colorectal cancer	Vitamin B6	Highest vs lowest categories (pmol/)	0.53 [0.38; 0.71]	1.04 [0.90; 1.20]	0.000	0.593	0.000	0.51 [0.32; 0.80]	opposite	SIGN obs only	Weak	Single variant (rs1229984)
	Colorectal cancer	Serum Zinc	Largest variation among serum levels	1.09 [0.00; 628.67]	0.94 [0.86; 1.03]	0.978	0.179	0.963	1.16 [0.00; 738.57]	protective	NONSIGN mr & obs	Non-significant	PRS 5 SNP
	Colorectal cancer	DHA 22:6n-3	Mean (% total fatty acids)	0.88 [0.57; 1.36]	1.32 [0.94; 1.87]	0.568	0.114	0.153	0.67 [0.31; 1.45]	opposite	NONSIGN mr & obs	Non-significant	PRS 3 SNP
	Colorectal cancer	EPA 20:5n-3	Mean (% total fatty acids)	0.17 [0.01; 5.19]	1.06 [0.91; 1.22]	0.309	0.436	0.294	0.16 [0.00; 5.67]	opposite	NONSIGN mr & obs	Non-significant	PRS 96 SNP
	Colorectal cancer	DPA 20:5n-3	Mean (% total fatty acids)	2.18 [0.61; 7.59]	1.58 [0.99; 2.52]	0.225	0.055	0.639	1.38 [0.25; 7.74]	opposite	NONSIGN mr & obs	Non-significant	Single variant (rs9939609)
	Colorectal cancer	Total cholesterol	Highest vs. lowest categories	1.11 [0.98; 1.27]	1.46 [1.20; 1.79]	0.115	0.000	0.024	0.76 [0.55; 1.06]	risk	SIGN mr only	Non-significant	PRS 14 SNP
	Colorectal cancer	Triglyceride	Highest vs. lowest categories	1.17 [0.99; 1.37]	0.98 [0.85; 1.12]	0.058	0.774	0.103	1.19 [0.88; 1.61]	opposite	NONSIGN mr & obs	Non-significant	PRS 13 SNP
	Colorectal cancer	LDL–cholesterol	Highest vs. lowest categories	1.04 [0.57; 1.92]	1.05 [0.92; 1.18]	0.899	0.442	0.976	0.99 [0.48; 2.06]	risk	NONSIGN mr & obs	Non-significant	PRS 13 SNP
	Colorectal cancer	HDL–cholesterol	Highest vs. lowest categories	0.83 [0.67; 1.04]	0.94 [0.84; 1.05]	0.097	0.277	0.322	0.88 [0.63; 1.23]	protective	NONSIGN mr & obs	Non-significant	PRS 6 SNP
	Colorectal cancer	C-reactive protein	1-unit change in ln (mg/l)	1.14 [1.04; 1.25]	1.04 [0.97; 1.12]	0.005	0.285	0.123	1.10 [0.93; 1.29]	risk	SIGN obs only	Weak	PRS 3 SNP
	Colorectal cancer	IL-6	1-unit change in ln (mg/l)	1.09 [0.85; 1.39]	0.98 [0.96; 1.00]	0.492	0.052	0.398	1.11 [0.85; 1.45]	opposite	NONSIGN mr & obs	Non-significant	PRS 3 SNP
	Colorectal cancer	Fasting glucose	Highest vs lowest	1.27 [1.11; 1.45]	1.04 [0.92; 1.18]	0.000	0.537	0.032	1.22 [0.94; 1.58]	protective	SIGN obs only	Highly suggestive	PRS 3 SNP

Umbrella review	Outcome	Exposure	Comparison	OBS ES [95% CI]	MR ES [95% CI]	OBS Pvalue	MR Pvalue	P-value Het.	ROR [95% CI]	Direction	Concordance	OBS credibility assessment	Genetic
			categories (mmol/L)										
	Colorectal cancer	HbA1C	Highest vs lowest categories (%)	1.25 [0.93; 1.67]	1.02 [0.85; 1.22]	0.135	0.830	0.247	1.23 [0.76; 1.97]	risk	NONSIGN mr & obs	Non-significant	PRS 4 SNP
	Colorectal cancer	IGF−I	Highest vs lowest categories (%)	1.31 [1.05; 1.63]	0.88 [0.76; 1.01]	0.016	0.078	0.003	1.49 [1.04; 2.14]	opposite	SIGN obs only	Weak	PRS 18 SNP
	Colorectal cancer	Adiponectin	Highest vs lowest categories (%)	0.78 [0.65; 0.95]	0.97 [0.91; 1.04]	0.010	0.371	0.034	0.80 [0.62; 1.04]	protective	SIGN obs only	Weak	PRS 8 SNP
	Colorectal cancer	Telomere length	Shortest Q4 vs longest Q2	1.02 [0.75; 1.40]	1.25 [0.92; 1.69]	0.901	0.150	0.360	0.82 [0.44; 1.51]	risk	NONSIGN mr & obs	Non-significant	PRS 8 SNP
