# Supplementary material for: Validity of observational evidence on putative risk and protective factors: appraisal of 3744 meta-analyses on 57 topics
Source: BMC Med. 2021 Jul 6;19:157. doi: 10.1186/s12916-021-02020-6 (PMC8259334; doi:10.1186/s12916-021-02020-6)
Supplement: Supplementary file 9 — Additional file 9: Figures 44 to 45. Forest plots of the ROR comparing meta-analyses of observational studies with meta-analyses of RCTs and with MR studies. [file 12916_2021_2020_MOESM9_ESM.pdf]

**Additional file 9: Figures 44 to 45 Forest plot of the ROR comparing meta-analyses of observational studies with**

## 44) Meta-analyses of RCTs

| Exposure – Outcome | logROR | selogROR | ROR | 95% CI |
|---|---|---|---|---|
| Statins – 30−day mortality | −0.41 | 0.4720 | 0.67 | [0.26; 1.68] |
| Statins – All−cause mortality | −0.29 | 0.3149 | 0.74 | [0.40; 1.38] |
| Statins – Cancer | −0.18 | 0.0695 | 0.83 | [0.73; 0.95] |
| Statins – Colorectal cancer | −0.05 | 0.1086 | 0.95 | [0.77; 1.17] |
| Statins – Hip fracture | −0.56 | 0.6108 | 0.57 | [0.17; 1.88] |
| Statins – Gastric cancer | −0.31 | 0.3482 | 0.73 | [0.37; 1.45] |
| Statins – Hematological cancer | −0.04 | 0.1262 | 0.96 | [0.75; 1.22] |
| Statins – In−hospital mortality | −0.71 | 0.5093 | 0.49 | [0.18; 1.33] |
| Statins – Infection | −0.56 | 0.1623 | 0.57 | [0.41; 0.78] |
| Statins – Infection related mortality | −0.07 | 0.2129 | 0.93 | [0.61; 1.41] |
| Statins – Liver cancer | −0.56 | 0.4564 | 0.57 | [0.23; 1.39] |
| Statins – Myopathy | 0.79 | 0.4421 | 2.21 | [0.93; 5.26] |
| Statins – Pancreatitis | 0.60 | 0.2240 | 1.83 | [1.18; 2.84] |
| Statins – Prostate cancer | −0.13 | 0.1005 | 0.88 | [0.72; 1.07] |
| Statins – Sustained virological response | 0.11 | 0.1492 | 1.12 | [0.84; 1.50] |
| Statins – ARDS mortality | 0.06 | 0.2716 | 1.06 | [0.62; 1.81] |
| Statins – Bladder cancer | 0.29 | 0.2428 | 1.34 | [0.83; 2.15] |
| Statins – Breast cancer | −0.03 | 0.1700 | 0.97 | [0.70; 1.36] |
| Statins – Gynecologic cancer | 0.06 | 0.2701 | 1.06 | [0.63; 1.80] |
| Statins – Kidney cancer | 0.06 | 0.3488 | 1.06 | [0.53; 2.10] |
| Statins – Pancreatic cancer | 0.06 | 0.4689 | 1.06 | [0.42; 2.66] |
| Statins – Skin cancer | −0.15 | 0.2029 | 0.86 | [0.58; 1.28] |
| Statins – Lung cancer | −0.10 | 0.1331 | 0.91 | [0.70; 1.18] |
| Statins – Femoral bone mineral density | −0.40 | 0.5541 | 0.67 | [0.23; 1.99] |
| Statins – Hip bone mineral density | 0.13 | 1.0805 | 1.14 | [0.14; 9.43] |
| Statins – Spine bone mineral density | 0.14 | 0.2678 | 1.16 | [0.68; 1.95] |
| Statins – Fracture | −0.06 | 0.1384 | 0.94 | [0.72; 1.24] |
| Statins – Acute kidney injury | 0.82 | 0.4170 | 2.26 | [1.00; 5.12] |
| Statins – Diabetes | 0.25 | 0.1146 | 1.29 | [1.03; 1.61] |
| Statins – Cararact | 0.24 | 0.1625 | 1.27 | [0.92; 1.75] |
| Statins – Ventilator free days | −0.03 | 0.0745 | 0.97 | [0.84; 1.12] |
| Statins – Contrast−induced nephropathy | 0.07 | 0.3516 | 1.07 | [0.54; 2.13] |
| Vitamin D – Birth length | −0.09 | 0.1627 | 0.92 | [0.67; 1.26] |
| VItamin D – CVD | −0.36 | 0.1276 | 0.69 | [0.54; 0.89] |
| Vitamin D – Small for gestational age | −0.22 | 0.3676 | 0.81 | [0.39; 1.66] |
| Vitamin D – Birth weight; Low birth weight | 1.03 | 0.3119 | 2.80 | [1.52; 5.15] |
| Vitamin D – Head circumference at birth | −0.21 | 0.1627 | 0.81 | [0.59; 1.11] |
| Vitamin D – Mortality | −0.49 | 0.6974 | 0.61 | [0.16; 2.41] |
| VItamin D – All−cause mortality | −0.23 | 0.0983 | 0.79 | [0.65; 0.96] |
| Vitamin D – Fracture | −1.18 | 0.1941 | 0.31 | [0.21; 0.45] |
| Vitamin D – Hip fracture | −0.40 | 0.1008 | 0.67 | [0.55; 0.82] |
| CKD – All−cause mortality | −0.19 | 0.2361 | 0.83 | [0.52; 1.32] |
| CKD – All−cause mortality | 0.68 | 0.3612 | 1.97 | [0.97; 3.99] |
| CKD – All−cause mortality | −0.23 | 0.1820 | 0.80 | [0.56; 1.14] |
| CKD – All−cause mortality | 0.44 | 0.3596 | 1.55 | [0.77; 3.14] |
| CKD – All−cause mortality | −0.42 | 0.3087 | 0.66 | [0.36; 1.20] |
| CKD – All−cause mortality | −0.30 | 0.2254 | 0.74 | [0.48; 1.16] |
| CKD – All−cause mortality | −0.55 | 0.3685 | 0.58 | [0.28; 1.18] |
| CKD – All−cause mortality | −1.07 | 0.8124 | 0.34 | [0.07; 1.68] |
| CKD – All−cause mortality | −0.18 | 0.1309 | 0.84 | [0.65; 1.08] |
| CKD – All−cause mortality | −0.30 | 0.3998 | 0.74 | [0.34; 1.63] |
| Low dose aspirin – Stroke | 0.64 | 0.3708 | 1.90 | [0.92; 3.93] |
| Low dose aspirin – Death | −0.16 | 0.2291 | 0.85 | [0.54; 1.33] |
| Low dose aspirin – AMI | 0.57 | 0.5672 | 1.77 | [0.58; 5.39] |
| Low dose aspirin – CVD death | −0.05 | 0.5654 | 0.96 | [0.32; 2.90] |
| Low dose aspirin – Death | 0.31 | 1.1327 | 1.36 | [0.15; 12.55] |
| Low dose aspirin – Re−operation for bleeding | −0.38 | 0.6417 | 0.68 | [0.19; 2.40] |
| Low dose aspirin – AMI | 0.55 | 0.8324 | 1.74 | [0.34; 8.88] |
| Low dose aspirin – Intra−cranial bleeding | 0.73 | 0.7826 | 2.07 | [0.45; 9.59] |
| Low dose aspirin – Ischemic stroke | 0.02 | 0.3477 | 1.02 | [0.52; 2.02] |
| Low dose aspirin – Gastric cancer | −0.11 | 0.6107 | 0.89 | [0.27; 2.96] |
| Vitamin E – Colorectal cancer | −1.31 | 0.8582 | 0.27 | [0.05; 1.45] |

Heterogeneity: $I^2 = 56\%$, $\tau^2 = 0.0452$, $p < 0.01$

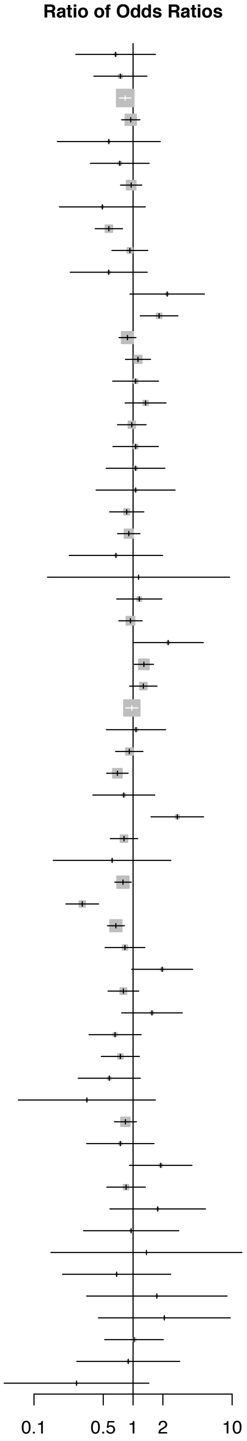

# 45) MR studies

| Exposure – Outcome – Genetic instrument | logROR | selogROR | ROR | 95% CI |
|---|---|---|---|---|
| SUA – Atrial fibrillation – Genetic risk score of 8 SUA related SNPs | 0.37 | 0.1478 | 1.45 | [1.08; 1.93] |
| SUA – Coronary heart diseases – Genetic risk score of 31 SUA related SNPs | 0.07 | 0.0997 | 1.08 | [0.89; 1.31] |
| SUA – Heart failure – Genetic risk score of 14 SUA related SNPs | 0.43 | 0.1809 | 1.54 | [1.08; 2.20] |
| SUA – Hypertension – Genetic risk score of 8 SUA related SNPs | 0.41 | 0.0967 | 1.51 | [1.25; 1.83] |
| SUA – Cancer – Genetic risk score of 8 SUA related SNPs | 0.09 | 0.0894 | 1.09 | [0.92; 1.30] |
| SUA – Chronic kidney diseases – Genetic risk score of 8 SUA related SNPs | 0.02 | 0.1390 | 1.02 | [0.77; 1.33] |
| SUA – Type 2 diabetes mellitus – Genetic risk score of 14 SUA related SNPs | 0.21 | 0.0859 | 1.23 | [1.04; 1.46] |
| SUA – Diabetic macrovascular complications – Genetic risk score of 3 SUA related SNPs | −0.14 | 0.0727 | 0.87 | [0.76; 1.01] |
| SUA – Parkinson's disease – Genetic risk score of 8 SUA related SNPs | −0.87 | 0.3820 | 0.42 | [0.20; 0.89] |
| SUA – Stroke/ ischemic stroke – Genetic risk score of 14 SUA related SNPs | 0.21 | 0.1530 | 1.23 | [0.91; 1.66] |
| Coffee consumption – Depression – Genome−wide significant SNPs# | −0.23 | 0.3189 | 0.80 | [0.43; 1.49] |
| Coffee consumption – Depression – 3 SNPs functionally relevant to coffee metabolism (rs4410790, rs2472297 and rs2470893) | −0.29 | 0.5219 | 0.75 | [0.27; 2.08] |
| Obesity – Depression – FTO rs3751812 | 0.48 | 0.1645 | 1.62 | [1.17; 2.23] |
| Obesity – Depression – wGRS from 32 SNPS associated to BMI in GWAS studies | 0.32 | 0.1007 | 1.38 | [1.13; 1.68] |
| Smoking – Depression – CHRNA5 rs16969968 and CHRNA3rs1051730 | 0.51 | 0.0662 | 1.66 | [1.46; 1.89] |
| Smoking – Depression – CHRNA5 rs16969968 and CHRNA3rs1051730 | 0.50 | 0.0710 | 1.65 | [1.43; 1.89] |
| Smoking – Depression – CHRNA5 rs16969968 and CHRNA3rs1051730 | 0.51 | 0.0713 | 1.66 | [1.45; 1.91] |
| Smoking – Depression – CHRNA3 gene cluster rs1051730 | 0.50 | 0.1158 | 1.65 | [1.31; 2.07] |
| Smoking – Depression – CHRNA3 gene cluster rs1051730 | 0.68 | 0.1713 | 1.98 | [1.41; 2.76] |
| Cannabis use – Schizophrenia spectrum disorders – GRS | 1.35 | 0.2044 | 3.86 | [2.59; 5.76] |
| Cannabis use – Schizophrenia spectrum disorders – GRS | 1.02 | 0.2937 | 2.77 | [1.56; 4.92] |
| Serum vitamin D – Schizophrenia spectrum disorders – GRS | −2.11 | 0.5733 | 0.12 | [0.04; 0.37] |
| Serum CRP – Schizophrenia spectrum disorders – GRS | 0.75 | 0.8370 | 2.11 | [0.41; 10.90] |
| Alcohol consumption – T2D – Single variant (rs1229984) | −0.32 | 0.0866 | 0.73 | [0.61; 0.86] |
| Birth weight – Type 2 diabetes mellitus – PRS 5 SNP | −1.30 | 0.3344 | 0.27 | [0.14; 0.52] |
| BMI – Type 2 diabetes mellitus – PRS 3 SNP | −2.50 | 0.6312 | 0.08 | [0.02; 0.28] |
| BMI – Type 2 diabetes mellitus – PRS 96 SNP | 0.13 | 0.1630 | 1.14 | [0.83; 1.57] |
| BMI – Type 2 diabetes mellitus – Single variant (rs9939609) | 0.16 | 0.1583 | 1.18 | [0.86; 1.61] |
| BMI – Type 2 diabetes mellitus – PRS 14 SNP | 0.22 | 0.1003 | 1.25 | [1.03; 1.52] |
| Coffee consumption – T2D – PRS 13 SNP | −0.36 | 0.0416 | 0.70 | [0.65; 0.76] |
| Systolic blood pressure – Type 2 diabetes mellitus – PRS 13 SNP | 0.59 | 0.0726 | 1.80 | [1.56; 2.08] |
| Systolic blood pressure – Type 2 diabetes mellitus – PRS 6 SNP | 0.54 | 0.0645 | 1.72 | [1.51; 1.95] |
| Serum adiponectin – Type 2 diabetes mellitus – PRS 3 SNP | −0.18 | 0.1096 | 0.84 | [0.68; 1.04] |
| Serum adiponectin – Type 2 diabetes mellitus – PRS 3 SNP | −0.27 | 0.1565 | 0.77 | [0.56; 1.04] |
| Serum adiponectin – Type 2 diabetes mellitus – PRS 3 SNP | −0.32 | 0.0619 | 0.73 | [0.64; 0.82] |
| Serum CRP – Type 2 diabetes mellitus – PRS 4 SNP | 0.13 | 0.1291 | 1.14 | [0.88; 1.46] |
| Serum CRP – Type 2 diabetes mellitus – PRS 18 SNP | 0.14 | 0.1104 | 1.16 | [0.93; 1.44] |
| SUA – Type 2 diabetes mellitus – PRS 8 SNP | 0.17 | 0.0711 | 1.18 | [1.03; 1.36] |
| SUA – Type 2 diabetes mellitus – PRS 8 SNP | 0.34 | 0.2311 | 1.41 | [0.90; 2.22] |
| SUA – Type 2 diabetes mellitus – PRS 24 SNP | 0.17 | 0.0607 | 1.18 | [1.05; 1.33] |
| Serum vitamin D – Type 2 diabetes mellitus – PRS 5 SNP | −0.49 | 0.1512 | 0.61 | [0.46; 0.83] |
| Waist circumferences – T2D – PRS 5 SNP | 0.46 | 0.0845 | 1.58 | [1.34; 1.87] |
| C reactive protein – Venous thromboembolism – rs3091244, rs1130864, rs1205, rs3093077 | 0.35 | 0.2016 | 1.42 | [0.96; 2.12] |
| C reactive protein – All−cause mortality – rs3091244, rs1130864, rs1205, rs3093077 | 0.48 | 0.1756 | 1.62 | [1.15; 2.29] |
| C reactive protein – Coronary Heart Disease – rs3093077, rs1205, rs1130864, rs1800947 | 0.32 | 0.0988 | 1.38 | [1.14; 1.67] |
| C reactive protein – Coronary Heart Disease – rs7553007, rs1130864, rs1205 | 0.32 | 0.0536 | 1.38 | [1.24; 1.53] |
| C reactive protein – Coronary Heart Disease – rs1205 | 0.29 | 0.0606 | 1.34 | [1.19; 1.51] |
| C reactive protein – Coronary Heart Disease – rs7553007 | 0.34 | 0.0591 | 1.41 | [1.25; 1.58] |
| C reactive protein – Coronary Heart Disease – rs6700896, rs4537545, rs7553007, rs1183910 | 0.28 | 0.3168 | 1.33 | [0.71; 2.47] |
| C reactive protein – Type 2 diabetes – various SNPs | 0.12 | 0.0683 | 1.13 | [0.99; 1.29] |
| C reactive protein – Type 2 diabetes – various SNPs | 0.15 | 0.2058 | 1.17 | [0.78; 1.75] |
| C reactive protein – Type 2 diabetes – various SNPs | 0.04 | 0.2521 | 1.04 | [0.64; 1.71] |
| C reactive protein – Type 2 diabetes – rs1130864, rs1205, rs1800947, rs3093077 | 0.09 | 0.1504 | 1.10 | [0.82; 1.47] |
| C reactive protein – Type 2 diabetes – various SNPs | 0.13 | 0.1291 | 1.14 | [0.88; 1.46] |
| C reactive protein – Type 2 diabetes – 17 SNPs | 0.14 | 0.1104 | 1.16 | [0.93; 1.44] |
| C reactive protein – Type 2 diabetes – NA | 0.28 | 0.1174 | 1.33 | [1.05; 1.67] |
| C reactive protein – Hypertension – rs1800947 | 0.20 | 0.3188 | 1.22 | [0.65; 2.28] |
| C reactive protein – Ischemic stroke – rs1130864, rs1205, rs1800947, rs3093077 | −0.03 | 0.1557 | 0.97 | [0.71; 1.31] |
| C reactive protein – Ischemic stroke – various SNPs | 0.08 | 0.1292 | 1.08 | [0.84; 1.40] |
| C reactive protein – Atrial fibrillation (recurrence) – rs1205, rs1130864, rs3091244, rs3093077 | 1.67 | 0.3477 | 5.33 | [2.70; 10.53] |
| C reactive protein – Alzheimer...s disease – 52 SNPs | 0.86 | 0.3587 | 2.37 | [1.17; 4.78] |
| C reactive protein – Alzheimer...s disease – rs2794520 | 0.14 | 0.1739 | 1.15 | [0.82; 1.62] |
| C reactive protein – Alzheimer...s disease – various SNPs | 0.15 | 0.1377 | 1.16 | [0.89; 1.52] |
| C reactive protein – Alzheimer...s disease – various SNPs | 1.21 | 0.1310 | 3.36 | [2.60; 4.34] |
| C reactive protein – Alzheimer...s disease – rs1130864, rs3093077 | −0.04 | 0.2587 | 0.96 | [0.58; 1.59] |
| C reactive protein – Alzheimer...s disease – various SNPs | −0.04 | 0.2060 | 0.96 | [0.64; 1.44] |
| C reactive protein – Breast cancer – rs1205, rs1130864, rs3091244, rs3093077 | 0.18 | 0.2448 | 1.20 | [0.74; 1.94] |
| C reactive protein – Colon cancer – various SNPs | 0.06 | 0.0770 | 1.06 | [0.91; 1.23] |
| C reactive protein – Colorectal cancer – various SNPs | 0.07 | 0.0729 | 1.08 | [0.93; 1.24] |
| C reactive protein – Colorectal cancer – rs1205, rs1130864, rs3091244, rs3093077 | 0.02 | 0.2392 | 1.02 | [0.64; 1.63] |
| C reactive protein – Colorectal cancer – rs1205, rs1800947, rs1130864, rs2808630, rs3093077 | −0.44 | 0.2885 | 0.64 | [0.37; 1.13] |
| C reactive protein – Non−fatal Myocardial Infarction – rs1130864 | 0.30 | 0.5612 | 1.36 | [0.45; 4.07] |
| C reactive protein – Prostate cancer – rs1205, rs1130864, rs3091244, rs3093077 | 0.05 | 0.3010 | 1.05 | [0.58; 1.89] |
| C reactive protein – Rectal cancer – various SNPs | 0.02 | 0.1199 | 1.02 | [0.81; 1.29] |
| Vitamin E – Colorectal cancer – unavailable | −1.37 | 0.8858 | 0.25 | [0.04; 1.44] |
| 25−hydroxyvitamin D – Colorectal cancer – unavailable | −0.31 | 0.2487 | 0.74 | [0.45; 1.20] |
| Selenium – Colorectal cancer – unavailable | 0.01 | 0.2271 | 1.01 | [0.65; 1.58] |
| Vitamin B12 – Colorectal cancer – unavailable | −0.32 | 0.1422 | 0.73 | [0.55; 0.96] |
| Vitamin B6 – Colorectal cancer – unavailable | −0.67 | 0.2329 | 0.51 | [0.32; 0.80] |
| Serum Zinc – Colorectal cancer – unavailable | 0.15 | 3.2920 | 1.16 | [0.00; 738.48] |
| DHA 22:6n−3 – Colorectal cancer – unavailable | −0.40 | 0.3971 | 0.67 | [0.31; 1.45] |
| EPA 20:5n−3 – Colorectal cancer – unavailable | −1.83 | 1.8201 | 0.16 | [0.00; 5.67] |
| DPA 20:5n−3 – Colorectal cancer – unavailable | 0.32 | 0.8802 | 1.38 | [0.25; 7.74] |
| Total cholesterol – Colorectal cancer – unavailable | −0.27 | 0.1681 | 0.76 | [0.55; 1.06] |
| Triglyceride – Colorectal cancer – unavailable | 0.18 | 0.1532 | 1.19 | [0.88; 1.61] |
| LDL...cholesterol – Colorectal cancer – unavailable | −0.01 | 0.3733 | 0.99 | [0.48; 2.06] |
| HDL...cholesterol – Colorectal cancer – unavailable | −0.12 | 0.1691 | 0.88 | [0.63; 1.23] |
| C−reactive protein – Colorectal cancer – unavailable | 0.09 | 0.0836 | 1.10 | [0.93; 1.29] |
| IL−6 – Colorectal cancer – unavailable | 0.11 | 0.1359 | 1.11 | [0.85; 1.45] |
| Fasting glucose – Colorectal cancer – unavailable | 0.20 | 0.1317 | 1.22 | [0.94; 1.58] |
| HbA1C – Colorectal cancer – unavailable | 0.20 | 0.2415 | 1.23 | [0.76; 1.97] |
| IGF...I – Colorectal cancer – unavailable | 0.40 | 0.1847 | 1.49 | [1.04; 2.14] |
| Adiponectin – Colorectal cancer – unavailable | −0.22 | 0.1309 | 0.80 | [0.62; 1.04] |
| Telomere length – Colorectal cancer – unavailable | −0.20 | 0.3144 | 0.82 | [0.44; 1.51] |

Heterogeneity: $I^2 = 88\%$, $\tau^2 = 0.0980$, $p < 0.01$

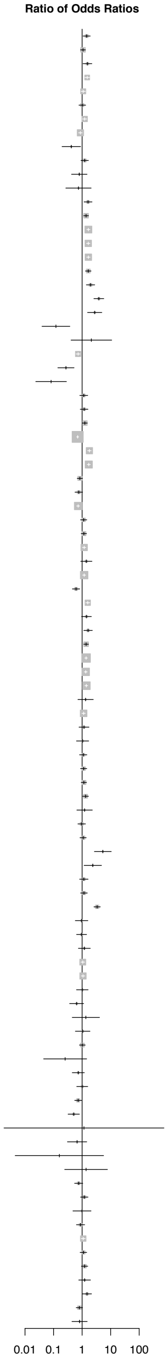


*A ROR < 1 indicates that the exposure is relatively more favorable in observational studies compared with RCTs or MR studies*

*In figure B, some associations such as, for example, coffee consumptions and depression, are assessed by more than one MR study using different genetic instruments*
